# Dissecting the Underlying Pharmaceutical Mechanism of Chinese Traditional Medicine Yun-Pi-Yi-Shen-Tong-Du-Tang Acting on Ankylosing Spondylitis through Systems Biology Approaches

**DOI:** 10.1038/s41598-017-13723-3

**Published:** 2017-10-18

**Authors:** Duoli Xie, Lin Huang, Guanghui Zhao, Yiran Yu, Jiawei Gao, Haichang Li, Chengping Wen

**Affiliations:** 1 TCM Clinical Basis Institute, Zhejiang Chinese Medicine University, 548 Binwen Road, Hangzhou, Zhejiang, 310000 China; 2 Guangzhou University of Chinese Medicine, Mathematical Engineering Academy of Chinese Medicine, Guangzhou, 510006 China

## Abstract

Traditional Chinese Medicine (TCM) has been served as complementary medicine for Ankylosing Spondylitis (AS) treatment for a long time. Yun-Pi-Yi-Shen-Tong-Du-Tang (Y-Y-T) is a novel empirical formula designed by Prof. Chengping Wen. In this study, a retrospective investigation supported efficacy of Y-Y-T and then we deciphered the underlying molecular mechanism of the efficacy. Herbal ingredients and targeting proteins were collected from TCMID. PPI networks were constructed to further infer the relationship among Y-Y-T, drugs used for treating AS, differentially expressed genes of AS patients and AS disease proteins. Finally, it was suggested that TLR signaling pathway and T cell receptor signaling pathway may involve in the biological processes of AS progression and contribute to the curative effect and proteins such as JAK2, STAT3, HSP90AA1, TNF and PTEN were the key targets. Our systemic investigation to infer therapeutic mechanism of Y-Y-T for AS treatment provides a new insight in understanding TCM pharmacology.

## Introduction

Ankylosing Spondylitis (AS) is a chronic rheumatic disease with 0.2–0.5% prevalence worldwide^1^. It mainly affects the axial skeleton and is characterized by morning stiffness and sacroiliitis^2^. In the course of AS development, the onset of joint fusion, spinal deformity and disability will sequentially take place^3^. So far, the pathogenesis of AS is still not clear. It was reported that the etiopathogenesis was related to Gram-negative bacteria, human leukocyte antigen B27 (HLA-27), pattern recognition receptors (PRRs) and inflammatory bowel disease (IBD)^4,5^. Medication treatment for AS mainly includes the following three classes of drugs, non-steroid anti-inflammatory drugs (NSAIDs), disease-modifying anti-rheumatic drugs (DMARDs) and biologicals. These medical treatments can alleviate inflammatory reaction, relieve pain of sacroiliac joints and spine, slow down disease progression and decrease disease activity to some extent. But a prolonged therapy with these medications may also cause systematic side effects such as serious infections and gastrointestinal intolerance^6,7^.

Traditional Chinese Medicine (TCM), as an alternative medicine, is widely used for AS treatment in clinical in Asian. For example, kunxian capsule, a Chinese patent medicine used for immunologic treatments, can effectively induce an anti-inflammatory effect and immunologic regulation^8^. Besides, clinical observation found that Chinese herbs combined with etanercept could relieve various symptoms and improve percentage of effectiveness in AS patients^9^. Another clinical case reported that Bushen-Qiangdu-Zhilv Decoction can help AS patients alleviate the inflammatory symptoms and improve quality of life^3^.

Yun-Pi-Yi-Shen-Tong-Du-Tang (Y-Y-T) is an empirical TCM formula created by Prof. Chengping Wen and it followed an academic theory of invigorating spleen and kidney dredge collaterals which was advanced by the national medical master Zhu Liang Chun. In the passing 21 years, as a rheumatologist, Prof. Wen made continual readjustment to the original compatibility of Chinese herbs and finally found a compatibility with stable curative effect and low side effect, namely Y-Y-T. It contains 11 medicinal herbs with recommended doses as follows: *Dioscoreae Nipponicae Rhizoma* (Chuan Shan Long, 20 g), *Atractylodes Lancea* (Cang Zhu, 12 g), *Rhizoma Smilacis Glabrae* (Tu Fu Ling, 30 g), *Lonicera Japonica* (Jin Yin Hua, 15 g), *Achyranthes Bidentata* (Niu Xi, 12 g), *Myrrh* (Mo Yao, 10 g), *Aconitum Carmichaeli* (Chuan Wu, 10 g), *Radix Astragali* (Huang Qi, 15 g), *Glycyrrhiza* (Gan Cao, 6 g), *Leech* (Shui Zhi, 6 g), *Coptis* (Huang Lian, 9 g). Studies have shown that *Myrrh, Coptis* and *Lonicera Japonica* were remedies for inflammation related disorders^10–12^. *Aconitum Carmichaeli* is an analgesic and anti-rheumatic medicine which can effectively alleviate the symptoms of neuropathic pain and inflammatory^13^ while *Dioscoreae Nipponicae Rhizoma* has been widely used to deal with arthroncus, arthrodynia and arthritis^14^.

Owing to the boost of biomedical data, system biology, polypharmacology and system biology-based network pharmacology are booming in recent years. The first case of network pharmacology-based TCM study of *Qing- Luo-Yin* ^15^ give us a deeper understanding of the mechanisms of TCM and broaden us the notion of drug discovery which facilitated a new research direction of “multiple targets, multiple effects, complex diseases”. Ke *et al*.^16^ by employing molecular docking and network analysis, filtered 504 herbs highly related to neurodegenerative diseases from 7362 kinds of herbs. Further, Luo *et al*.^17^ created herbs-compounds-targets-pathway-cooperation networks to explain the potential mechanism of Danggui-shaoyao-san in addressing neurodegenerative diseases. Besides, Liang *et al*. successfully designed a novel effective TCM formula by conducting systematic analysis for herbs^18^.

AS is a disease with complex pathogenic factors. Y-Y-T showed its own worth in the intervention of disease progression. To clarify the underlying mechanism of Y-Y-T in AS treatment, we deeply analyzed the composition of the formula and constructed PPI networks to show the interrelationship between formula targets and AS-related proteins and to predict potential key targets of Y-Y-T. This systematic approach to uncover the mechanism of therapy on molecular level facilitates our understanding of the intangible biological processes of this formula.

## Results

### Clinical performance of Y-Y-T in AS patients’ treatment

We made a retrospective investigation to evaluate the effectiveness of the formula. Inflammatory markers of acute phase reactants C-reactive protein (CRP) and erythrocyte sedimentation rate (ESR) were recorded. Bath Ankylosing Spondylitis Disease Activity Index (BASDAI)^19^, Ankylosing Spondylitis Disease Activity Score (ASDAS)-CRP and ASDAS-ESR were calculated to appraise the disease activity and curative effect. Among 30 patients (Supplement Tables 1–3 17 patients were dignosed for active period of AS (BASDAI score of ≥ 4) at baseline before treatment. After the 6-month treatment, the level of BASDAI, ASDAS-CRP and ASDAS-ESR were significantly decreased (p-value as 5.02e-09, 1.15e-07 and 1.61e-12 respectively). Moreover, symptoms such as pain, fatigue and morning stiffness were apparently relieved. The result of clinical research supported the efficacy of Y-Y-T in alleviating symptoms and enhancing physical quality for AS patients.

### The herbs, ingredients and targets of the Y-Y-T

It is generally recognized that spleen, kidneys and governor vessel, in the cognition of TCM, are closely related to the risk of AS progression. So, the creative principle of Y-Y-T is to strengthen spleen, nourish kidney and dredge governor vessel. Specifically, *Atractylodes Lancea, Radix Astragali* and *Glycyrrhiza* are used to strengthen the spleen, *Achyranthes Bidentata* is added to burst the function of kidneys while *Rhizoma Smilacis Glabrae* and *Aconitum Carmichaeli* are used to dredge governor vessel. The six herbs above play vital roles in the treatment and thus being called as Jun (Monarch) while the other 5 herbs are served as assistance. To reveal the underlying treatment mechanism of this formula, we collected herbal ingredients from TCMID and got 357 kinds of ingredients (Supplement Table S4). To be detailed, *Aconitum Carmichaeli* contained 2 ingredients, gaconitine and songorine. *Myrrh* contained 2 ingredients, eugenol and cinnamaldehyde. *Dioscoreae Nipponicae Rhizoma* contained 5 ingredients, including dioscin, trillin, allantoin, etc. There were 68, 33, 106, 2, 70, 11, 35 and 30 ingredients in *Atractylodes Lancea*, *Rhizoma Smilacis Glabrae*, *Lonicera Japonica*, *Achyranthes Bidentata*, *Radix Astragali*, *Glycyrrhiza*, *Leech* and *Coptis* respectively. Function of some ingredients has been studied. For instance, discin, one ingredients of *Dioscorea nipponica*, could repair the damaged synovium tissue by reducing Th1/Th2^20^, regulate the signaling pathway of the microRNA let7i/TLR4/MyD88 and reverse the inflammatory kidney injury^21^. Astilbin, a bioactive compound extracted from *Rhizoma Smilacis Glabrae*, could decrease antigen-specific autoantibodies by up-regulating regulatory T cells and down-regulating Th17 cells and it also can reduce the efficiency of antigen presenting cells by decreasing the expression of MHC class II^22^.

**Figure 1:**
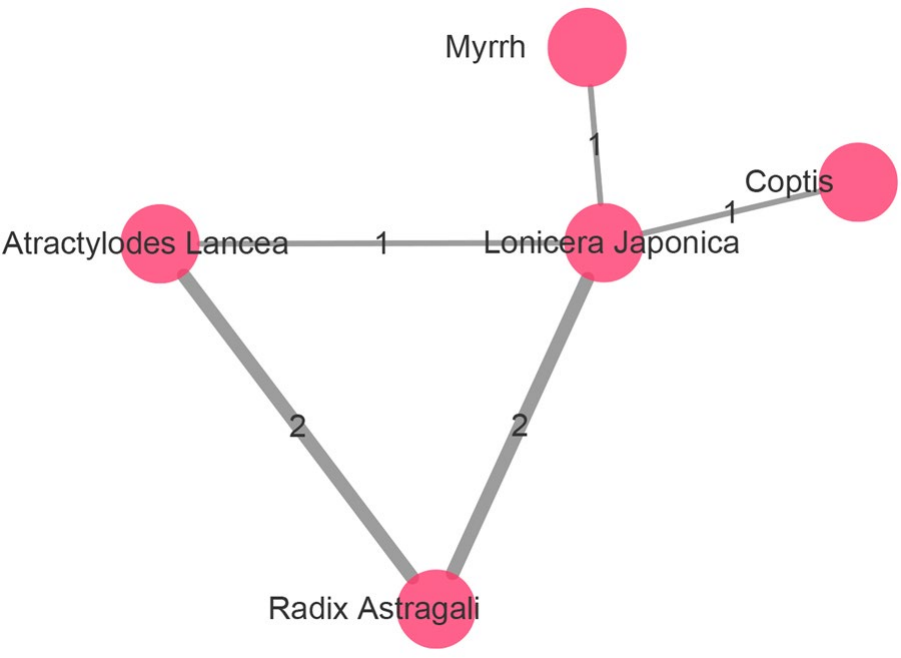
The relationship between herbs. Same ingredients shared between 5 herbs, the width of the lines represent the number of same ingredients shared between herbs.

Among the 357 ingredients, 350 of them were extracted from individual herb while 7 were shared among herbs which indicated a cumulative effect (Fig. 1). For instance, *Lonicera Japonica* and *Radix Astragali* shared 2 compounds γ-sitosterol and β-sitosterol, *Radix Astragali* and *Atractylodes Lancea* shared adeninenucleoside and uridine while chlorogenic acid was found in *Lonicera Japonica* and *Coptis*. Interestingly, most of the common ingredients can reduce inflammation through various approaches such as decreasing tumor necrosis factor (TNF) and interleukin 6 (IL-6) production, preventing leukocyte extravasation and antibacterial effect^23–26^.

**Figure 2:**
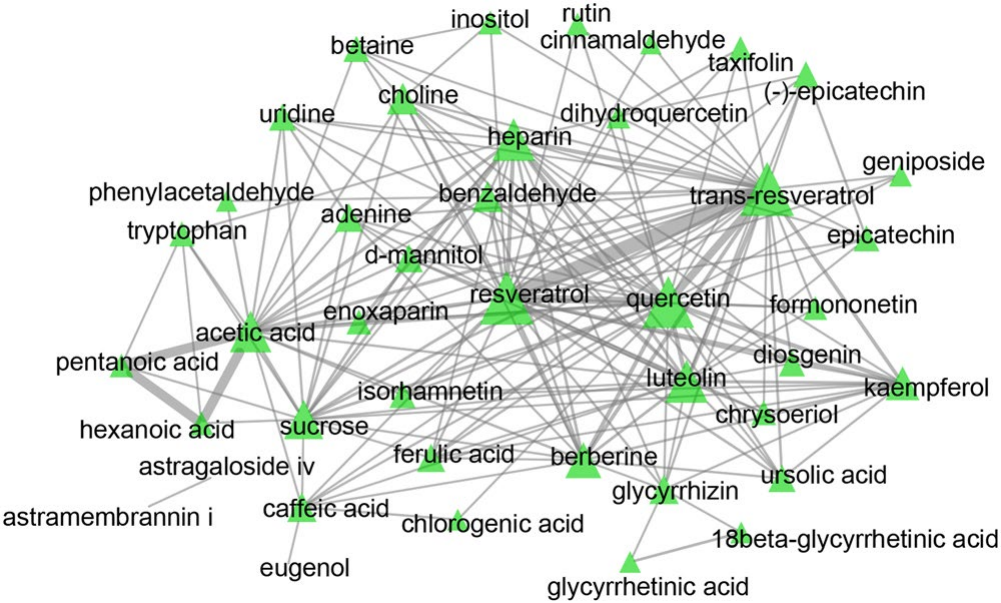
The relationship between ingredients. Sharing targets more than 3 between ingredients were showed up in the network. The width of the lines represent the number of shared targets between ingredients.

We then extracted targets that were highly correlated with the ingredients from TCMID and STITCH (Supplement Tables S5 and 6). Totally, we found that 80 ingredients had effect on 1101 proteins. 452 targets are shared among ingredients while other 649 targets are unique. Several small molecules such as resveratrol, trans-resveratrol and quercetin seem to have similar features since they possessed more than 70 identical targeting proteins (Fig. 2).

### Functional analysis of Y-Y-T targets

**Figure 3:**
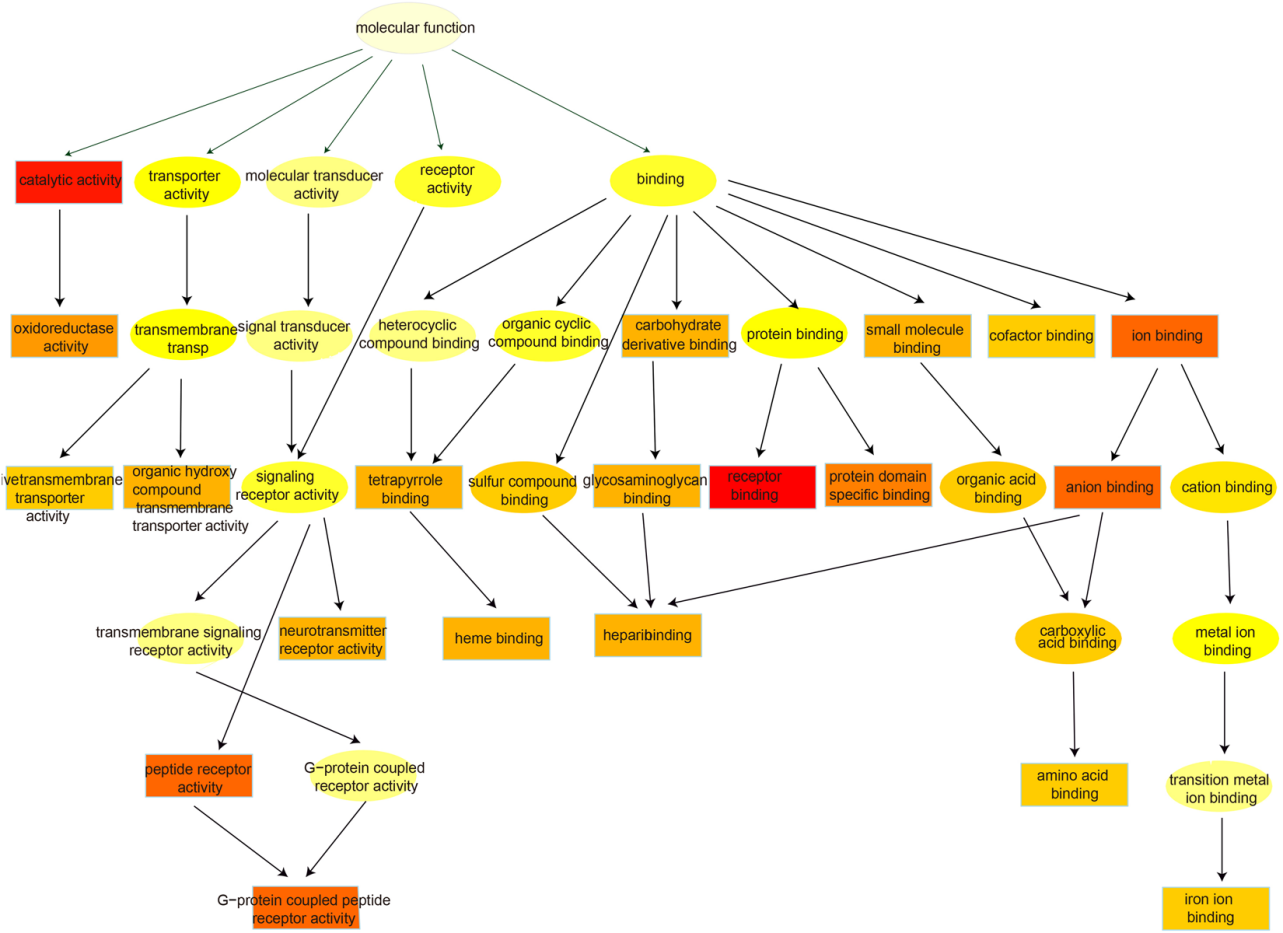
Classification of the formula’s top 20 enriched Go terms. The different colors represent different functional categories and the lower levels affiliation to upper levels.

Gene Ontology (GO) analysis and KEGG pathway analysis were utilized to analyze the main therapeutic effect of the formula. Results of GO analysis (Supplement Table S7) showed that targets of Y-Y-T were mainly related to 5 parts, namely catalytic, transporter, receptor and molecular transducer activity as well as binding (Fig. 3). Specifically, heparin binding (*p* = 1.20E-11), peptide receptor activity, G-protein coupled (*p* = 1.20E-09), neuropeptide hormone activity (*p = *3.10E-06) are closely associated with AS. For example, a neuropeptide of vasoactive intestinal polypeptide plays a significant role in neuroendocrine-immune-gastrointestinal systems^27^ and magnesium-containing intramedullary nails had the ability to repair osteoporosis^28^. The enriched terms showed us the essential function of the formula for treating AS.

80 enriched KEGG pathways (Supplement Table S8) could be divided into six categories including Human Diseases, Organismal Systems, Environmental Information Processing, Metabolism, Genetic Information Processing and Cellular Processes. In Human Diseases group, Inflammatory bowel disease (IBD) (*P* = 5.10E-03) and Rheumatoid arthritis (RA) (*P* = 1.00E-02) are significantly associated with AS. The etiopathogenesis of IBD and AS is analogous because of the similar genetic and immunologic background. Moreover, RA and AS patients shared common bone metabolism biomarkers and symptoms^5,29^. Additionally, some pathways in immune system were involved in the onset and development of AS. For example, in an inflammatory environment, the activation of AMPK (*p* = 5.00E-04) and TLR signaling pathway (*P* = 7.30E-04) would facilitate AS incidence^30^. T cell receptor signaling pathway (*P* = 9.10E-04) participated in hip joint ligament ossification^31^. Moreover, other pathways focus on endocrine, sensory and nervous systems and are related to signaling molecules interaction and transduction. In metabolism, the formula may involve in the metabolism process of amino acid, carbohydrate and lipid.

### Comparison between Y-Y-T targets, other AS drug targets, AS disease proteins and differentially expressed genes of AS patients

**Figure 4:**
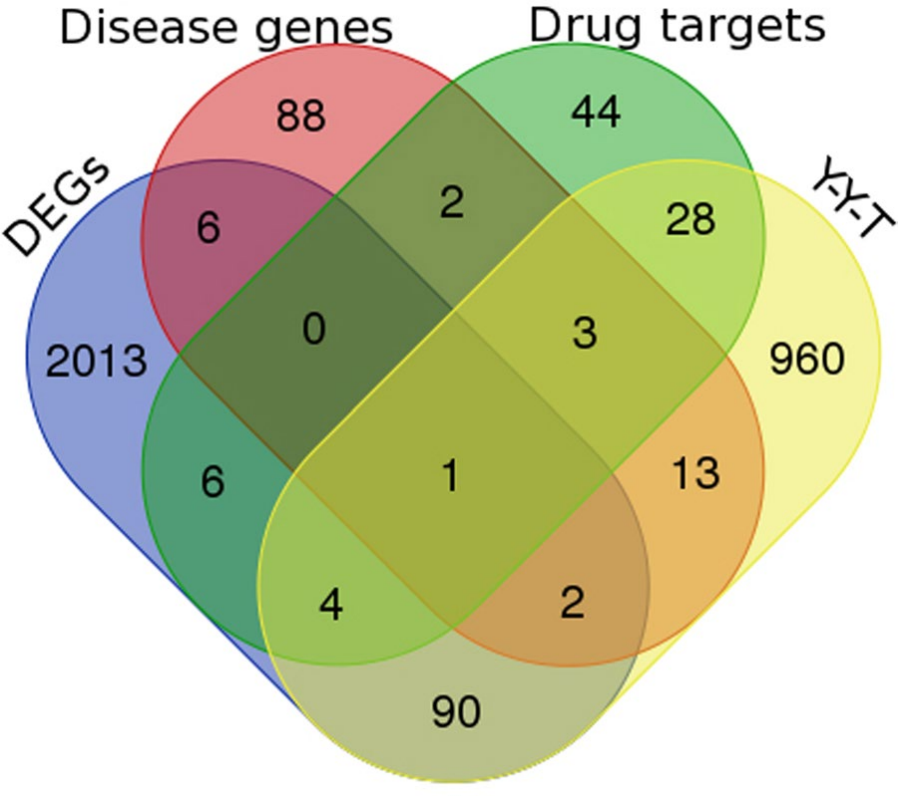
The overlaps between formula targets, disease genes, drug targets and DEGs. Blue: disease genes; Yellow: DEGs; pink: Y-Y-T targets; green: drug targets.

In order to discover potential therapeutic mechanism of Y-Y-T for AS treatment, we made comparison between four datasets. The gene expression profile GSE 73754 was downloaded from the GEO database. T-test with Benjamin-Hochberg correction for multiple comparisons was carried out to identify DEGs, resulting in 2122 DEGs (Supplement Table S9). The other 3 datasets, 1101 targets of Y-Y-T formula, 88 targets of 19 FDA approved drugs related to AS (Supplement Table S10) and 115 AS disease genes (Supplement Table S11), were retrieved from related public databases. As showed in Fig. 4. 19, 97 and 36 formula targets overlapped with disease proteins, proteins encoded by DEGs and drug targets, respectively. TNF, which is the only one target shared the 4 groups, is apparently a key protein in therapy.

### Targets association networks between proteins encoded by disease genes and formula targets

**Figure 5:**
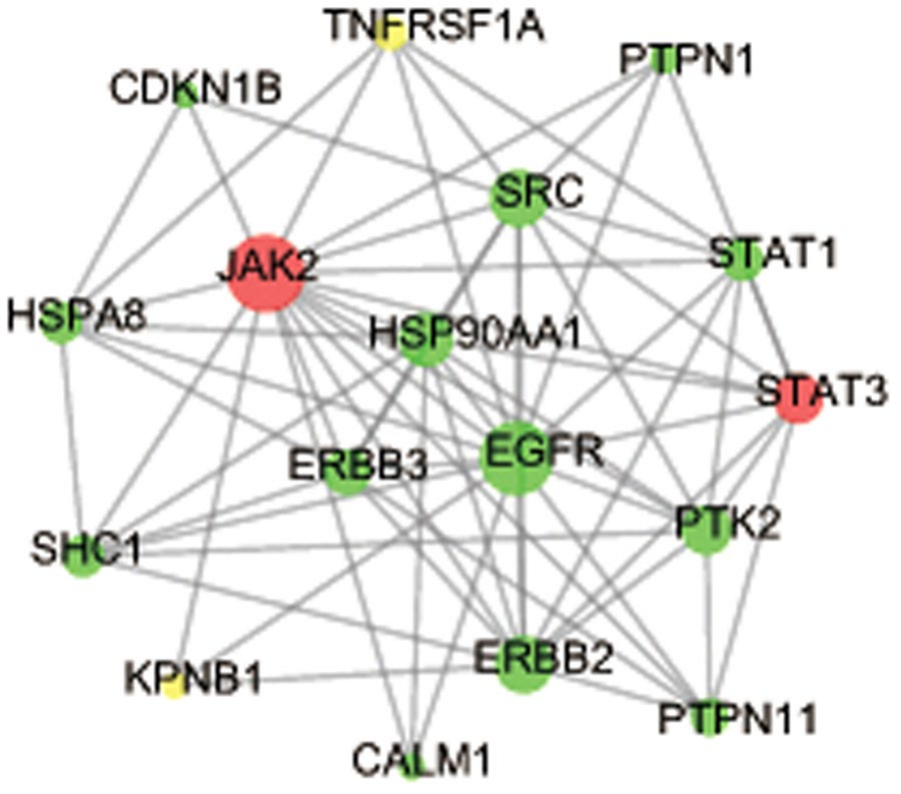
A cluster PPI network of the Y-Y-T and disease genes. Green: formula targets; yellow: disease genes; red: formula and disease genes.

To further explore the underlying mechanism of the formula, we applied PPI networks to uncover the functional relationship between formula targets and disease proteins. 811 formula targets and 77 proteins encoded by disease genes formed 3732 pairs of PPIs. As showed in Fig. 5, two proteins overlapped between the two groups (janus kinase 2 [JAK2], signal transducer and activator of transcription 3 [STAT3]) were defined as essential targets. 17 nodes (13 formula targets, 2 essential targets and 2 AS disease genes) formed a highly-connected cluster. In this cluster, JAK2 and epidermal growth factor receptor (EGFR) appear to act as hub due to their high degree of association. JAK2 and STAT3 are both regulatory factors of IL-23 pathway which is an important etiological factor for AS^32^ while EGFR has been recognized as a monoclonal antibody targets for the treatment of AS^33^ and involves in AS progression. Furthermore, JAK2, signal transducer and activator of transcription 1 (STAT1), SHC-adaptor protein (SHC), protein tyrosine kinase 2 (PTK2) jointly take part in chemokine signal pathway which has been proven to affect immune-mediated inflammatory disease. Heat shock protein 90 alpha family class A member 1 (HSP90AA1) is a formula target directly or indirectly connected with 4 disease proteins. It was suggested that HSP90AA1-targeted agents can balance the inflammation-immune system through blocking inflammation, cytokine production, protein kinase activity and angiogenic signaling^34^.

### Targets association networks between DEGs and formula targets

**Figure 6:**
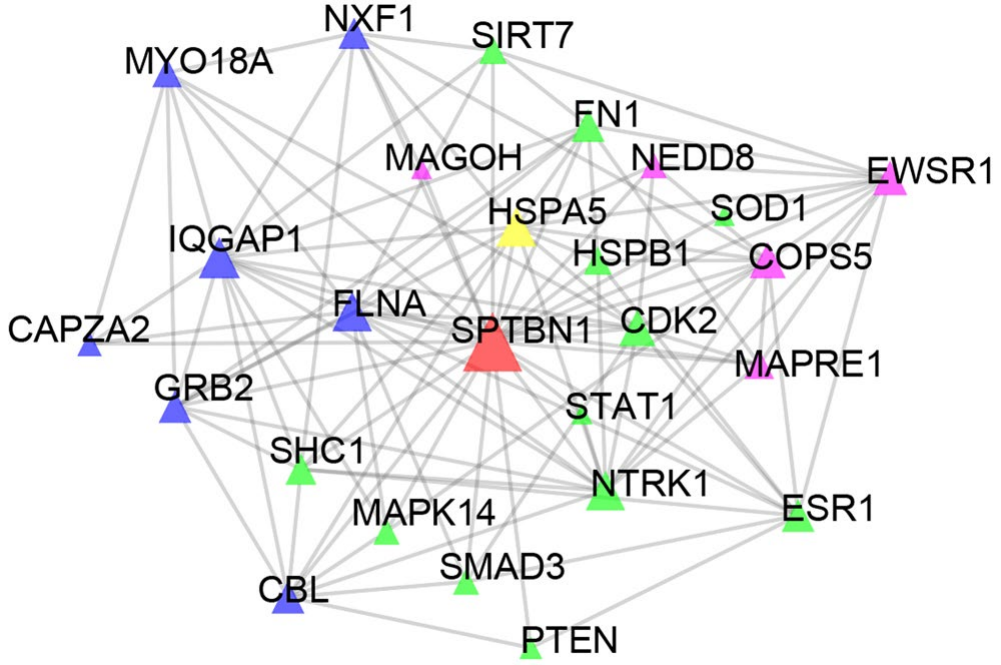
A cluster PPI network of the Y-Y-T formula and DEGs. Green: formula targets; red: formula targets and up-regulated DEGs; yellow: formula targets and down -regulated DEGs; blue: up-regulated DEGs; purple: down -regulated DEGs.

90 targets overlapped between 869 Y-Y-T targets and 1266 DEGs and 13,461 pairs of PPIs were formed by the two datasets. 79% Y-Y-T targets associated with 60% DEGs. For 90 Y-Y-T targets sharing with the DEGs, 41 of them were up-regulated DEGs while 49 were down-regulated DEGs. As showed in Fig. 6, spectrin beta, non-erythrocytic 1 (SPTBN1), shared by 2 groups, was a hub connected with other 25 nodes including estrogen receptor 1 (ESR1), neurotrophic receptor tyrosine kinase 1 (NTRK1) and phosphatase and tensin homolog (PTEN), etc. ESR1 is related to low bone mineral density (BMD) in AS^35,36^. NTRK1 is involved in the pain mechanism^35^ while PTEN could active PI3K/Akt signaling which is related to the bone metabolism of AS patients^37^. The formula may act on above mentioned proteins to intervene the progression of AS.

### Targets association networks between drug targets and formula targets

**Figure 7:**
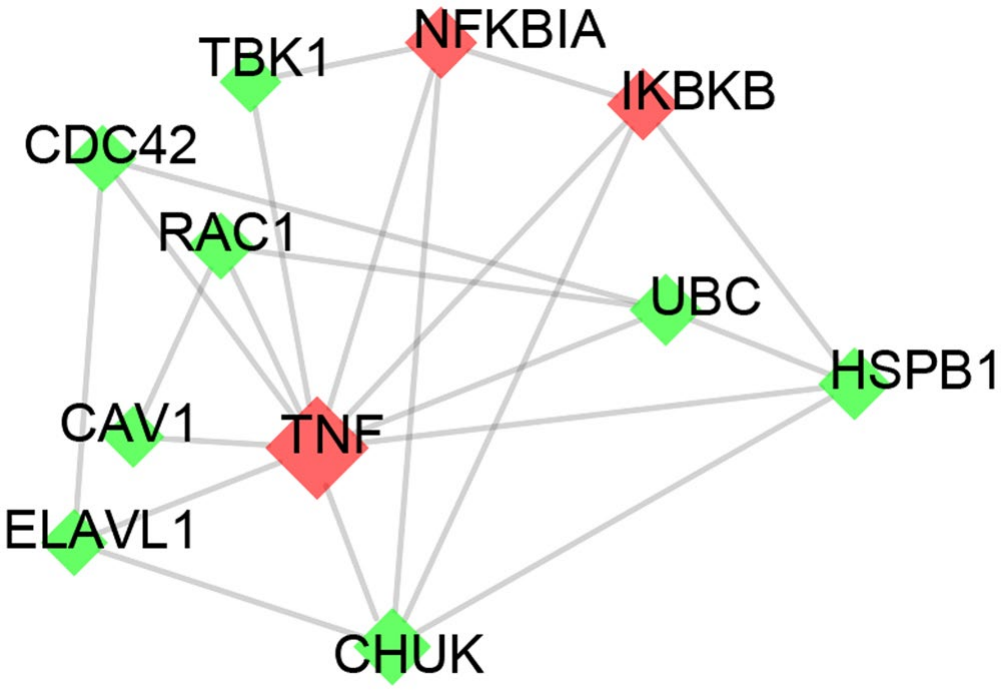
A cluster PPI network of the Y-Y-T and drug targets. Green: formula targets; red: formula targets and drug targets. Y-Y-T; Yun-Pi-Yi-Shen-Tong-Du-Tang.

810 Y-Y-T targets interact with 68 drug targets and there are 34 proteins overlapped between the two dataset, including TNF and prostaglandin G/H synthase 2 (PTGS2) in Etanercept, NFKB inhibitor alpha (NFKBIA) and inhibitor of nuclear factor kappa B kinase subunit beta (IKBKB) in Acetylsalicylic acid. These drugs are routine medication in the course of AS treatment, indicating a similar mechanism in formula. As presented in Fig. 7, hub target TNF was connected with other 10 targets. TNF is a cytokine involved in systemic inflammation and anti-TNF drugs are widely utilized for rheumatism intervention in clinical^38^. Another drug target NFKBIA and its’ promoter polymorphisms are associated with the development of AS^39^. In addition, formula target conserved helix-loop-helix ubiquitous kinase (CHUK) has been reported to effect as anti-TNF^40^ and help control the inflammation. All these results expounded that a combination of AS drug targets and Y-Y-T targets can enhance the curative effect.

## Discussion

Y-Y-T is a formula created by Prof. Chengping Wen. Clinical evidence demonstrated that Y-Y-T is beneficial for AS patients in relieving clinical symptoms and enhancing physical quality. In this study, we further deciphered the potential underlying mechanism of this formula.

BASDAI is a self-administer instrument consisting of six horizontal visual analog scales which is used to estimate the disease progression, measure severity of fatigue, spinal, peripheral joint pain and localized tenderness and morning stiffness^19^. ESR and CRP as inflammation biomarkers have been widely utilized for evaluating disease activity and medicine efficacy^41^, while ASDAS-CRP and ASDAS-ESR are spondylitis-specific assessments^42^. The retrospective research results indicated that Y-Y-T can reduce the symptoms of morning stiffness, fatigue, pain and decrease disease activity, proving the effectiveness.

In this study, herbs, compounds and targets of Y-Y-T were jointly exploited in layers of in-depth analysis, which gave us a better understanding of the potential therapeutic mechanisms of Y-Y-T. We found that there are a few common compounds among the 11 herbs. Some compounds like resveratrol, trans-resveratrol, quercetin targeting more than 70 proteins seems to be critical compounds in the therapy. For example, resveratrol can restrain growth of klebsiella pneumonia^43^ which is an inflammation trigger of AS, while quercetin has anti-inflammatory and analgesic effects^44^. Meanwhile, some targets were shared among the compounds, such as IL-6, TNF and PTGS2, indicating an accumulative effect. IL-6, TNF, PTGS2 are classic pro-inflammatory cytokines secreted by a variety of immune cells and are either associated with disease activity or some inflammatory markers^45,46^. However, berberine and astibin could inhibit the expression and secretion of IL-6, TNF and PTGS2^47–49^.

Functional analysis illustrated that Y-Y-T was significantly enriched on GO terms of molecular transducer activity, receptor activity as well as binding, and pathways related to immune, inflammatory and metabolism, like metabolic pathways, TLR signaling pathway, AMPK signaling pathway, T cell receptor signaling pathway, etc. Most of these pathways are involved in the development of AS while others such as TNF signaling pathway is related with the onset of AS, complement and coagulation cascades, with 36 formula targets engaged, is related to immunoprotecive and regulatory function^50^.

We found that 13 drug targets were shared among more than 5 compounds. Among them, TNF, PTGS2, CYP1A1 (cytochrome P450 family 1 subfamily A member 1) seems to be hub targets of high frequency occurrence in herbs which produced a superposition effect. PPI networks between drug targets and formula targets illustrated the underlying mechanism that how Y-Y-T enhanced the efficacy of western medicine. Furthermore, PPI networks were constructed between four datasets and hub proteins were selected in each network. The four datasets formed a complex network, indicating a close relationship between formula targets and AS suggested that proteins such as JAK2, STAT3, HSP90AA1, TNF and PTEN are the key targets in PPI networks.

In all, the integrative investigation of Y-Y-T targets gave us a better understanding how this formula works on AS. Our systemic method which is different from traditional research revealed the therapeutic mechanism of Y-Y-T on AS and provides us an alternative way to investigate the TCM formulae.

## Materials and Methods

### Clinical data collection

This retrospective study was carried out under the approval of Ethics committee of Zhejiang Chinese Medical University and the methods were carried out in accordance with the approved guidelines. Written informed consents were obtained from all patients before they participated in the study. We stuck to the basic principle of “freely given informed consent”. Diagnostic criteria refer to the 1984 revised New York AS diagnostic criteria. Patients’ detailed information was collected either from outpatient medical records in The Second Affiliated Hospital of Zhejiang University of TCM or from the patients’ follow-up visits. Those who had other diseases or suffering from liver and renal dysfunction were removed. Patients with poor effect to the western medicine and continuously suffering from morning stiffness, persistent lower-back and multiple joints pain participated Y-Y-T add-on treatment for 6 months. Finally, 30 cases finished the observation. General information, serum markers (ESR and CRP) as well as indicators of disease progression were recorded. Student *t* test was applied to evaluate the otherness of indexes in different treating period.

### Collection of Y-Y-T data, AS disease proteins and AS drug targets

Ingredients of the 11 herbs in Y-Y-T and targets of those ingredients were gleaned from Traditional Chinese Medicine Integrated Database (TCMID, http://www.megabionet.org/tcmid/)^51^. Online Mendelian Inheritance in Man (OMIM, http://omim.org)^52^ and The Genetic Association Database (GAD, http://geneticassociationdb.nih.gov)^53^ were applied for the collection of AS disease proteins while AS drug targets were gathered from Drug Bank (http://www.drugbank.ca)^54^.

### Microarray data processing of AS samples

The profile of GSE 73754 was downloaded from Gene Expression Omnibus database^55^ (GEO, http://www.ncbi.nlm.nih.gov/geo/). The dataset consists of 42 AS patient samples and 30 normal samples. Expression values were normalized by rma function in R. Genes with adjusted p-value less than 0.05 were defined as DEGs.

### GO enrichment analysis

GO and KEGG pathway enrichment analysis for Y-Y-T targets were undertaken through the online analytical tools DAVID Bioinformatics Resources 6.7 (http://david.abcc.ncifcrf.gov/) and topGO function in R.

### Target association network

Homo sapiens protein-protein interaction data was extracted from InWeb_InBioMap, the most complete Human PPI database^56^. The InWeb_InBioMap database is a newly released comprehensive database which provided a scored human protein-protein interaction network with more than 500,000 interactions and the data of PPI was derived from various protein interaction resources based on experiment with highly accurate. PPIs were then verified and benchmarked, high degree targets were selected for further analysis. Networks were visualized by Cytoscape 3.4.0.

## Electronic supplementary material

Tables
