# Supplementary material for: Dissecting the Underlying Pharmaceutical Mechanism of Chinese Traditional Medicine Yun-Pi-Yi-Shen-Tong-Du-Tang Acting on Ankylosing Spondylitis through Systems Biology Approaches
Source: Sci Rep. 2017 Oct 18;7:13436. doi: 10.1038/s41598-017-13723-3 (PMC5647417; doi:10.1038/s41598-017-13723-3)
Supplement: Supplementary file 1 — Tables [file 41598_2017_13723_MOESM1_ESM.doc]

Dissecting the Underlying Pharmaceutical Mechanism of Chinese Traditional Medicine Yun-Pi-Yi-Shen-Tong-Du-Tang Acting on Ankylosing Spondylitis through Systems Biology Approaches

Duoli Xie^1, #^, Lin Huang^1, #^, Guanghui Zhao^2^, Yiran Yu^1^, Jiawei Gao^1^, Haichang Li^1^, Chengping Wen^1, *^

^1^ TCM Clinical Basis Institute, Zhejiang Chinese Medicine University, 548 Binwen Road, Hangzhou, Zhejiang, 310000, China

^2^ Guangzhou University of Chinese Medicine, Mathematical Engineering Academy of Chinese Medicine, Guangzhou, 510006, China

^#^ These authors contributed equally to this work.

* Correspondence should be addressed to C.W. E-mail: ([wengcp@163.com](mailto:wengcp@163.com); Tel: +86-571-86613644.)

**Supplement Table S1** Basic information of 30 patients.

| **Patient** | **SEX** | **AGE(y)** | **HIGH(cm)** | **WEIGHT(kg)** | **COURSE(y)** | **Finger-ground distance(cm)** | **Occipital-wall distance** | **Chest expansion(cm)** | **HLA-B27** |
| --- | --- | --- | --- | --- | --- | --- | --- | --- | --- |
| P1 | F | 34 | 155 | 55 | 3 | 6 | 0 | 2.5 | + |
| P2 | M | 16 | 180 | 63 | 2.5 | 0 | 4 | 7 | + |
| P3 | M | 31 | 175 | 75 | 8 | 20 | 0 | 0 | 0 |
| P4 | F | 26 | 160 | 57 | 11 | 0 | 0 | 3 | + |
| P5 | F | 50 | 155 | 58 | 18 | 12 | 4 | 1.5 | + |
| P6 | F | 34 | 166 | 55 | 13 | 0 | 0 | 1.5 | + |
| P7 | F | 36 | 160 | 50 | 13 | 0 | 0 | 2 | + |
| P8 | F | 36 | 160 | 57 | 15 | 17 | 0 | 1.5 | + |
| P9 | F | 23 | 160 | 55 | 2.5 | 0 | 0 | 3 | + |
| P10 | M | 39 | 172 | 56 | 14 | 25 | 0 | 2 | + |
| P11 | M | 35 | 165 | 55 | 11 | 15 | 0 | 2.5 | + |
| P12 | M | 19 | 168 | 58 | 3 | 6 | 1 | 5 | + |
| P13 | M | 48 | 165 | 57 | 10 | 15 | 25 | 1 | 0 |
| P14 | F | 41 | 167 | 57 | 12 | 0 | 0 | 3 | + |
| P15 | M | 54 | 178 | 82 | 0.83 | 0 | 0 | 4.5 | + |
| P16 | F | 35 | 156 | 48 | 7.5 | 0 | 0 | 2.5 | + |
| P17 | M | 58 | 176 | 75 | 10 | 30 | 3 | 1 | - |
| p18 | F | 35 | 161 | 56 | 5 | 0 | 0 | 2.5 | + |
| P19 | M | 21 | 178 | 65 | 1 | 10 | 5 | 7 | + |
| P20 | M | 40 | 175 | 79 | 0.5 | 0 | 0 | 5 | + |
| P21 | M | 42 | 177 | 89 | 10 | 24 | 20 | 0.5 | 0 |
| P22 | M | 38 | 171 | 60 | 9 | 20 | 1 | 5 | + |
| P23 | M | 48 | 175 | 70 | 8 | 15 | 2 | 3 | + |
| p24 | F | 44 | 160 | 53 | 2 | 0 | 0 | 2 | + |
| P25 | F | 27 | 165 | 57 | 4 | 0 | 0 | 5 | + |
| P26 | F | 32 | 160 | 54 | 0.5 | 0 | 0 | 7 | + |
| p27 | M | 48 | 175 | 72 | 5 | 48 | 4.5 | 2 | + |
| P28 | F | 66 | 161 | 50 | 9 | 20 | 0 | 4 | + |
| p29 | M | 17 | 180 | 72 | 4 | 27 | 9 | 2 | + |
| p30 | M | 46 | 178 | 63 | 0.41 | 20 | 0 | 0.5 | + |

**Supplement Table S2** BASDAI questions score and patient global score of 30 AS patients.

*Q1—‘How would you describe the overall level of fatigue/tiredness you have experienced?’; Q2—‘How would you describe the overall level of neck, back or hip pain you have had?’; Q3—‘How would you describe the overall level of pain/swelling in joints other than neck, back or hips you have had?’; Q4—‘How would you describe the overall level of discomfort you have had from any areas tender to touch or pressure?’; Q5—‘How would you describe the overall level of morning stiffness you have had from the time you wake up?’; Q6—‘How long does your morning stiffness last from the time you wake up?’. All the questions assessed on a visual analogue scale (from 0 to 10).

| **Patient** | **Time** | **Q 1** | **Q 2** | **Q 3** | **Q 4** | **Q 5** | **Q 6** | **Patient global** |
| --- | --- | --- | --- | --- | --- | --- | --- | --- |
| P1 | T1 | 8 | 8 | 7 | 8 | 9 | 3 | 8 |
|  | T2 | 2 | 2 | 0 | 2 | 0 | 0 | 2 |
| P2 | T1 | 1 | 8 | 0 | 0 | 7 | 0 | 5 |
|  | T2 | 3 | 2 | 0 | 3 | 2 | 0 | 2 |
| P3 | T1 | 0 | 0 | 5 | 5 | 3 | 2 | 5 |
|  | T2 | 5 | 1 | 0 | 0 | 0 | 0 | 2 |
| P4 | T1 | 5 | 9 | 6 | 0 | 6 | 4 | 8 |
|  | T2 | 5 | 7 | 0 | 0 | 4 | 2 | 6 |
| P5 | T1 | 7 | 9 | 9 | 8 | 8 | 10 | 8 |
|  | T2 | 2 | 2 | 4 | 0 | 0 | 0 | 3 |
| P6 | T1 | 10 | 0 | 0 | 10 | 10 | 10 | 8 |
|  | T2 | 0 | 1 | 1 | 1 | 0 | 0 | 0 |
| P7 | T1 | 0 | 1 | 0 | 0 | 1 | 0 | 0 |
|  | T2 | 0 | 0 | 0 | 0 | 0 | 0 | 0 |
| P8 | T1 | 0 | 10 | 0 | 0 | 10 | 6 | 8 |
|  | T2 | 0 | 5 | 0 | 0 | 5 | 5 | 5 |
| P9 | T1 | 0 | 10 | 10 | 0 | 6 | 0 | 10 |
|  | T2 | 0 | 2 | 2 | 0 | 0 | 0 | 2 |
| P10 | T1 | 4 | 8 | 7 | 6 | 6 | 5 | 7 |
|  | T2 | 2 | 2 | 3 | 5 | 1 | 0 | 2 |
| P11 | T1 | 2 | 8 | 2 | 7 | 4 | 2 | 7 |
|  | T2 | 1 | 0 | 0 | 1 | 2 | 0 | 4 |
| P12 | T1 | 0 | 10 | 10 | 10 | 10 | 3 | 10 |
|  | T2 | 0 | 4 | 4 | 3 | 3 | 1 | 4 |
| P13 | T1 | 10 | 8 | 8 | 5 | 6 | 3 | 7 |
|  | T2 | 5 | 4 | 4 | 5 | 3 | 3 | 6 |
| P14 | T1 | 4 | 6 | 5 | 5 | 5 | 3 | 8 |
|  | T2 | 5 | 6 | 0 | 0 | 0 | 0 | 5 |
| P15 | T1 | 5 | 6 | 0 | 0 | 7 | 7 | 8 |
|  | T2 | 1 | 2 | 0 | 0 | 0 | 0 | 2 |
| P16 | T1 | 5 | 7 | 6 | 8 | 5 | 7 | 8 |
|  | T2 | 3 | 5 | 3 | 5 | 2 | 2 | 3 |
| P17 | T1 | 5 | 0 | 5 | 0 | 5 | 3 | 6 |
|  | T2 | 3 | 0 | 2 | 0 | 3 | 0 | 3 |
| P18 | T1 | 5 | 5 | 5 | 2 | 5 | 10 | 5 |
|  | T2 | 2 | 2 | 2 | 1 | 2 | 3 | 1 |
| P19 | T1 | 4 | 4 | 5 | 5 | 0 | 0 | 3 |
|  | T2 | 5 | 4 | 4 | 2 | 0 | 0 | 2 |
| P20 | T1 | 8 | 5 | 0 | 3 | 6 | 0 | 5 |
|  | T2 | 6 | 1 | 0 | 1 | 1 | 0 | 2 |
| P21 | T1 | 10 | 6 | 5 | 0 | 10 | 0 | 9 |
|  | T2 | 7 | 6 | 5 | 0 | 3 | 0 | 4 |
| P22 | T1 | 8 | 9 | 0 | 0 | 3 | 0 | 2 |
|  | T2 | 5 | 9 | 2 | 0 | 0 | 0 | 2 |
| P23 | T1 | 3 | 7 | 8 | 8 | 9 | 8 | 8 |
|  | T2 | 3 | 6 | 6 | 5 | 6 | 6 | 6 |
| P24 | T1 | 2 | 8 | 0 | 0 | 0 | 0 | 7 |
|  | T2 | 1 | 1 | 0 | 0 | 0 | 0 | 1 |
| P25 | T1 | 8 | 8 | 8 | 8 | 8 | 0 | 8 |
|  | T2 | 3 | 0 | 4 | 3 | 1 | 0 | 1 |
| P26 | T1 | 3 | 4 | 3 | 3 | 4 | 3 | 3 |
|  | T2 | 3 | 2 | 2 | 2 | 2 | 2 | 2 |
| P27 | T1 | 6 | 10 | 0 | 10 | 10 | 10 | 10 |
|  | T2 | 6 | 5 | 0 | 5 | 10 | 10 | 5 |
| P28 | T1 | 10 | 10 | 2 | 0 | 10 | 7 | 9 |
|  | T2 | 5 | 6 | 1 | 0 | 8 | 5 | 6 |
| P29 | T1 | 0 | 0 | 6 | 3 | 1 | 2 | 4 |
|  | T2 | 0 | 0 | 0 | 0 | 1 | 2 | 0 |
| P30 | T1 | 5 | 5 | 5 | 5 | 5 | 5 | 5 |
|  | T2 | 3 | 3 | 3 | 5 | 3 | 3 | 3 |
|  |  |  |  |  |  |  |  |  |

**Supplement Table S3** The clinical parameters of 30 patients.

| **Patient** | **T1-CRP(mg/L)** | **T2-CRP(mg/L)** | **T1-ESR(mm/h)** | **T2-ESR(mm/h)** | **BASDAI-Score (before)** | **BASDAI-Score （later）** | **ASDAS-CRP（before）** | **ASDAS-CRP（later）** | **ASDAS-ESR（before）** | **ASDAS-ESR（later）** |
| --- | --- | --- | --- | --- | --- | --- | --- | --- | --- | --- |
| P1 | 3.4 | 1.46 | 17 | 6 | 7.4 | 1.2 | 3.390849 | 0.9831934 | 3.55307 | 1.1017 |
| P2 | 4.67 | 4.62 | 14 | 20 | 2.5 | 1.8 | 2.522674 | 1.461546 | 2.293306 | 1.694336 |
| P3 | 11.2 | 16.1 | 7 | 6 | 2.5 | 1.2 | 2.479331 | 1.9848264 | 1.908205 | 1.0227 |
| P4 | 0.15 | 6 | 10 | 33 | 5 | 3 | 2.719922 | 2.749682 | 3.333547 | 3.052157 |
| P5 | 24 | 25 | 40 | 55 | 8.4 | 1.6 | 5.069729 | 2.7504379 | 4.932095 | 3.013946 |
| P6 | 24.29 | 9.31 | 4 | 17 | 6 | 0.6 | 3.330407 | 1.5448732 | 2.18 | 1.37307 |
| P7 | 0.32 | 0.2 | 28 | 10 | 0.3 | 0 | 0.281749 | 0.1055642 | 1.62941 | 0.926547 |
| P8 | 13.71 | 2.65 | 31 | 16 | 3.6 | 2 | 3.994657 | 2.194647 | 3.739355 | 2.477 |
| P9 | 3 | 3.81 | 14 | 24 | 4.6 | 0.8 | 3.842664 | 1.5174336 | 3.876306 | 1.991401 |
| P10 | 24.34 | 18.32 | 32 | 33 | 6.1 | 2.5 | 4.41055 | 2.3955005 | 4.027458 | 2.325157 |
| P11 | 31.3 | 21.77 | 26 | 32 | 4.4 | 0.6 | 4.012064 | 2.249632 | 3.227013 | 2.109458 |
| P12 | 31.6 | 20 | 41 | 34 | 7.3 | 2.6 | 5.231417 | 3.0367785 | 4.863115 | 2.889469 |
| P13 | 12.06 | 16.7 | 13 | 20 | 7.1 | 4.2 | 3.983772 | 3.2737939 | 3.374427 | 2.855336 |
| P14 | 1 | <5 | 36 | 17 | 4.8 | 2.2 | 2.546332 | - | 3.773 | 2.24707 |
| P15 | 14.6 | 10.6 | 23 | - | 3.6 | 0.6 | 3.60267 | 1.881132 | 3.266179 | - |
| P16 | 0.86 | 2.5 | 10 | 13 | 6.4 | 3.6 | 2.930314 | 1.9953498 | 3.382547 | 2.186427 |
| P17 | <1.0 | <1.0 | 4 | 4 | 2.8 | 1.3 | - | - | 1.901 | 1.097 |
| P18 | <1.0 | <1.0 | 8 | 12 | 2.8 | 1.3 | - | - | 2.908729 | 1.664982 |
| P19 | 9 | 5 | 7 | 2 | 3.6 | 3 | 2.512197 | 2.0334287 | 1.860205 | 1.300365 |
| P20 | - | 2.27 | 5 | 1 | 3.8 | 1.7 | - | 1.0269934 | 1.615168 | 0.598 |
| P21 | 17.6 | 18.74 | 5 | 5 | 5.2 | 3.9 | 3.773511 | 3.2579526 | 2.576168 | 2.011168 |
| P22 | 12.8 | 6.53 | - | 14 | 3.7 | 3.2 | 2.828683 | 2.6239402 | - | 2.205306 |
| P23 | 24.42 | 30.96 | 86 | 39 | 6.9 | 5.2 | 4.648375 | 4.1779369 | 5.41417 | 3.911784 |
| P24 | <0.5 | <8.0 | 7 | 4 | 2 | 0.4 | - | - | 2.198205 | 0.778 |
| P25 | 3.3 | 1.7 | 26 | 32 | 7.2 | 2.1 | 3.276538 | 0.9770928 | 3.718013 | 2.114458 |
| P26 | 0.3 | <1.0 | 17 | - | 3.3 | 2.2 | 1.358909 | - | 2.32807 | 0.694 |
| P27 | 46.67 | 14 | 35 | 15 | 7.2 | 5.2 | 5.127431 | 3.3029611 | 4.343411 | 2.784784 |
| P28 | <8.0 | 1.4 | 63 | 35 | 6.1 | 3.7 | - | 1.5958964 | 4.787615 | 2.638411 |
| P29 | 14.7 | 3.3 | 18 | 9 | 2.1 | 0.3 | 2.58837 | 0.9605381 | 2.349094 | 1.017 |
| P30 | 14.32 | 12 | 22 | 1 | 5 | 3.4 | 3.390183 | 2.5711057 | 3.109292 | 1.334 |

**Supplement Table S4** The detailed information of herbs and compounds in Y-Y-T.

357 unique compounds of 11 herbs were download from TCMID .

| **herb** | **compound** |
| --- | --- |
| Dioscoreae Nipponicae Rhizoma | dioscin |
| Dioscoreae Nipponicae Rhizoma | trillin |
| Dioscoreae Nipponicae Rhizoma | 25-d-spirosta-3,5-diene |
| Dioscoreae Nipponicae Rhizoma | allantoin |
| Dioscoreae Nipponicae Rhizoma | diosgenin |
| Atractylodes Lancea | 10-epiatractyloside a |
| Atractylodes Lancea | beta-eudesmol |
| Atractylodes Lancea | scopoletin β-d-xylopyranosyl-(1→6)-β-d-glucopyranoside |
| Atractylodes Lancea | bata-caryophyllene |
| Atractylodes Lancea | uridine |
| Atractylodes Lancea | elemol |
| Atractylodes Lancea | elemicin |
| Atractylodes Lancea | elenolide |
| Atractylodes Lancea | β-elemene |
| Atractylodes Lancea | 2-(8-methyl-2,8-dihydroxy-9-oxo-2-hydroxymethylbicyclo[5.3.0]decan-7-yl)isopropanol glucoside |
| Atractylodes Lancea | diterbutyl phthalate |
| Atractylodes Lancea | 2-methyl-4-(1,1-dimethylethyl) phenol |
| Atractylodes Lancea | 2-furaldehyde |
| Atractylodes Lancea | 2-(8-methyl-2,8,9-trihydroxy-2-hydroxymethyl-bicyclo[5.3.0]decan-7-yl)isopropanolglucoside |
| Atractylodes Lancea | cis-atractyloside i |
| Atractylodes Lancea | atractylodin |
| Atractylodes Lancea | (2r,3r,5r,7r,10s)-atractyloside g 2-o-β-d-glucopyranoside |
| Atractylodes Lancea | atractyloside i |
| Atractylodes Lancea | atractylenolide i |
| Atractylodes Lancea | atractylenolide ii |
| Atractylodes Lancea | (1s,4s,5s,7r,10s)-10,11,14-trihydroxyguai-3-one 11-o-β-d-glucopyranoside |
| Atractylodes Lancea | (+)-eudesma-4(15),7(11)-dien-8-one |
| Atractylodes Lancea | eudesobovatol a |
| Atractylodes Lancea | (1s,4s,5r,7r,10r)-11,14-dihydroxyguai-3-one11-o-β-d-glucopyranoside |
| Atractylodes Lancea | β-eudesmol |
| Atractylodes Lancea | α-eudesmol |
| Atractylodes Lancea | gamma-selinene |
| Atractylodes Lancea | icariside f2 |
| Atractylodes Lancea | (1s,5r,7r,10r)-secoatractylolactone11-o-β-d-glucopyranoside |
| Atractylodes Lancea | (2 e)-2-decene-4,6-diyne-1,8-diol 8-o-β-d-apio-furanosyl-(1→6)-β-d-glucopyranoside |
| Atractylodes Lancea | hinesol |
| Atractylodes Lancea | alpha-chamigrene |
| Atractylodes Lancea | adeninenucleoside |
| Atractylodes Lancea | atractylon |
| Atractylodes Lancea | 3beta-acetoxy-atractylon |
| Atractylodes Lancea | beta-humulene |
| Atractylodes Lancea | 2-furancarboxylic acid |
| Atractylodes Lancea | eudesmol |
| Atractylodes Lancea | tr-saponin a |
| Atractylodes Lancea | tryptophan |
| Atractylodes Lancea | 3β-hydroxyatractylone |
| Atractylodes Lancea | 3β-acetoxy-atractylon |
| Atractylodes Lancea | syringin |
| Atractylodes Lancea | guaiol |
| Atractylodes Lancea | δ-guaiene |
| Atractylodes Lancea | α-guaiene |
| Atractylodes Lancea | α-humulene |
| Atractylodes Lancea | β-chamigrene |
| Atractylodes Lancea | (2 e,8 e)-2,8-decadiene-4,6-diyne-1,10-diol 1-o-β-d-glucopyranoside |
| Atractylodes Lancea | atractyloside g |
| Atractylodes Lancea | atractyloside e |
| Atractylodes Lancea | atractyloside d |
| Atractylodes Lancea | atractyloside c |
| Atractylodes Lancea | atractyloside b |
| Atractylodes Lancea | atractyloside a 14-o-β-d-fructofuranoside |
| Atractylodes Lancea | atractyloside a |
| Atractylodes Lancea | atractylone |
| Atractylodes Lancea | 1-3alpha,6beta-ditigloyloxytropane |
| Atractylodes Lancea | β-maaliene |
| Atractylodes Lancea | 2,6-ditertbutyl-4methyl phenol |
| Atractylodes Lancea | β-caryophyllene |
| Atractylodes Lancea | methyl 3,4,5-trimethoxycinnamate |
| Atractylodes Lancea | 1beta-hydroxybaccatin i |
| Atractylodes Lancea | β-selinene |
| Atractylodes Lancea | (1s,4s,5s,7r,10r)-10,11,14-trihydroxyguai-3-one 11-o-β-d-glucopyranoside |
| Atractylodes Lancea | 6,6'-dimethoxygossypol |
| Atractylodes Lancea | (5r,7r,10s)-isopterocarpolon β-d-gluco-pyranoside |
| Atractylodes Lancea | (+)-maalioxide |
| Rhizoma Smilacis Glabrae | dihydrokaempferol-3-o-α-l-rhamnopyranoside |
| Rhizoma Smilacis Glabrae | (-)-epicatechin |
| Rhizoma Smilacis Glabrae | 3-o-caffeoylshikimic acid |
| Rhizoma Smilacis Glabrae | 5-o-caffeoylshikimicacid |
| Rhizoma Smilacis Glabrae | 2,4,6-trihydroxyacetophenone-2,4-di-o-β-d-glucopyranoside |
| Rhizoma Smilacis Glabrae | smitilbin |
| Rhizoma Smilacis Glabrae | astilbin |
| Rhizoma Smilacis Glabrae | isobaimuxinol |
| Rhizoma Smilacis Glabrae | epicatechin |
| Rhizoma Smilacis Glabrae | daucosterol |
| Rhizoma Smilacis Glabrae | 7,6'-dihydroxy-3'-methoxyisoflavone |
| Rhizoma Smilacis Glabrae | 4,7-dihydroxy-5-methoxyl-6-methyl-8-formyl-flavan |
| Rhizoma Smilacis Glabrae | isoengelitin |
| Rhizoma Smilacis Glabrae | tufulingoside |
| Rhizoma Smilacis Glabrae | tulipalin |
| Rhizoma Smilacis Glabrae | syrionylglycerol-beta-syringaresinol |
| Rhizoma Smilacis Glabrae | trans-resveratrol |
| Rhizoma Smilacis Glabrae | isoeruboside b |
| Rhizoma Smilacis Glabrae | taxifolin |
| Rhizoma Smilacis Glabrae | enhydrin |
| Rhizoma Smilacis Glabrae | smiglaside d |
| Rhizoma Smilacis Glabrae | smiglaside c |
| Rhizoma Smilacis Glabrae | smiglaside b |
| Rhizoma Smilacis Glabrae | smiglaside a |
| Rhizoma Smilacis Glabrae | syringicacid |
| Rhizoma Smilacis Glabrae | isoengeletin |
| Rhizoma Smilacis Glabrae | smilagenin |
| Rhizoma Smilacis Glabrae | smiglaside e |
| Rhizoma Smilacis Glabrae | isoastilbin b |
| Rhizoma Smilacis Glabrae | sodium tauropythocholate |
| Rhizoma Smilacis Glabrae | resveratrol |
| Rhizoma Smilacis Glabrae | dihydroquercetin |
| Rhizoma Smilacis Glabrae | dihydroresveratrol |
| Lonicera Japonica | 1-hexanol |
| Lonicera Japonica | loniceracetalides b |
| Lonicera Japonica | isochlorogenic acid |
| Lonicera Japonica | new triterpennoid glycoside |
| Lonicera Japonica | benzyl cyanide |
| Lonicera Japonica | 2-furaldehyde |
| Lonicera Japonica | ioniceroside c |
| Lonicera Japonica | chlorogenin |
| Lonicera Japonica | chlorogenicacid |
| Lonicera Japonica | farnesal |
| Lonicera Japonica | gamma-sitosterol |
| Lonicera Japonica | benzyl alcohol |
| Lonicera Japonica | alpha-terpineol |
| Lonicera Japonica | loganoside |
| Lonicera Japonica | citronellol |
| Lonicera Japonica | citronellyl acetate |
| Lonicera Japonica | eugenol methyl ether |
| Lonicera Japonica | l-phenylalaninosecologanin |
| Lonicera Japonica | eugenol |
| Lonicera Japonica | 7-o-(4-β-d-glucopyranosyloxy-3-methoxy-benzoyl) secologanolicacid |
| Lonicera Japonica | α-pinene |
| Lonicera Japonica | 6'-o-(7α-hydroxyswerosyloxy)loganin |
| Lonicera Japonica | β-sitosterol-β-d-glucoside |
| Lonicera Japonica | (3r)-4'-methoxy-2',3,7-trihydroxyisoflavanone |
| Lonicera Japonica | loniflavone |
| Lonicera Japonica | lonicerin |
| Lonicera Japonica | loniceracetalide b |
| Lonicera Japonica | macrocarpal a |
| Lonicera Japonica | shuang-hua-chun |
| Lonicera Japonica | trans-2-hwxwnoic acid |
| Lonicera Japonica | farnesol |
| Lonicera Japonica | hederagenin 3-o-arabinoside |
| Lonicera Japonica | farnesyl acetate |
| Lonicera Japonica | inositol-b |
| Lonicera Japonica | nerol |
| Lonicera Japonica | inositol-c |
| Lonicera Japonica | hederagenin |
| Lonicera Japonica | benzyl benzoate |
| Lonicera Japonica | benzyl ethyl alcohol |
| Lonicera Japonica | linamarin |
| Lonicera Japonica | 1,1'-bicyclohexyl |
| Lonicera Japonica | linalool |
| Lonicera Japonica | inositol b |
| Lonicera Japonica | β-sitosterol |
| Lonicera Japonica | linalyl oxide |
| Lonicera Japonica | beta-pinene |
| Lonicera Japonica | neochlorogenic acid |
| Lonicera Japonica | dibutyl phthalate |
| Lonicera Japonica | ethyl palmitate |
| Lonicera Japonica | luteolin |
| Lonicera Japonica | chlorogenic acid |
| Lonicera Japonica | inositol c |
| Lonicera Japonica | menthyl acetate |
| Lonicera Japonica | inositol |
| Lonicera Japonica | caffeicacid |
| Lonicera Japonica | insularine |
| Lonicera Japonica | 2-heptanol |
| Lonicera Japonica | chrysoeriol |
| Lonicera Japonica | stigmasterol-beta-d-glucoside |
| Lonicera Japonica | isochlorogenic acid b |
| Lonicera Japonica | luteolin 7-o-beta-d-galatoside |
| Lonicera Japonica | loniceracetalide a |
| Lonicera Japonica | caffeic acid |
| Lonicera Japonica | macranthoidin a |
| Lonicera Japonica | macranthoidin b |
| Lonicera Japonica | macranthoin f |
| Lonicera Japonica | macranthoside a |
| Lonicera Japonica | macranthoside b |
| Lonicera Japonica | 3'-o-methyl loniflavone |
| Lonicera Japonica | pentanoic acid |
| Lonicera Japonica | methyllinolenate |
| Lonicera Japonica | methyl linoleate |
| Lonicera Japonica | delta-terpineol |
| Lonicera Japonica | stigmasterol |
| Lonicera Japonica | 2'-o-[β-d-apiofuranosyl(1→2)-β-d-glucopyra-nosyl]isoliquiritigenin |
| Lonicera Japonica | i-linalool |
| Lonicera Japonica | 3-methyl-2-(2-pentenyl)-2-cyclopenten-1-one |
| Lonicera Japonica | phenethyl alcohol |
| Lonicera Japonica | 3-methyl butanone |
| Lonicera Japonica | isochlorogenicacid |
| Lonicera Japonica | isochlorogenicacid a |
| Lonicera Japonica | loganicacid |
| Lonicera Japonica | 2-methyl-1-butanol |
| Lonicera Japonica | nerolidol |
| Lonicera Japonica | loganin |
| Lonicera Japonica | carvacrol acetate |
| Lonicera Japonica | carvacrol |
| Lonicera Japonica | (e)-aldosecologanin |
| Lonicera Japonica | (z)-aldosecologanin |
| Lonicera Japonica | benzaldehyde |
| Lonicera Japonica | methyl palmitate |
| Lonicera Japonica | 2-heptadecanone |
| Lonicera Japonica | 3-o-beta-d-glucopyranosyl-(1-3)-alpha-l-rhamnopyranosyl-(1-2)-alpha-l-arabinopyranosyl hederagenin 28-o-beta-d-gluco-pyranosyl-(1-6)-beta-d-glucopyranosyl ester(vii) |
| Lonicera Japonica | hexanoic acid |
| Lonicera Japonica | geraniol |
| Lonicera Japonica | geranyl acetate |
| Lonicera Japonica | phenylacetaldehyde |
| Lonicera Japonica | ethyl linolenate |
| Lonicera Japonica | 2-methylbutanoic acid |
| Lonicera Japonica | isoeugenol |
| Lonicera Japonica | methyl pentose(i) |
| Lonicera Japonica | beta-sitosterol-3-o-beta-d-xylopyranoside |
| Lonicera Japonica | ethyl-p-digallate |
| Lonicera Japonica | ethylpalmitate |
| Lonicera Japonica | 1-hexene |
| Lonicera Japonica | α-terpineol |
| Achyranthes Bidentata | inokosterone |
| Achyranthes Bidentata | ecdysterone |
| Myrrh | eugenol |
| Myrrh | cinnamaldehyde |
| Radix Aconiti Preparata | aconitine |
| Radix Aconiti Preparata | songorine |
| Radix Astragali | soyasapogenol b |
| Radix Astragali | isoastragaloside1, 3 |
| Radix Astragali | guanosine |
| Radix Astragali | formononentin |
| Radix Astragali | uridine |
| Radix Astragali | rhamnocitrin |
| Radix Astragali | 3,5-dimethoxystilbene |
| Radix Astragali | folinicacid |
| Radix Astragali | calycosin |
| Radix Astragali | folicacid |
| Radix Astragali | 2-hydroxy-3-methoxystrychnine |
| Radix Astragali | chrysanthemaxanthin |
| Radix Astragali | lupeol |
| Radix Astragali | cycloastragenol |
| Radix Astragali | kumugansine a |
| Radix Astragali | β-sitosterol |
| Radix Astragali | 9, 10-dimethoxypterocarpane-3-o-beta-d-glucoside |
| Radix Astragali | (3r) -2', 3' -dihydroxy-7, 4' -dimethoxyisoflavone |
| Radix Astragali | (−)-medicarpin |
| Radix Astragali | astragaloside vii |
| Radix Astragali | astragaloside viii |
| Radix Astragali | astragaloside v |
| Radix Astragali | astragaloside vi |
| Radix Astragali | astramembrannin ii |
| Radix Astragali | isoastragaloside ii |
| Radix Astragali | astramembrannin i |
| Radix Astragali | 4'-dimethoxyisoflavane-7-o-beta-d-glucoside |
| Radix Astragali | 3-o-beta-d-glucuronopyranosyl gypsogenin |
| Radix Astragali | acetyl astragaloside i |
| Radix Astragali | beta-sitosterol |
| Radix Astragali | 20(r)-21,24-cyclo-3beta,25-dihydroxyl-dammar-23(24)-en-21-one |
| Radix Astragali | gamma-sitosterol |
| Radix Astragali | formononetin |
| Radix Astragali | 2', 4' -dihydroxy-5 |
| Radix Astragali | 3'-hydroxy-4--methoxyisoflavone-7-o-beta-d-glucoside |
| Radix Astragali | adeninenucleoside |
| Radix Astragali | quercetin |
| Radix Astragali | 20-hexadecanoylingenol |
| Radix Astragali | choline |
| Radix Astragali | 6-dimethoxy-isoflavane |
| Radix Astragali | betaine |
| Radix Astragali | adenine |
| Radix Astragali | n-candicine |
| Radix Astragali | isoastragaloside i |
| Radix Astragali | 2'-hydroxy-3 |
| Radix Astragali | acetylastragaloside |
| Radix Astragali | astragaloside ii |
| Radix Astragali | astragaloside i |
| Radix Astragali | sucrose |
| Radix Astragali | 4-hydroxy-2,6-dimethyl-6-(3,7-dimethyl-2,6-octadienyl)-8-(3-methyl-2-butenyl)-2h-1-benzopyran-5,7(3h,6h)-dione |
| Radix Astragali | astragaloside iv |
| Radix Astragali | astragaloside iii |
| Radix Astragali | 2'-hydroxy-3',4'-dime thoxy-isoflavane-7-o-β-d-glucoside |
| Radix Astragali | hexadecanoicacid |
| Radix Astragali | canavanine |
| Radix Astragali | βetaine |
| Radix Astragali | foliosidine |
| Radix Astragali | isorhamnetin |
| Radix Astragali | cyclosieversigenin |
| Radix Astragali | folinic acid |
| Radix Astragali | 9,10-dimethoxy-pterocarpane-3-o-β-d-gluco-side |
| Radix Astragali | astragaloside 1~8 |
| Radix Astragali | soyasaponin 1 |
| Radix Astragali | suffruticoside a |
| Radix Astragali | kumatakenin |
| Radix Astragali | glucuronicacid |
| Radix Astragali | (6ar, 11ar) -10-hydroxy-3, 9-dimethoxypterocarpane |
| Radix Astragali | acetic acid |
| Radix Astragali | kaempferol |
| Radix Astragali | astramembrannin |
| Glycyrrhiza | rutin |
| Glycyrrhiza | schaftoside |
| Glycyrrhiza | isoschaftoside |
| Glycyrrhiza | 18alpha-glycyrrhetinic acid |
| Glycyrrhiza | narcissin |
| Glycyrrhiza | glycyrrhizin |
| Glycyrrhiza | isoquercitrin |
| Glycyrrhiza | astragalin |
| Glycyrrhiza | ononin |
| Glycyrrhiza | 18beta-glycyrrhetinic acid |
| Glycyrrhiza | glycyrrhetinic acid |
| Leech | gardnerilin a |
| Leech | gardenoside |
| Leech | croomionidine |
| Leech | ceramide-glycanase |
| Leech | crocin |
| Leech | genioisidic acid |
| Leech | hirudin-pa |
| Leech | genipinic acid |
| Leech | ursolic acid |
| Leech | enoxaparin |
| Leech | antithrombin |
| Leech | bdellin b-3 |
| Leech | geniposide |
| Leech | genipingentiobioside |
| Leech | cholesterol esterase |
| Leech | beta-acethlgalactorsaminidase |
| Leech | eglins |
| Leech | methyl(2e,8z)-decadien-4,6-diynoate |
| Leech | liquemin n |
| Leech | heparin |
| Leech | hirudin |
| Leech | lipase |
| Leech | o-desulfated heparin |
| Leech | dulcitol |
| Leech | destabilase |
| Leech | l-galactoheptulose |
| Leech | beta-ursolic acid |
| Leech | glycosphingolipid-splitting enzyme |
| Leech | bdellins |
| Leech | deacetyl asperulosidicacid methyl ester |
| Leech | propionylchlinesterase |
| Leech | d-mannoheptulose |
| Leech | crocetin |
| Leech | nadroparin |
| Leech | d-mannitol |
| Coptis | epiberberine |
| Coptis | obaculactone |
| Coptis | chlorogenic acid |
| Coptis | magnograndiolide |
| Coptis | trihydroxybufosterocholanic acid |
| Coptis | worenine |
| Coptis | 3,4-dihydroxyphenylethyl alcohol glucoside |
| Coptis | palmatine |
| Coptis | ferulic acid |
| Coptis | obakulactone |
| Coptis | berberine |
| Coptis | obamegine |
| Coptis | obacunoic acid |
| Coptis | palmidin a |
| Coptis | obakunone |
| Coptis | magnoflorine |
| Coptis | 2-carboxymethyl-3-prenyl-2,3-epoxy-1,4-naphthoquinone |
| Coptis | columbianadin |
| Coptis | columbamine |
| Coptis | 2,3,4-trihydroxy-benzenepropanoicacid |
| Coptis | coptisine |
| Coptis | corchoroside a |
| Coptis | limonin |
| Coptis | 3-carboxy-4-hydroxy-phenoxy glucoside |
| Coptis | 5,8-dihydroxy-2-(2-phenylethyl) chromone |
| Coptis | ferulicacid |
| Coptis | magnoflovine |
| Coptis | javanicin |
| Coptis | jatrorrhizine |
| Coptis | 6-o-e-feruloylajugol |

**Supplement Table S5** The detailed information of relationship between compounds and targets.

Different relationships between compounds and targets. True or false stands for whether the compound issues the action.

| **Compound** | **Target** | **Function** | **True or False** |
| --- | --- | --- | --- |
| (-)-epicatechin | IL6 | inhibition | t |
| (-)-epicatechin | NOS3 | binding | f |
| (-)-epicatechin | NOS1 | binding | f |
| (-)-epicatechin | HMOX1 | activation | t |
| (-)-epicatechin | NOS2 | binding | f |
| 18beta-glycyrrhetinic acid | ANPEP | inhibition | t |
| 18beta-glycyrrhetinic acid | SRC | activation | t |
| 18beta-glycyrrhetinic acid | ABCB1 | inhibition | t |
| 18beta-glycyrrhetinic acid | HSD11B2 | binding | f |
| 18beta-glycyrrhetinic acid | ICAM1 | inhibition | t |
| 18beta-glycyrrhetinic acid | NR3C1 | binding | f |
| 18beta-glycyrrhetinic acid | NOS2 | inhibition | f |
| 18beta-glycyrrhetinic acid | PTGR2 | binding | f |
| 18beta-glycyrrhetinic acid | REN | inhibition | t |
| 18beta-glycyrrhetinic acid | AKR1C3 | inhibition | t |
| 18beta-glycyrrhetinic acid | GJA1 | binding | f |
| 18beta-glycyrrhetinic acid | HSD11B1 | binding | f |
| 18beta-glycyrrhetinic acid | NR3C2 | binding | f |
| 2-furaldehyde | HBB | binding | f |
| 2-furaldehyde | HBA1 | binding | f |
| 2-methylbutanoic acid | CES1 | binding | f |
| acetic acid | PLAU | binding | f |
| acetic acid | PLA2G1B | inhibition | t |
| acetic acid | DOT1L | binding | f |
| acetic acid | ANPEP | binding | f |
| acetic acid | PEBP1 | binding | f |
| acetic acid | CANT1 | binding | f |
| acetic acid | ACSS3 | catalysis | f |
| acetic acid | FURIN | binding | f |
| acetic acid | TACR1 | reaction | f |
| acetic acid | SRC | binding | f |
| acetic acid | PCK1 | binding | f |
| acetic acid | FFAR1 | reaction | f |
| acetic acid | SLC16A1 | catalysis | f |
| acetic acid | LSM2 | binding | f |
| acetic acid | CCK | reaction | f |
| acetic acid | AVPR1A | reaction | f |
| acetic acid | CS | binding | f |
| acetic acid | TST | binding | f |
| acetic acid | PAICS | binding | f |
| acetic acid | NPSR1 | reaction | f |
| acetic acid | F12 | binding | f |
| acetic acid | ALDH3B2 | catalysis | f |
| acetic acid | ACYP1 | catalysis | f |
| acetic acid | FFAR2 | reaction | f |
| acetic acid | KLK1 | binding | f |
| acetic acid | ALDH1A1 | catalysis | f |
| acetic acid | GRPR | reaction | f |
| acetic acid | GM2A | binding | f |
| acetic acid | SMAD3 | binding | f |
| acetic acid | MTRR | binding | f |
| acetic acid | CTSD | binding | f |
| acetic acid | CDA | binding | f |
| acetic acid | PNP | binding | f |
| acetic acid | LDHA | binding | f |
| acetic acid | ACYP2 | catalysis | f |
| acetic acid | PROKR2 | reaction | f |
| acetic acid | HTR2A | reaction | f |
| acetic acid | CKB | binding | f |
| acetic acid | KISS1R | reaction | f |
| acetic acid | OXT | reaction | f |
| acetic acid | ADH7 | binding | f |
| acetic acid | MTAP | binding | f |
| acetic acid | TFB1M | binding | f |
| acetic acid | F2RL1 | reaction | f |
| acetic acid | ABAT | inhibition | t |
| acetic acid | UPP1 | binding | f |
| acetic acid | HLA-A | binding | f |
| acetic acid | SERPINE1 | binding | f |
| acetic acid | SDC3 | reaction | f |
| acetic acid | NOS1 | binding | f |
| acetic acid | VIM | binding | f |
| acetic acid | P2RY2 | reaction | f |
| acetic acid | CA2 | inhibition | t |
| acetic acid | PTEN | binding | f |
| acetic acid | PAFAH2 | catalysis | f |
| acetic acid | LEP | activation | f |
| acetic acid | DPYD | binding | f |
| acetic acid | VWF | binding | f |
| acetic acid | NDST3 | catalysis | f |
| acetic acid | KISS1 | reaction | f |
| acetic acid | APEX1 | binding | f |
| acetic acid | FNTB | binding | f |
| acetic acid | GNA14 | reaction | f |
| acetic acid | FPR2 | reaction | f |
| acetic acid | P2RY10 | reaction | f |
| acetic acid | CRYZ | binding | f |
| acetic acid | LIPE | binding | f |
| acetic acid | QRFPR | reaction | f |
| acetic acid | LSM6 | binding | f |
| acetic acid | SDC1 | reaction | f |
| acetic acid | TRH | reaction | f |
| acetic acid | ODC1 | activation | t |
| acetic acid | GBE1 | binding | f |
| acetic acid | TACR3 | reaction | f |
| acetic acid | UMPS | binding | f |
| acetic acid | GNMT | binding | f |
| acetic acid | FNTA | binding | f |
| acetic acid | CAT | binding | f |
| acetic acid | CDH1 | binding | f |
| acetic acid | CCND1 | inhibition | t |
| acetic acid | HAGH | binding | f |
| acetic acid | APP | reaction | f |
| acetic acid | PIM1 | binding | f |
| acetic acid | APCS | binding | f |
| acetic acid | OPN4 | reaction | f |
| acetic acid | SIAE | catalysis | f |
| acetic acid | EDN2 | reaction | f |
| acetic acid | JAK2 | binding | f |
| acetic acid | GRIA2 | binding | f |
| acetic acid | MAP2K1 | binding | f |
| acetic acid | LTA4H | binding | f |
| acetic acid | AGTR1 | reaction | f |
| acetic acid | HPGDS | binding | f |
| acetic acid | GNRHR | reaction | f |
| acetic acid | MAPK14 | binding | f |
| acetic acid | CD55 | binding | f |
| acetic acid | GNRH2 | reaction | f |
| acetic acid | PLK1 | binding | f |
| acetic acid | PRKAR2A | binding | f |
| acetic acid | ARFGEF1 | binding | f |
| acetic acid | GANC | binding | f |
| acetic acid | GRP | reaction | f |
| acetic acid | PTPN1 | binding | f |
| acetic acid | CASP6 | binding | f |
| acetic acid | PLA2G7 | catalysis | f |
| acetic acid | ACE | binding | f |
| acetic acid | CCKAR | reaction | f |
| acetic acid | MLNR | reaction | f |
| acetic acid | TNS1 | binding | f |
| acetic acid | KLF4 | binding | f |
| acetic acid | BSG | binding | f |
| acetic acid | AFMID | binding | f |
| acetic acid | SYT1 | binding | f |
| acetic acid | PON2 | catalysis | f |
| acetic acid | AGRN | reaction | f |
| acetic acid | EIF2AK2 | binding | f |
| acetic acid | NPFF | reaction | f |
| acetic acid | LPAR6 | reaction | f |
| acetic acid | LPA | binding | f |
| acetic acid | SLC16A8 | catalysis | f |
| acetic acid | HCRTR1 | reaction | f |
| acetic acid | ALDH9A1 | catalysis | f |
| acetic acid | GPR4 | reaction | f |
| acetic acid | GAST | reaction | f |
| acetic acid | CYSLTR2 | reaction | f |
| acetic acid | TRHR | reaction | f |
| acetic acid | GAA | binding | f |
| acetic acid | BACE1 | binding | f |
| acetic acid | ALB | binding | f |
| acetic acid | RETN | binding | f |
| acetic acid | DNMT3B | catalysis | f |
| acetic acid | LTB4R | reaction | f |
| acetic acid | CALM1 | binding | f |
| acetic acid | NPS | reaction | f |
| acetic acid | LYZ | binding | f |
| acetic acid | MYLIP | binding | f |
| acetic acid | LPAR1 | reaction | f |
| acetic acid | PLG | binding | f |
| acetic acid | GCGR | reaction | f |
| acetic acid | HDC | binding | f |
| acetic acid | PTS | binding | f |
| acetic acid | RNASE2 | binding | f |
| acetic acid | CHRM5 | reaction | f |
| acetic acid | ANXA1 | reaction | f |
| acetic acid | CBR4 | binding | f |
| acetic acid | PECR | binding | f |
| acetic acid | ACSS2 | catalysis | f |
| acetic acid | IMPDH2 | binding | f |
| acetic acid | AVP | reaction | f |
| acetic acid | EDNRB | reaction | f |
| acetic acid | HSPG2 | reaction | f |
| acetic acid | PCSK7 | binding | f |
| acetic acid | ADSSL1 | binding | f |
| acetic acid | LCT | binding | f |
| acetic acid | XCR1 | reaction | f |
| acetic acid | MAT2A | binding | f |
| acetic acid | S100A4 | binding | f |
| acetic acid | F2R | reaction | f |
| acetic acid | MLN | reaction | f |
| acetic acid | SDC4 | reaction | f |
| acetic acid | IL18 | activation | t |
| acetic acid | UTS2 | reaction | f |
| acetic acid | TBXA2R | reaction | f |
| acetic acid | NOS2 | binding | f |
| acetic acid | PAFAH1B1 | catalysis | f |
| acetic acid | CBS | binding | f |
| acetic acid | ASPA | binding | f |
| acetic acid | SIRT2 | binding | f |
| acetic acid | EDN3 | reaction | f |
| acetic acid | DHFR | binding | f |
| acetic acid | NTS | reaction | f |
| acetic acid | APRT | binding | f |
| acetic acid | DHFRL1 | binding | f |
| acetic acid | KNG1 | reaction | f |
| acetic acid | P2RY6 | reaction | f |
| acetic acid | CHDH | binding | f |
| acetic acid | YES1 | binding | f |
| acetic acid | CHRM3 | reaction | f |
| acetic acid | ALDH3A1 | catalysis | f |
| acetic acid | NDST2 | catalysis | f |
| acetic acid | MTHFD1 | binding | f |
| acetic acid | CFTR | binding | f |
| acetic acid | UCP2 | activation | t |
| acetic acid | SRPK2 | binding | f |
| acetic acid | CDK2 | binding | f |
| acetic acid | PROKR1 | reaction | f |
| acetic acid | PAFAH1B3 | catalysis | f |
| acetic acid | PLA2G2E | binding | f |
| acetic acid | ESR2 | binding | f |
| acetic acid | SORD | binding | f |
| acetic acid | HPRT1 | binding | f |
| acetic acid | CFH | binding | f |
| acetic acid | TYRO3 | binding | f |
| acetic acid | MTA1 | binding | f |
| acetic acid | MTR | binding | f |
| acetic acid | PRSS3 | binding | f |
| acetic acid | ACOT8 | catalysis | f |
| acetic acid | GPR17 | reaction | f |
| acetic acid | PRODH | binding | f |
| acetic acid | SDC2 | reaction | f |
| acetic acid | NDST1 | catalysis | f |
| acetic acid | REN | activation | t |
| acetic acid | M6PR | binding | f |
| acetic acid | QRFP | reaction | f |
| acetic acid | GSTP1 | binding | f |
| acetic acid | MPO | activation | t |
| acetic acid | UROD | binding | f |
| acetic acid | KIF5B | binding | f |
| acetic acid | GPC1 | reaction | f |
| acetic acid | FAH | binding | f |
| acetic acid | GCG | reaction | f |
| acetic acid | GNRH1 | reaction | f |
| acetic acid | PMCH | reaction | f |
| acetic acid | NTRK1 | binding | f |
| acetic acid | PAFAH1B2 | catalysis | f |
| acetic acid | ACHE | catalysis | f |
| acetic acid | ALDH2 | catalysis | f |
| acetic acid | AMDHD2 | catalysis | f |
| acetic acid | PPARA | activation | t |
| acetic acid | OXSM | binding | f |
| acetic acid | SERPINE2 | binding | f |
| acetic acid | PPOX | binding | f |
| acetic acid | PRTFDC1 | binding | f |
| acetic acid | PTAFR | reaction | f |
| acetic acid | PROK1 | reaction | f |
| acetic acid | GPRC6A | reaction | f |
| acetic acid | ISG20 | binding | f |
| acetic acid | DECR1 | binding | f |
| acetic acid | TJP1 | binding | f |
| acetic acid | AKR1C3 | inhibition | t |
| acetic acid | TTR | binding | f |
| acetic acid | GALM | binding | f |
| acetic acid | F2RL3 | reaction | f |
| acetic acid | CSNK2A1 | binding | f |
| acetic acid | IDH1 | activation | t |
| acetic acid | PROK2 | reaction | f |
| acetic acid | CLTC | binding | f |
| acetic acid | EDN1 | reaction | f |
| acetic acid | XDH | binding | f |
| acetic acid | ESR1 | binding | f |
| acetic acid | NTSR2 | reaction | f |
| acetic acid | PIGL | catalysis | f |
| acetic acid | NR1H2 | binding | f |
| acetic acid | GAD2 | binding | f |
| acetic acid | P2RY1 | reaction | f |
| acetic acid | NOS3 | inhibition | t |
| acetic acid | CHRM1 | reaction | f |
| acetic acid | TACR2 | reaction | f |
| acetic acid | MTHFR | binding | f |
| acetic acid | TAC3 | reaction | f |
| acetic acid | F2RL2 | reaction | f |
| acetic acid | MDP1 | binding | f |
| acetic acid | ADH4 | binding | f |
| acetic acid | HCRT | reaction | f |
| acetic acid | CCL2 | binding | f |
| acetic acid | HSPA5 | activation | t |
| acetic acid | NTSR1 | reaction | f |
| acetic acid | NDST4 | catalysis | f |
| acetic acid | IMPDH1 | binding | f |
| acetic acid | DDC | binding | f |
| acetic acid | PTGFR | reaction | f |
| acetic acid | ALDH3A2 | catalysis | f |
| acetic acid | BCHE | binding | f |
| acetic acid | CYSLTR1 | reaction | f |
| acetic acid | COMT | binding | f |
| acetic acid | ADSS | binding | f |
| acetic acid | PON1 | catalysis | f |
| acetic acid | QPCT | binding | f |
| acetic acid | DHODH | inhibition | t |
| acetic acid | GLA | binding | f |
| acetic acid | CLYBL | catalysis | f |
| acetic acid | NARFL | binding | f |
| acetic acid | TAC1 | reaction | f |
| acetic acid | GAPDH | binding | f |
| acetic acid | GPR68 | reaction | f |
| acetic acid | KLF2 | binding | f |
| acetic acid | SOD1 | inhibition | t |
| acetic acid | ADK | binding | f |
| acetic acid | ENTPD1 | binding | f |
| acetic acid | HSPA8 | binding | f |
| acetic acid | RNASE1 | binding | f |
| acetic acid | HSP90AA1 | binding | f |
| acetic acid | FOS | activation | t |
| acetic acid | TYMS | binding | f |
| acetic acid | MMP9 | binding | f |
| acetic acid | ANTXR2 | binding | f |
| acetic acid | TP53 | binding | f |
| acetic acid | TPI1 | binding | f |
| acetic acid | GPR132 | reaction | f |
| acetic acid | SLC16A7 | catalysis | f |
| acetic acid | GPR65 | reaction | f |
| acetic acid | GMPR2 | binding | f |
| acetic acid | F10 | binding | f |
| acetic acid | ACOT12 | catalysis | f |
| acetic acid | GNG2 | reaction | f |
| acetic acid | HSD11B1 | binding | f |
| acetic acid | SOD2 | binding | f |
| acetic acid | CASR | reaction | f |
| acetic acid | VEGFA | binding | f |
| acetic acid | SLC3A2 | binding | f |
| aconitine | KCNH2 | inhibition | t |
| adenine | STK24 | binding | f |
| adenine | PNP | binding | f |
| adenine | MTAP | catalysis | f |
| adenine | CHFR | binding | f |
| adenine | SLC29A2 | catalysis | f |
| adenine | XPNPEP1 | inhibition | t |
| adenine | PTGS2 | inhibition | t |
| adenine | ACP1 | binding | t |
| adenine | PNMT | binding | f |
| adenine | PECR | binding | t |
| adenine | APRT | binding | t |
| adenine | SRPK2 | binding | t |
| adenine | PYGM | binding | f |
| adenine | HPRT1 | inhibition | t |
| adenine | SLC29A3 | catalysis | f |
| adenine | ACVR2B | binding | f |
| adenine | P2RY1 | activation | t |
| adenine | ACACB | binding | t |
| adenine | HSP90AA1 | binding | f |
| allantoin | TTR | binding | f |
| allantoin | SLC2A4 | activation | t |
| astragaloside iv | TLR4 | inhibition | t |
| astragaloside iv | SERPINE1 | inhibition | t |
| astragaloside iv | ACE | inhibition | t |
| astragaloside iv | AKT1 | activation | t |
| astragaloside iv | GSK3B | inhibition | f |
| astragaloside iv | TP53 | inhibition | t |
| astramembrannin i | TLR4 | inhibition | t |
| astramembrannin i | SERPINE1 | inhibition | t |
| astramembrannin i | ACE | inhibition | t |
| astramembrannin i | AKT1 | activation | t |
| astramembrannin i | GSK3B | inhibition | f |
| astramembrannin i | TP53 | inhibition | t |
| benzaldehyde | MMP1 | activation | t |
| benzyl alcohol | LYZ | binding | f |
| benzyl alcohol | CES1 | catalysis | f |
| berberine | PLAU | inhibition | t |
| berberine | PLA2G1B | binding | f |
| berberine | TOP1 | inhibition | t |
| berberine | SRC | inhibition | t |
| berberine | TLR4 | inhibition | t |
| berberine | GATA2 | activation | t |
| berberine | AHR | activation | t |
| berberine | ALDH7A1 | activation | t |
| berberine | CYP1A1 | inhibition | t |
| berberine | PTPN1 | inhibition | t |
| berberine | PTGS2 | inhibition | t |
| berberine | STAT3 | inhibition | t |
| berberine | LPL | inhibition | t |
| berberine | ADIPOQ | activation | t |
| berberine | FN1 | inhibition | t |
| berberine | JUN | activation | t |
| berberine | AKT1 | inhibition | t |
| berberine | NOS2 | activation | f |
| berberine | UCP2 | activation | t |
| berberine | INSR | activation | t |
| berberine | AKR1B1 | inhibition | t |
| berberine | EGFR | inhibition | t |
| berberine | HMOX1 | activation | t |
| berberine | GCG | activation | t |
| berberine | ACHE | binding | f |
| berberine | STK11 | activation | t |
| berberine | LDLR | activation | t |
| berberine | PTGER4 | inhibition | t |
| berberine | SLC2A4 | activation | t |
| berberine | PON1 | activation | t |
| berberine | MMP2 | inhibition | t |
| berberine | MMP9 | inhibition | t |
| berberine | TP53 | activation | t |
| betaine | TLR4 | inhibition | t |
| betaine | IFNG | inhibition | f |
| betaine | ALDH7A1 | catalysis | f |
| betaine | BHMT2 | catalysis | f |
| betaine | CASP3 | inhibition | t |
| betaine | CHDH | binding | f |
| betaine | APOB | activation | t |
| betaine | ABR | inhibition | t |
| betaine | SLC6A13 | binding | f |
| betaine | BHMT | catalysis | f |
| betaine | SLC6A12 | catalysis | f |
| betaine | HSPA8 | binding | f |
| beta-sitosterol | ICAM1 | inhibition | t |
| beta-sitosterol | SREBF1 | inhibition | f |
| beta-sitosterol | CASP3 | activation | t |
| beta-sitosterol | APOE | activation | t |
| caffeic acid | ALOX15 | inhibition | t |
| caffeic acid | G6PD | inhibition | t |
| caffeic acid | TYR | catalysis | f |
| caffeic acid | MIF | binding | t |
| caffeic acid | MAPK8 | activation | t |
| caffeic acid | MPO | inhibition | t |
| caffeic acid | MAPK1 | binding | f |
| canavanine | NOS1 | binding | f |
| canavanine | NOS2 | binding | f |
| canavanine | NOS3 | inhibition | t |
| carvacrol | HTR2A | inhibition | t |
| carvacrol | HSPA4 | activation | t |
| carvacrol | CASP3 | activation | t |
| carvacrol | TRPA1 | activation | t |
| chlorogenic acid | DNMT1 | inhibition | t |
| chlorogenic acid | CASP3 | activation | t |
| chlorogenic acid | MAPK8 | inhibition | t |
| chlorogenic acid | HMGB1 | inhibition | t |
| choline | PLD6 | catalysis | f |
| choline | PLD3 | catalysis | f |
| choline | SLC22A1 | catalysis | f |
| choline | CHAT | catalysis | f |
| choline | ADH7 | catalysis | f |
| choline | CHKA | catalysis | f |
| choline | TNF | inhibition | t |
| choline | SLC5A7 | catalysis | f |
| choline | SLC22A2 | catalysis | f |
| choline | SLC44A3 | catalysis | f |
| choline | CHKB | catalysis | f |
| choline | LYZ | binding | f |
| choline | PCYT1A | binding | t |
| choline | S100A4 | activation | f |
| choline | PLD1 | binding | t |
| choline | CHDH | catalysis | f |
| choline | SLC44A2 | catalysis | f |
| choline | PLD2 | binding | t |
| choline | PCYT1B | catalysis | f |
| choline | REN | inhibition | t |
| choline | GCG | activation | t |
| choline | ACHE | catalysis | f |
| choline | PLD4 | catalysis | f |
| choline | BHMT | activation | t |
| choline | PTDSS1 | catalysis | f |
| choline | ADH4 | catalysis | f |
| choline | BCHE | binding | t |
| choline | SUV39H1 | activation | t |
| choline | BDNF | activation | t |
| choline | ETNK1 | binding | f |
| choline | SLC44A1 | catalysis | f |
| choline | SLC44A5 | catalysis | f |
| chrysoeriol | ABCC1 | inhibition | t |
| cinnamaldehyde | MAPK14 | activation | t |
| cinnamaldehyde | PTGS2 | inhibition | t |
| cinnamaldehyde | CASP3 | activation | t |
| cinnamaldehyde | MAPK8 | activation | t |
| cinnamaldehyde | MAPK3 | activation | t |
| cinnamaldehyde | NOS2 | inhibition | f |
| cinnamaldehyde | SLC2A4 | activation | t |
| cinnamaldehyde | MAPK1 | activation | t |
| crocetin | TIMP1 | activation | t |
| crocetin | COL1A1 | inhibition | t |
| crocetin | MAPK8 | inhibition | t |
| crocetin | TP53 | activation | t |
| crocin | NOS1 | inhibition | f |
| crocin | ADRBK1 | inhibition | t |
| crocin | NOS2 | inhibition | f |
| crocin | PNLIP | inhibition | t |
| crocin | HMOX1 | activation | t |
| crocin | MAPT | activation | t |
| crocin | NFE2L2 | activation | t |
| crocin | MMP9 | inhibition | t |
| dibutyl phthalate | PLA2G1B | binding | f |
| dibutyl phthalate | AR | activation | t |
| dibutyl phthalate | HSD11B2 | inhibition | t |
| dibutyl phthalate | NR1I3 | activation | t |
| dibutyl phthalate | TRPA1 | activation | t |
| dibutyl phthalate | NR1I2 | activation | t |
| dihydroquercetin | MTTP | inhibition | t |
| dihydroquercetin | TNFSF11 | inhibition | t |
| dihydroquercetin | APOB | inhibition | t |
| dihydroquercetin | NQO1 | activation | t |
| dihydroquercetin | ABCC1 | inhibition | t |
| dihydroresveratrol | LTA4H | binding | t |
| diosgenin | TNF | inhibition | t |
| diosgenin | CFLAR | inhibition | t |
| diosgenin | PTGS2 | inhibition | t |
| diosgenin | MAPK8 | inhibition | t |
| diosgenin | TNFRSF10B | activation | t |
| diosgenin | TIMP2 | activation | t |
| diosgenin | HGF | inhibition | t |
| diosgenin | MMP2 | activation | t |
| diosgenin | VEGFA | inhibition | t |
| d-mannitol | SRC | activation | t |
| d-mannitol | TLR4 | activation | f |
| d-mannitol | AQP4 | activation | t |
| d-mannitol | MTRR | binding | f |
| d-mannitol | NOS1 | binding | f |
| d-mannitol | TGFB1 | activation | t |
| d-mannitol | CASP3 | activation | t |
| d-mannitol | NOS2 | binding | f |
| d-mannitol | CASP9 | activation | t |
| d-mannitol | NOS3 | binding | f |
| dulcitol | AKR1B10 | catalysis | f |
| dulcitol | AKR1B1 | catalysis | f |
| enoxaparin | PLAU | activation | t |
| enoxaparin | SERPIND1 | binding | t |
| enoxaparin | ABCG2 | activation | t |
| enoxaparin | TLR4 | activation | t |
| enoxaparin | CXCR4 | inhibition | t |
| enoxaparin | KLK1 | inhibition | t |
| enoxaparin | S100A9 | inhibition | t |
| enoxaparin | SERPINE1 | inhibition | t |
| enoxaparin | KISS1 | activation | t |
| enoxaparin | SDC1 | activation | t |
| enoxaparin | PF4 | inhibition | t |
| enoxaparin | CDKN1B | activation | t |
| enoxaparin | EGR1 | inhibition | t |
| enoxaparin | CAT | activation | t |
| enoxaparin | SERPINC1 | activation | t |
| enoxaparin | BMP2 | inhibition | t |
| enoxaparin | PROC | activation | t |
| enoxaparin | IL2 | inhibition | t |
| enoxaparin | LPL | activation | t |
| enoxaparin | PLAT | activation | t |
| enoxaparin | ELN | inhibition | t |
| enoxaparin | BACE1 | inhibition | t |
| enoxaparin | ALB | activation | t |
| enoxaparin | CASP3 | inhibition | t |
| enoxaparin | ERBB2 | activation | t |
| enoxaparin | CXCL12 | inhibition | t |
| enoxaparin | IL1B | activation | t |
| enoxaparin | FN1 | activation | t |
| enoxaparin | MAPK3 | activation | t |
| enoxaparin | HSPG2 | inhibition | t |
| enoxaparin | PRL | activation | t |
| enoxaparin | TH | activation | t |
| enoxaparin | AZU1 | inhibition | t |
| enoxaparin | MDK | inhibition | t |
| enoxaparin | GSK3B | activation | t |
| enoxaparin | CFH | activation | t |
| enoxaparin | PPBP | inhibition | t |
| enoxaparin | MMP1 | inhibition | t |
| enoxaparin | FGF7 | activation | t |
| enoxaparin | VTN | activation | t |
| enoxaparin | SOD3 | activation | t |
| enoxaparin | REN | inhibition | t |
| enoxaparin | TNC | inhibition | t |
| enoxaparin | PRKD1 | activation | t |
| enoxaparin | CSNK2A1 | inhibition | t |
| enoxaparin | EGF | inhibition | t |
| enoxaparin | CKS2 | inhibition | t |
| enoxaparin | IGFBP3 | inhibition | t |
| enoxaparin | NOS3 | activation | t |
| enoxaparin | DAG1 | inhibition | t |
| enoxaparin | CSN3 | inhibition | t |
| enoxaparin | HGF | inhibition | t |
| enoxaparin | MMP2 | inhibition | t |
| enoxaparin | FOS | inhibition | t |
| enoxaparin | MMP9 | inhibition | t |
| enoxaparin | MAPK1 | activation | t |
| enoxaparin | F10 | inhibition | t |
| enoxaparin | FGF2 | activation | t |
| enoxaparin | TNNT2 | inhibition | t |
| enoxaparin | VEGFA | activation | t |
| epicatechin | NOS1 | binding | f |
| epicatechin | IL6 | inhibition | t |
| epicatechin | NOS2 | binding | f |
| epicatechin | HMOX1 | activation | t |
| epicatechin | NOS3 | binding | f |
| eugenol | TRPV1 | activation | t |
| eugenol | TRPV1 | activation | t |
| eugenol | CYP1A1 | inhibition | t |
| eugenol | CASP3 | activation | t |
| eugenol | CASP9 | activation | t |
| eugenol | TRPA1 | activation | t |
| eugenol | ALOX5 | inhibition | t |
| eugenol | KCNA5 | inhibition | t |
| eugenol | MAOA | inhibition | t |
| farnesol | MAOB | inhibition | t |
| farnesol | CASP3 | activation | t |
| ferulic acid | CYP1A2 | inhibition | t |
| ferulic acid | CYP1A1 | inhibition | t |
| ferulic acid | G6PD | inhibition | t |
| ferulic acid | MAPK3 | inhibition | t |
| ferulic acid | DHFR | inhibition | t |
| ferulic acid | DECR1 | inhibition | t |
| ferulic acid | CYCS | activation | t |
| ferulic acid | MAPK1 | inhibition | t |
| folinic acid | FPGS | binding | f |
| folinic acid | DHFR | binding | f |
| folinic acid | SLC19A1 | binding | f |
| folinic acid | MTHFR | inhibition | t |
| folinic acid | MTHFS | binding | f |
| folinic acid | TYMS | binding | f |
| geniposide | IL6 | inhibition | t |
| geniposide | SELE | inhibition | t |
| geniposide | GSK3B | activation | f |
| geniposide | HMOX1 | activation | t |
| geniposide | CYP2E1 | inhibition | t |
| geraniol | CDK2 | inhibition | t |
| glycyrrhetinic acid | ANPEP | inhibition | t |
| glycyrrhetinic acid | SRC | activation | t |
| glycyrrhetinic acid | ABCB1 | inhibition | t |
| glycyrrhetinic acid | HSD11B2 | binding | f |
| glycyrrhetinic acid | ICAM1 | inhibition | t |
| glycyrrhetinic acid | NR3C1 | binding | f |
| glycyrrhetinic acid | NOS2 | inhibition | f |
| glycyrrhetinic acid | PTGR2 | binding | f |
| glycyrrhetinic acid | REN | inhibition | t |
| glycyrrhetinic acid | AKR1C3 | inhibition | t |
| glycyrrhetinic acid | GJA1 | binding | f |
| glycyrrhetinic acid | HSD11B1 | binding | f |
| glycyrrhetinic acid | NR3C2 | binding | f |
| glycyrrhizin | SRC | inhibition | t |
| glycyrrhizin | HSD11B2 | binding | f |
| glycyrrhizin | NOS1 | inhibition | f |
| glycyrrhizin | TLR2 | inhibition | t |
| glycyrrhizin | GSK3B | inhibition | f |
| glycyrrhizin | GJA1 | inhibition | t |
| glycyrrhizin | HSD11B1 | binding | f |
| heparin | SERPIND1 | binding | t |
| heparin | ADIPOQ | activation | t |
| hexanoic acid | TACR1 | reaction | f |
| hexanoic acid | FFAR1 | reaction | f |
| hexanoic acid | EDNRA | reaction | f |
| hexanoic acid | CCK | reaction | f |
| hexanoic acid | AVPR1A | reaction | f |
| hexanoic acid | NPSR1 | reaction | f |
| hexanoic acid | FFAR2 | binding | f |
| hexanoic acid | GRPR | reaction | f |
| hexanoic acid | PROKR2 | reaction | f |
| hexanoic acid | HTR2A | reaction | f |
| hexanoic acid | KISS1R | reaction | f |
| hexanoic acid | OXT | reaction | f |
| hexanoic acid | F2RL1 | reaction | f |
| hexanoic acid | P2RY2 | reaction | f |
| hexanoic acid | KISS1 | reaction | f |
| hexanoic acid | GNA14 | reaction | f |
| hexanoic acid | FPR2 | reaction | f |
| hexanoic acid | P2RY10 | reaction | f |
| hexanoic acid | QRFPR | reaction | f |
| hexanoic acid | TRH | reaction | f |
| hexanoic acid | TACR3 | reaction | f |
| hexanoic acid | APP | reaction | f |
| hexanoic acid | OPN4 | reaction | f |
| hexanoic acid | EDN2 | reaction | f |
| hexanoic acid | AGTR1 | reaction | f |
| hexanoic acid | GNRHR | reaction | f |
| hexanoic acid | GNRH2 | reaction | f |
| hexanoic acid | LPAR2 | reaction | f |
| hexanoic acid | GRP | reaction | f |
| hexanoic acid | CCKAR | reaction | f |
| hexanoic acid | MLNR | reaction | f |
| hexanoic acid | NPFF | reaction | f |
| hexanoic acid | LPAR6 | reaction | f |
| hexanoic acid | HCRTR1 | reaction | f |
| hexanoic acid | GPR4 | reaction | f |
| hexanoic acid | GAST | reaction | f |
| hexanoic acid | CYSLTR2 | reaction | f |
| hexanoic acid | TRHR | reaction | f |
| hexanoic acid | OXTR | reaction | f |
| hexanoic acid | LTB4R | reaction | f |
| hexanoic acid | NPS | reaction | f |
| hexanoic acid | NMBR | reaction | f |
| hexanoic acid | ADRA1D | reaction | f |
| hexanoic acid | LPAR1 | reaction | f |
| hexanoic acid | GCGR | reaction | f |
| hexanoic acid | HTR2B | reaction | f |
| hexanoic acid | CCKBR | reaction | f |
| hexanoic acid | CHRM5 | reaction | f |
| hexanoic acid | ANXA1 | reaction | f |
| hexanoic acid | AVP | reaction | f |
| hexanoic acid | EDNRB | reaction | f |
| hexanoic acid | XCR1 | reaction | f |
| hexanoic acid | F2R | reaction | f |
| hexanoic acid | MLN | reaction | f |
| hexanoic acid | UTS2 | reaction | f |
| hexanoic acid | TBXA2R | reaction | f |
| hexanoic acid | UTS2R | reaction | f |
| hexanoic acid | EDN3 | reaction | f |
| hexanoic acid | NTS | reaction | f |
| hexanoic acid | KNG1 | reaction | f |
| hexanoic acid | P2RY6 | reaction | f |
| hexanoic acid | PTGER1 | reaction | f |
| hexanoic acid | CHRM3 | reaction | f |
| hexanoic acid | NPFFR1 | reaction | f |
| hexanoic acid | PROKR1 | reaction | f |
| hexanoic acid | AKR1B1 | binding | f |
| hexanoic acid | GPR17 | reaction | f |
| hexanoic acid | QRFP | reaction | f |
| hexanoic acid | ADRA1B | reaction | f |
| hexanoic acid | GNA15 | reaction | f |
| hexanoic acid | GCG | reaction | f |
| hexanoic acid | GNRH1 | reaction | f |
| hexanoic acid | PMCH | reaction | f |
| hexanoic acid | LPAR3 | reaction | f |
| hexanoic acid | OXSM | binding | f |
| hexanoic acid | PTAFR | reaction | f |
| hexanoic acid | PROK1 | reaction | f |
| hexanoic acid | GPRC6A | reaction | f |
| hexanoic acid | F2RL3 | reaction | f |
| hexanoic acid | PROK2 | reaction | f |
| hexanoic acid | EDN1 | reaction | f |
| hexanoic acid | NTSR2 | reaction | f |
| hexanoic acid | P2RY1 | reaction | f |
| hexanoic acid | CHRM1 | reaction | f |
| hexanoic acid | TACR2 | reaction | f |
| hexanoic acid | BDKRB1 | reaction | f |
| hexanoic acid | TAC3 | reaction | f |
| hexanoic acid | F2RL2 | reaction | f |
| hexanoic acid | HCRT | reaction | f |
| hexanoic acid | CELA1 | binding | f |
| hexanoic acid | NTSR1 | reaction | f |
| hexanoic acid | PTGFR | reaction | f |
| hexanoic acid | CYSLTR1 | reaction | f |
| hexanoic acid | NPFFR2 | reaction | f |
| hexanoic acid | TAC1 | reaction | f |
| hexanoic acid | GPR68 | reaction | f |
| hexanoic acid | HTR2C | reaction | f |
| hexanoic acid | GPR132 | reaction | f |
| hexanoic acid | GPR65 | reaction | f |
| hexanoic acid | GNG2 | reaction | f |
| hexanoic acid | CASR | reaction | f |
| inositol | GBA | inhibition | t |
| inositol | HTR2A | inhibition | t |
| inositol | SLC5A3 | catalysis | f |
| inositol | GDE1 | catalysis | f |
| inositol | CDIPT | inhibition | t |
| inositol | IMPAD1 | catalysis | f |
| inositol | SLC5A11 | catalysis | f |
| inositol | AKT1 | inhibition | t |
| inositol | MIOX | catalysis | f |
| inositol | SLC2A13 | catalysis | f |
| inositol | GLA | catalysis | f |
| inositol | LTF | binding | f |
| isoeugenol | AHR | activation | t |
| isoeugenol | IL2 | inhibition | f |
| isoeugenol | MPO | binding | f |
| isoeugenol | LPO | binding | f |
| isoquercitrin | CYP1A1 | inhibition | t |
| isoquercitrin | CASP3 | activation | t |
| isorhamnetin | PON2 | activation | t |
| isorhamnetin | MAPK8 | inhibition | t |
| isorhamnetin | AKT1 | inhibition | t |
| isorhamnetin | NOS2 | inhibition | f |
| isorhamnetin | HMOX1 | activation | t |
| isorhamnetin | ABCC1 | inhibition | t |
| jatrorrhizine | SELE | inhibition | t |
| kaempferol | SRC | inhibition | t |
| kaempferol | ALOX12 | inhibition | t |
| kaempferol | NOS1 | inhibition | f |
| kaempferol | AHR | activation | t |
| kaempferol | CYP1A1 | inhibition | t |
| kaempferol | STAT1 | inhibition | t |
| kaempferol | NR1I3 | activation | t |
| kaempferol | IL2 | inhibition | t |
| kaempferol | STAT3 | inhibition | t |
| kaempferol | TNFRSF11B | activation | t |
| kaempferol | PTGES | inhibition | t |
| kaempferol | JUN | inhibition | t |
| kaempferol | AKT1 | inhibition | t |
| kaempferol | CYP2B6 | inhibition | t |
| kaempferol | CDK2 | inhibition | t |
| kaempferol | ESR2 | activation | t |
| kaempferol | MMP1 | inhibition | t |
| kaempferol | GSTP1 | inhibition | t |
| kaempferol | HMOX1 | activation | t |
| kaempferol | UGT3A1 | binding | t |
| kaempferol | SLC2A1 | inhibition | t |
| kaempferol | NR1I2 | activation | t |
| kaempferol | RB1 | inhibition | t |
| kaempferol | IGF2 | inhibition | t |
| kaempferol | CCL2 | inhibition | t |
| kaempferol | ABCC1 | inhibition | t |
| kaempferol | MMP2 | inhibition | t |
| l-galactoheptulose | SHPK | catalysis | f |
| linalool | NOS1 | inhibition | f |
| lupeol | CTNNB1 | inhibition | t |
| lupeol | MITF | activation | t |
| lupeol | TP53 | activation | t |
| luteolin | TOP1 | inhibition | t |
| luteolin | PCK1 | inhibition | f |
| luteolin | TLR4 | inhibition | t |
| luteolin | NOS1 | inhibition | f |
| luteolin | BCL2L1 | inhibition | t |
| luteolin | CDH1 | activation | t |
| luteolin | PPARG | binding | f |
| luteolin | CASP3 | activation | t |
| luteolin | ERBB2 | inhibition | f |
| luteolin | MAPK8 | inhibition | t |
| luteolin | IL1B | inhibition | t |
| luteolin | FN1 | inhibition | t |
| luteolin | MAPK3 | inhibition | t |
| luteolin | PKM2 | activation | t |
| luteolin | JUN | inhibition | t |
| luteolin | AKT1 | activation | t |
| luteolin | NOS2 | inhibition | f |
| luteolin | FOXO1 | activation | t |
| luteolin | CASP9 | activation | t |
| luteolin | TBK1 | inhibition | t |
| luteolin | ESR2 | activation | t |
| luteolin | HMOX1 | activation | t |
| luteolin | MTOR | inhibition | t |
| luteolin | TTR | binding | f |
| luteolin | CSNK2A1 | binding | f |
| luteolin | TNFRSF10B | activation | t |
| luteolin | JUNB | inhibition | t |
| luteolin | IGF2 | inhibition | t |
| luteolin | ABCC1 | inhibition | t |
| luteolin | RPS6KA1 | inhibition | t |
| luteolin | IGF1 | inhibition | t |
| luteolin | HSP90AA1 | inhibition | t |
| luteolin | FOS | inhibition | t |
| luteolin | NFE2L2 | inhibition | t |
| luteolin | MMP9 | inhibition | t |
| luteolin | TP53 | activation | t |
| luteolin | MAPK1 | inhibition | t |
| luteolin | CASP7 | activation | t |
| luteolin | VEGFA | inhibition | t |
| obaculactone | CHRM4 | reaction | f |
| obaculactone | CXCR4 | reaction | f |
| obaculactone | CCL21 | reaction | f |
| obaculactone | CNR1 | reaction | f |
| obaculactone | FPR2 | reaction | f |
| obaculactone | IL8 | reaction | f |
| obaculactone | GAL | reaction | f |
| obaculactone | ADRA2C | reaction | f |
| obaculactone | APP | reaction | f |
| obaculactone | ADRA2A | reaction | f |
| obaculactone | NPY | reaction | f |
| obaculactone | LPAR2 | reaction | f |
| obaculactone | P2RY4 | reaction | f |
| obaculactone | LPAR1 | reaction | f |
| obaculactone | CYP3A4 | inhibition | t |
| obaculactone | POMC | reaction | f |
| obaculactone | CXCL12 | reaction | f |
| obaculactone | ANXA1 | reaction | f |
| obaculactone | KNG1 | reaction | f |
| obaculactone | PPBP | reaction | f |
| obaculactone | GPR17 | reaction | f |
| obaculactone | DRD4 | reaction | f |
| obaculactone | PMCH | reaction | f |
| obaculactone | LPAR3 | reaction | f |
| obaculactone | CCR2 | reaction | f |
| obaculactone | BDKRB1 | reaction | f |
| obaculactone | OPRD1 | reaction | f |
| obaculactone | C5AR1 | reaction | f |
| obaculactone | CXCL10 | reaction | f |
| obaculactone | GNG2 | reaction | f |
| obaculactone | CASR | reaction | f |
| palmatine | TOP1 | inhibition | t |
| pentanoic acid | TACR1 | reaction | f |
| pentanoic acid | FFAR1 | reaction | f |
| pentanoic acid | EDNRA | reaction | f |
| pentanoic acid | CCK | reaction | f |
| pentanoic acid | AVPR1A | reaction | f |
| pentanoic acid | NPSR1 | reaction | f |
| pentanoic acid | FFAR2 | binding | f |
| pentanoic acid | GRPR | reaction | f |
| pentanoic acid | PROKR2 | reaction | f |
| pentanoic acid | HTR2A | reaction | f |
| pentanoic acid | KISS1R | reaction | f |
| pentanoic acid | OXT | reaction | f |
| pentanoic acid | F2RL1 | reaction | f |
| pentanoic acid | P2RY2 | reaction | f |
| pentanoic acid | KISS1 | reaction | f |
| pentanoic acid | GNA14 | reaction | f |
| pentanoic acid | FPR2 | reaction | f |
| pentanoic acid | P2RY10 | reaction | f |
| pentanoic acid | QRFPR | reaction | f |
| pentanoic acid | TRH | reaction | f |
| pentanoic acid | TACR3 | reaction | f |
| pentanoic acid | APP | reaction | f |
| pentanoic acid | OPN4 | reaction | f |
| pentanoic acid | EDN2 | reaction | f |
| pentanoic acid | AGTR1 | reaction | f |
| pentanoic acid | GNRHR | reaction | f |
| pentanoic acid | GNRH2 | reaction | f |
| pentanoic acid | LPAR2 | reaction | f |
| pentanoic acid | GRP | reaction | f |
| pentanoic acid | CCKAR | reaction | f |
| pentanoic acid | MLNR | reaction | f |
| pentanoic acid | NPFF | reaction | f |
| pentanoic acid | LPAR6 | reaction | f |
| pentanoic acid | HCRTR1 | reaction | f |
| pentanoic acid | GPR4 | reaction | f |
| pentanoic acid | GAST | reaction | f |
| pentanoic acid | CYSLTR2 | reaction | f |
| pentanoic acid | TRHR | reaction | f |
| pentanoic acid | OXTR | reaction | f |
| pentanoic acid | LTB4R | reaction | f |
| pentanoic acid | NPS | reaction | f |
| pentanoic acid | NMBR | reaction | f |
| pentanoic acid | ADRA1D | reaction | f |
| pentanoic acid | LPAR1 | reaction | f |
| pentanoic acid | GCGR | reaction | f |
| pentanoic acid | HTR2B | reaction | f |
| pentanoic acid | CCKBR | reaction | f |
| pentanoic acid | CHRM5 | reaction | f |
| pentanoic acid | ANXA1 | reaction | f |
| pentanoic acid | AVP | reaction | f |
| pentanoic acid | EDNRB | reaction | f |
| pentanoic acid | XCR1 | reaction | f |
| pentanoic acid | F2R | reaction | f |
| pentanoic acid | MLN | reaction | f |
| pentanoic acid | UTS2 | reaction | f |
| pentanoic acid | TBXA2R | reaction | f |
| pentanoic acid | UTS2R | reaction | f |
| pentanoic acid | EDN3 | reaction | f |
| pentanoic acid | NTS | reaction | f |
| pentanoic acid | KNG1 | reaction | f |
| pentanoic acid | P2RY6 | reaction | f |
| pentanoic acid | PTGER1 | reaction | f |
| pentanoic acid | CHRM3 | reaction | f |
| pentanoic acid | NPFFR1 | reaction | f |
| pentanoic acid | PROKR1 | reaction | f |
| pentanoic acid | GPR17 | reaction | f |
| pentanoic acid | QRFP | reaction | f |
| pentanoic acid | ADRA1B | reaction | f |
| pentanoic acid | GNA15 | reaction | f |
| pentanoic acid | GCG | reaction | f |
| pentanoic acid | GNRH1 | reaction | f |
| pentanoic acid | PMCH | reaction | f |
| pentanoic acid | LPAR3 | reaction | f |
| pentanoic acid | PTAFR | reaction | f |
| pentanoic acid | PROK1 | reaction | f |
| pentanoic acid | GPRC6A | reaction | f |
| pentanoic acid | F2RL3 | reaction | f |
| pentanoic acid | PROK2 | reaction | f |
| pentanoic acid | EDN1 | reaction | f |
| pentanoic acid | NTSR2 | reaction | f |
| pentanoic acid | P2RY1 | reaction | f |
| pentanoic acid | CHRM1 | reaction | f |
| pentanoic acid | TACR2 | reaction | f |
| pentanoic acid | BDKRB1 | reaction | f |
| pentanoic acid | TAC3 | reaction | f |
| pentanoic acid | F2RL2 | reaction | f |
| pentanoic acid | HCRT | reaction | f |
| pentanoic acid | NTSR1 | reaction | f |
| pentanoic acid | PTGFR | reaction | f |
| pentanoic acid | CYSLTR1 | reaction | f |
| pentanoic acid | NPFFR2 | reaction | f |
| pentanoic acid | TAC1 | reaction | f |
| pentanoic acid | GPR68 | reaction | f |
| pentanoic acid | HTR2C | reaction | f |
| pentanoic acid | GPR132 | reaction | f |
| pentanoic acid | GPR65 | reaction | f |
| pentanoic acid | GNG2 | reaction | f |
| pentanoic acid | CASR | reaction | f |
| phenylacetaldehyde | FURIN | binding | f |
| phenylacetaldehyde | ALDH3B2 | catalysis | f |
| phenylacetaldehyde | MAOB | catalysis | f |
| phenylacetaldehyde | AOC2 | catalysis | f |
| phenylacetaldehyde | ALDH1A3 | catalysis | f |
| phenylacetaldehyde | ALDH3A1 | catalysis | f |
| phenylacetaldehyde | AOC3 | catalysis | f |
| phenylacetaldehyde | ALDH2 | catalysis | f |
| phenylacetaldehyde | MAOA | catalysis | f |
| phenylacetaldehyde | ALDH3A2 | catalysis | f |
| quercetin | SERPIND1 | activation | t |
| quercetin | CYP1A2 | inhibition | f |
| quercetin | STK17B | catalysis | f |
| quercetin | CXCR4 | inhibition | t |
| quercetin | ABCA1 | activation | t |
| quercetin | AR | activation | t |
| quercetin | CYP2C8 | inhibition | t |
| quercetin | ALOX12 | inhibition | t |
| quercetin | NOS1 | inhibition | f |
| quercetin | SIRT1 | activation | t |
| quercetin | BAX | activation | t |
| quercetin | AHR | activation | t |
| quercetin | ODC1 | inhibition | t |
| quercetin | PIM1 | catalysis | f |
| quercetin | HPGDS | inhibition | t |
| quercetin | ICAM1 | inhibition | t |
| quercetin | NR1I3 | activation | t |
| quercetin | CTNNB1 | inhibition | t |
| quercetin | PTGS2 | inhibition | t |
| quercetin | TYR | activation | t |
| quercetin | PON2 | activation | t |
| quercetin | ATP5C1 | binding | t |
| quercetin | ADIPOQ | activation | t |
| quercetin | SLC12A2 | activation | t |
| quercetin | CASP3 | inhibition | t |
| quercetin | MAPK8 | inhibition | t |
| quercetin | PIK3CG | binding | t |
| quercetin | ATP5A1 | binding | t |
| quercetin | TLR2 | inhibition | t |
| quercetin | MAPK3 | activation | t |
| quercetin | NOS2 | inhibition | f |
| quercetin | CASP9 | activation | f |
| quercetin | CFTR | activation | t |
| quercetin | CSF2 | activation | t |
| quercetin | CDK2 | inhibition | t |
| quercetin | PYGM | binding | f |
| quercetin | ATP5B | catalysis | f |
| quercetin | TBK1 | inhibition | t |
| quercetin | MMP1 | inhibition | t |
| quercetin | GADD45A | activation | t |
| quercetin | AKR1B1 | binding | f |
| quercetin | PYGB | binding | f |
| quercetin | SULT1E1 | inhibition | f |
| quercetin | GSTP1 | inhibition | t |
| quercetin | HMOX1 | activation | t |
| quercetin | UGT3A1 | binding | t |
| quercetin | SLC2A1 | inhibition | t |
| quercetin | TGFA | inhibition | t |
| quercetin | AKR1C3 | inhibition | t |
| quercetin | NR1I2 | activation | t |
| quercetin | PYGL | binding | f |
| quercetin | TNFRSF10B | activation | t |
| quercetin | IGFBP3 | activation | t |
| quercetin | HIF1A | activation | t |
| quercetin | XDH | binding | f |
| quercetin | ESR1 | activation | t |
| quercetin | CYP2E1 | inhibition | t |
| quercetin | ALOX5 | binding | f |
| quercetin | MMP3 | inhibition | t |
| quercetin | MAOA | binding | f |
| quercetin | NPC1L1 | inhibition | t |
| quercetin | PON1 | activation | t |
| quercetin | MMP2 | activation | t |
| quercetin | BDNF | activation | t |
| quercetin | FOS | inhibition | t |
| quercetin | NFE2L2 | activation | t |
| quercetin | PARP1 | inhibition | t |
| quercetin | TP53 | activation | t |
| quercetin | PTGS1 | inhibition | t |
| quercetin | DIO2 | activation | t |
| quercetin | VEGFA | inhibition | t |
| quercetin | HCK | binding | t |
| resveratrol | CYP1A2 | inhibition | f |
| resveratrol | SRC | inhibition | t |
| resveratrol | PCK1 | activation | f |
| resveratrol | AMD1 | inhibition | t |
| resveratrol | PGR | inhibition | t |
| resveratrol | CS | activation | t |
| resveratrol | NQO2 | catalysis | f |
| resveratrol | ALDH1A1 | inhibition | t |
| resveratrol | ELAVL1 | activation | t |
| resveratrol | PRKCA | inhibition | t |
| resveratrol | SMAD3 | inhibition | t |
| resveratrol | SCNN1A | inhibition | f |
| resveratrol | ALOX12 | inhibition | t |
| resveratrol | NOS1 | inhibition | f |
| resveratrol | HSPA4 | inhibition | t |
| resveratrol | PTEN | activation | t |
| resveratrol | APEX1 | inhibition | t |
| resveratrol | IL8 | inhibition | t |
| resveratrol | STAB2 | activation | t |
| resveratrol | PDE5A | inhibition | t |
| resveratrol | EGR1 | activation | t |
| resveratrol | CAT | inhibition | t |
| resveratrol | CDH1 | activation | t |
| resveratrol | CCND1 | inhibition | t |
| resveratrol | CYP1A1 | inhibition | t |
| resveratrol | NPY | inhibition | t |
| resveratrol | TGFB1 | inhibition | t |
| resveratrol | MAP2K1 | activation | t |
| resveratrol | LTA4H | binding | f |
| resveratrol | AGTR1 | inhibition | t |
| resveratrol | PPARG | binding | f |
| resveratrol | MAPK14 | activation | t |
| resveratrol | NR1I3 | activation | t |
| resveratrol | PTPN1 | inhibition | t |
| resveratrol | CASP6 | activation | t |
| resveratrol | CTNNB1 | activation | t |
| resveratrol | ACE | inhibition | t |
| resveratrol | PTGS2 | binding | t |
| resveratrol | BSG | inhibition | t |
| resveratrol | G6PD | activation | t |
| resveratrol | TYR | inhibition | t |
| resveratrol | PRKAA2 | activation | t |
| resveratrol | ATP5C1 | binding | f |
| resveratrol | SIRT7 | activation | t |
| resveratrol | PRKAA1 | activation | t |
| resveratrol | PTPN11 | activation | t |
| resveratrol | ADIPOQ | activation | t |
| resveratrol | MAPK8 | inhibition | t |
| resveratrol | PIK3CG | inhibition | t |
| resveratrol | GPX1 | activation | t |
| resveratrol | CUL5 | activation | t |
| resveratrol | POMC | inhibition | t |
| resveratrol | ATP5A1 | binding | f |
| resveratrol | KLK3 | inhibition | t |
| resveratrol | CBR4 | activation | t |
| resveratrol | TLR2 | inhibition | t |
| resveratrol | CREB1 | activation | t |
| resveratrol | MAPK3 | inhibition | t |
| resveratrol | PKM2 | inhibition | t |
| resveratrol | BGLAP | activation | f |
| resveratrol | JUN | activation | t |
| resveratrol | IL18 | inhibition | t |
| resveratrol | TBXA2R | inhibition | t |
| resveratrol | PFKM | inhibition | t |
| resveratrol | RELA | inhibition | t |
| resveratrol | FOXO1 | activation | t |
| resveratrol | SULT1B1 | binding | f |
| resveratrol | CASP9 | inhibition | t |
| resveratrol | CFTR | activation | t |
| resveratrol | TRPA1 | inhibition | f |
| resveratrol | CDK2 | inhibition | t |
| resveratrol | ATP5B | binding | f |
| resveratrol | GSK3A | activation | t |
| resveratrol | PRNP | inhibition | t |
| resveratrol | GSK3B | activation | t |
| resveratrol | FASLG | activation | t |
| resveratrol | SULT1E1 | inhibition | t |
| resveratrol | HMOX1 | inhibition | t |
| resveratrol | FOXO3 | activation | t |
| resveratrol | MTOR | inhibition | t |
| resveratrol | PPARA | activation | t |
| resveratrol | SERPINE2 | inhibition | f |
| resveratrol | PRKD1 | inhibition | t |
| resveratrol | NR1I2 | activation | t |
| resveratrol | CSNK2A1 | catalysis | f |
| resveratrol | TNFRSF10B | activation | t |
| resveratrol | EGF | inhibition | t |
| resveratrol | ELANE | inhibition | t |
| resveratrol | HIF1A | inhibition | t |
| resveratrol | ESR1 | binding | f |
| resveratrol | CYP2E1 | inhibition | t |
| resveratrol | NOS3 | activation | t |
| resveratrol | CCR2 | inhibition | f |
| resveratrol | CD4 | inhibition | t |
| resveratrol | SLC2A4 | activation | t |
| resveratrol | SPHK1 | inhibition | t |
| resveratrol | PON1 | activation | t |
| resveratrol | SP1 | activation | t |
| resveratrol | IGF1 | inhibition | t |
| resveratrol | BDNF | activation | t |
| resveratrol | FOS | inhibition | t |
| resveratrol | NFE2L2 | activation | t |
| resveratrol | PARP1 | inhibition | t |
| resveratrol | TP53 | activation | t |
| resveratrol | MAPK1 | inhibition | t |
| resveratrol | CD97 | inhibition | t |
| resveratrol | PTGS1 | inhibition | t |
| resveratrol | DIO2 | inhibition | t |
| resveratrol | SOD2 | activation | t |
| resveratrol | CEL | inhibition | t |
| rutin | P4HB | inhibition | t |
| rutin | HSPA4 | inhibition | t |
| rutin | SREBF1 | inhibition | t |
| rutin | CASP3 | activation | t |
| rutin | GSR | inhibition | t |
| rutin | NOS2 | inhibition | f |
| rutin | PRNP | inhibition | t |
| rutin | EGFR | inhibition | t |
| rutin | ALDH2 | activation | t |
| rutin | AKR1C3 | catalysis | f |
| rutin | FGF2 | activation | f |
| rutin | CASP7 | activation | t |
| sucrose | TXNL4B | binding | f |
| sucrose | NNT | binding | f |
| sucrose | RAD1 | binding | f |
| sucrose | SLC2A5 | activation | t |
| sucrose | FNTB | binding | f |
| sucrose | RNF4 | binding | f |
| sucrose | FNTA | binding | f |
| sucrose | AGTR1 | inhibition | t |
| sucrose | GANC | catalysis | f |
| sucrose | TAS1R2 | binding | f |
| sucrose | GAA | catalysis | f |
| sucrose | LYZ | binding | f |
| sucrose | CES1 | binding | f |
| sucrose | TAS1R3 | binding | f |
| sucrose | CYP2B6 | binding | f |
| sucrose | HUS1 | binding | f |
| sucrose | PRODH | inhibition | t |
| sucrose | REN | inhibition | t |
| sucrose | HMOX1 | binding | f |
| sucrose | PMCH | activation | f |
| sucrose | ATOX1 | binding | f |
| sucrose | CYP2E1 | binding | f |
| sucrose | RAD9A | binding | f |
| sucrose | CD4 | binding | f |
| sucrose | GLA | catalysis | f |
| sucrose | CYCS | binding | f |
| sucrose | LTF | binding | f |
| sucrose | FOS | activation | t |
| taxifolin | MTTP | inhibition | t |
| taxifolin | TNFSF11 | inhibition | t |
| taxifolin | APOB | inhibition | t |
| taxifolin | NQO1 | activation | t |
| taxifolin | ABCC1 | inhibition | t |
| trans-resveratrol | CYP1A2 | inhibition | f |
| trans-resveratrol | SRC | inhibition | t |
| trans-resveratrol | PCK1 | activation | f |
| trans-resveratrol | AMD1 | inhibition | t |
| trans-resveratrol | PGR | inhibition | t |
| trans-resveratrol | CS | activation | t |
| trans-resveratrol | NQO2 | catalysis | f |
| trans-resveratrol | ALDH1A1 | inhibition | t |
| trans-resveratrol | ELAVL1 | activation | t |
| trans-resveratrol | PRKCA | inhibition | t |
| trans-resveratrol | SMAD3 | inhibition | t |
| trans-resveratrol | SCNN1A | inhibition | f |
| trans-resveratrol | ALOX12 | inhibition | t |
| trans-resveratrol | NOS1 | inhibition | f |
| trans-resveratrol | HSPA4 | inhibition | t |
| trans-resveratrol | PTEN | activation | t |
| trans-resveratrol | APEX1 | inhibition | t |
| trans-resveratrol | IL8 | inhibition | t |
| trans-resveratrol | STAB2 | activation | t |
| trans-resveratrol | PDE5A | inhibition | t |
| trans-resveratrol | EGR1 | activation | t |
| trans-resveratrol | CAT | inhibition | t |
| trans-resveratrol | CDH1 | activation | t |
| trans-resveratrol | CCND1 | inhibition | t |
| trans-resveratrol | CYP1A1 | inhibition | t |
| trans-resveratrol | NPY | inhibition | t |
| trans-resveratrol | TGFB1 | inhibition | t |
| trans-resveratrol | MAP2K1 | activation | t |
| trans-resveratrol | LTA4H | binding | f |
| trans-resveratrol | AGTR1 | inhibition | t |
| trans-resveratrol | PPARG | binding | f |
| trans-resveratrol | MAPK14 | activation | t |
| trans-resveratrol | NR1I3 | activation | t |
| trans-resveratrol | PTPN1 | inhibition | t |
| trans-resveratrol | CASP6 | activation | t |
| trans-resveratrol | CTNNB1 | activation | t |
| trans-resveratrol | ACE | inhibition | t |
| trans-resveratrol | PTGS2 | binding | t |
| trans-resveratrol | BSG | inhibition | t |
| trans-resveratrol | G6PD | activation | t |
| trans-resveratrol | TYR | inhibition | t |
| trans-resveratrol | PRKAA2 | activation | t |
| trans-resveratrol | ATP5C1 | binding | f |
| trans-resveratrol | SIRT7 | activation | t |
| trans-resveratrol | PRKAA1 | activation | t |
| trans-resveratrol | PTPN11 | activation | t |
| trans-resveratrol | ADIPOQ | activation | t |
| trans-resveratrol | MAPK8 | inhibition | t |
| trans-resveratrol | PIK3CG | inhibition | t |
| trans-resveratrol | GPX1 | activation | t |
| trans-resveratrol | CUL5 | activation | t |
| trans-resveratrol | POMC | inhibition | t |
| trans-resveratrol | ATP5A1 | binding | f |
| trans-resveratrol | KLK3 | inhibition | t |
| trans-resveratrol | CBR4 | activation | t |
| trans-resveratrol | TLR2 | inhibition | t |
| trans-resveratrol | CREB1 | activation | t |
| trans-resveratrol | MAPK3 | inhibition | t |
| trans-resveratrol | PKM2 | inhibition | t |
| trans-resveratrol | BGLAP | activation | f |
| trans-resveratrol | JUN | activation | t |
| trans-resveratrol | IL18 | inhibition | t |
| trans-resveratrol | TBXA2R | inhibition | t |
| trans-resveratrol | PFKM | inhibition | t |
| trans-resveratrol | RELA | inhibition | t |
| trans-resveratrol | FOXO1 | activation | t |
| trans-resveratrol | SULT1B1 | binding | f |
| trans-resveratrol | CASP9 | inhibition | t |
| trans-resveratrol | CFTR | activation | t |
| trans-resveratrol | TRPA1 | inhibition | f |
| trans-resveratrol | CDK2 | inhibition | t |
| trans-resveratrol | ATP5B | binding | f |
| trans-resveratrol | GSK3A | activation | t |
| trans-resveratrol | PRNP | inhibition | t |
| trans-resveratrol | GSK3B | activation | t |
| trans-resveratrol | FASLG | activation | t |
| trans-resveratrol | SULT1E1 | inhibition | t |
| trans-resveratrol | HMOX1 | inhibition | t |
| trans-resveratrol | FOXO3 | activation | t |
| trans-resveratrol | MTOR | inhibition | t |
| trans-resveratrol | PPARA | activation | t |
| trans-resveratrol | SERPINE2 | inhibition | f |
| trans-resveratrol | PRKD1 | inhibition | t |
| trans-resveratrol | NR1I2 | activation | t |
| trans-resveratrol | CSNK2A1 | catalysis | f |
| trans-resveratrol | TNFRSF10B | activation | t |
| trans-resveratrol | EGF | inhibition | t |
| trans-resveratrol | ELANE | inhibition | t |
| trans-resveratrol | HIF1A | inhibition | t |
| trans-resveratrol | ESR1 | binding | f |
| trans-resveratrol | CYP2E1 | inhibition | t |
| trans-resveratrol | NOS3 | activation | t |
| trans-resveratrol | CCR2 | inhibition | f |
| trans-resveratrol | CD4 | inhibition | t |
| trans-resveratrol | SLC2A4 | activation | t |
| trans-resveratrol | SPHK1 | inhibition | t |
| trans-resveratrol | PON1 | activation | t |
| trans-resveratrol | SP1 | activation | t |
| trans-resveratrol | IGF1 | inhibition | t |
| trans-resveratrol | BDNF | activation | t |
| trans-resveratrol | FOS | inhibition | t |
| trans-resveratrol | NFE2L2 | activation | t |
| trans-resveratrol | PARP1 | inhibition | t |
| trans-resveratrol | TP53 | activation | t |
| trans-resveratrol | MAPK1 | inhibition | t |
| trans-resveratrol | CD97 | inhibition | t |
| trans-resveratrol | PTGS1 | inhibition | t |
| trans-resveratrol | DIO2 | inhibition | t |
| trans-resveratrol | SOD2 | activation | t |
| trans-resveratrol | CEL | inhibition | t |
| tryptophan | CHRM4 | binding | t |
| tryptophan | SLC6A19 | catalysis | f |
| tryptophan | IL4I1 | catalysis | f |
| tryptophan | HLA-A | catalysis | f |
| tryptophan | WARS | binding | t |
| tryptophan | SLC7A8 | catalysis | f |
| tryptophan | TDO2 | catalysis | f |
| tryptophan | CRH | inhibition | t |
| tryptophan | WARS2 | catalysis | f |
| tryptophan | DAO | binding | t |
| tryptophan | SLC16A10 | catalysis | f |
| tryptophan | UBC | reaction | f |
| tryptophan | IDO1 | binding | t |
| tryptophan | CHRM1 | binding | t |
| tryptophan | DDC | catalysis | f |
| tryptophan | TPH1 | binding | t |
| tryptophan | TPH2 | catalysis | f |
| tryptophan | SLC3A2 | catalysis | f |
| uridine | NT5E | catalysis | f |
| uridine | LSM2 | binding | f |
| uridine | NT5C1A | catalysis | f |
| uridine | CDA | binding | f |
| uridine | SLC28A2 | catalysis | f |
| uridine | UPP1 | catalysis | f |
| uridine | UCKL1 | catalysis | f |
| uridine | UCK2 | catalysis | f |
| uridine | LSM6 | binding | f |
| uridine | UCK1 | catalysis | f |
| uridine | SLC29A2 | catalysis | f |
| uridine | NT5M | catalysis | f |
| uridine | SLC28A3 | catalysis | f |
| uridine | NT5C3 | catalysis | f |
| uridine | TYMP | inhibition | t |
| uridine | P2RY6 | activation | t |
| uridine | SLC29A3 | catalysis | f |
| uridine | NT5C | catalysis | f |
| uridine | SLC29A1 | catalysis | f |
| uridine | AICDA | catalysis | f |
| uridine | NT5C2 | catalysis | f |
| ursolic acid | TOP1 | inhibition | t |
| ursolic acid | CASP3 | activation | t |
| ursolic acid | TOP2A | inhibition | t |
| ursolic acid | BAK1 | activation | t |
|  |  |  |  |

**Supplement Table S6** Targets of compounds.

A list of 1101 targets of 80 compounds that were collected from STITCH.

| **Compound** | **Target** | **Compound** | **Target** | **Compound** | **Target** |
| --- | --- | --- | --- | --- | --- |
| (-)-epicatechin | NFKB1 | caffeic acid | ELANE | hexanoic acid | AGTR1 |
| (-)-epicatechin | IL6 | caffeic acid | SELP | hexanoic acid | HTR2C |
| (-)-epicatechin | DPEP1 | caffeic acid | SMC2 | hexanoic acid | GNRH1 |
| (-)-epicatechin | CALCOCO1 | caffeic acid | HMGCR | hexanoic acid | NPFFR1 |
| (-)-epicatechin | NOS3 | caffeic acid | ALOX15 | hexanoic acid | OXSM |
| (-)-epicatechin | COMT | caffeic acid | FASN | hexanoic acid | CYSLTR2 |
| (-)-epicatechin | CEL | caffeic acid | ALOXE3 | hexanoic acid | APP |
| 18alpha-glycyrrhetinic acid | GJA1 | caffeic acid | SOD2 | hexanoic acid | AKR1B1 |
| 18beta-glycyrrhetinic acid | NR3C1 | caffeic acid | RAC1 | hexanoic acid | PTGER1 |
| 18beta-glycyrrhetinic acid | HSD11B1 | caffeic acid | CALM1 | hexanoic acid | HCRT |
| 18beta-glycyrrhetinic acid | ICAM1 | caffeic acid | COMT | hexanoic acid | CCKAR |
| 18beta-glycyrrhetinic acid | PTGR2 | caffeic acid | TOP1 | hexanoic acid | PROK2 |
| 18beta-glycyrrhetinic acid | GJA1 | caffeic acid | PTGS2 | hexanoic acid | CASR |
| 18beta-glycyrrhetinic acid | ANPEP | caffeic acid | CGA | hexanoic acid | F2RL2 |
| 18beta-glycyrrhetinic acid | HSD11B2 | caffeic acid | CYP4B1 | hexanoic acid | F2RL1 |
| 18beta-glycyrrhetinic acid | NR3C2 | caffeic acid | ALOX5 | hexanoic acid | AVPR1A |
| 18beta-glycyrrhetinic acid | SRC | caffeic acid | TNF | hexanoic acid | TAC3 |
| 18beta-glycyrrhetinic acid | AKR1B10 | caffeic acid | KCNK2 | hexanoic acid | PTAFR |
| 18beta-glycyrrhetinic acid | MGEA5 | caffeic acid | BDNF | hexanoic acid | TACR3 |
| 2-furaldehyde | HBA1 | canavanine | VIM | hexanoic acid | TRH |
| 2-furaldehyde | HBB | canavanine | ASS1 | hexanoic acid | TACR1 |
| 2-methylbutanoic acid | MMD | canavanine | NOS3 | hexanoic acid | NTSR2 |
| 2-methylbutanoic acid | NAPA | canavanine | CGB | hexanoic acid | PROKR1 |
| 2-methylbutanoic acid | C10orf2 | carvacrol | HSPA4 | hexanoic acid | P2RY1 |
| acetic acid | UTS2 | chlorogenic acid | FDFT1 | hexanoic acid | CHRM1 |
| acetic acid | P2RY10 | chlorogenic acid | TYR | hexanoic acid | ADRA1B |
| acetic acid | ADH7 | chlorogenic acid | HMGCR | hexanoic acid | LTB4R |
| acetic acid | MTHFD1 | chlorogenic acid | BCR | hexanoic acid | BDKRB2 |
| acetic acid | BDKRB1 | chlorogenic acid | FASN | hexanoic acid | NPFFR2 |
| acetic acid | PROKR2 | chlorogenic acid | TCFL5 | hexanoic acid | PLG |
| acetic acid | OXT | chlorogenic acid | CYP3A4 | hexanoic acid | GPRC6A |
| acetic acid | ACOT8 | chlorogenic acid | PPOX | hexanoic acid | P2RY6 |
| acetic acid | MLNR | chlorogenic acid | PTGS2 | hexanoic acid | TRHR |
| acetic acid | DHODH | chlorogenic acid | CGA | hexanoic acid | P2RY2 |
| acetic acid | DECR1 | choline | M6PR | hexanoic acid | XCR1 |
| acetic acid | RETN | choline | SLC22A4 | hexanoic acid | EDNRA |
| acetic acid | PON1 | choline | OXT | hexanoic acid | GPR4 |
| acetic acid | PON2 | choline | CHPT1 | hexanoic acid | TAC1 |
| acetic acid | OGDH | choline | IL4 | hexanoic acid | F2R |
| acetic acid | VIM | choline | IL5 | hexanoic acid | UTS2R |
| acetic acid | MPO | choline | SLC22A5 | hexanoic acid | OXTR |
| acetic acid | PIGL | choline | DMGDH | hexanoic acid | GPR132 |
| acetic acid | ALDH3A1 | choline | PEMT | hexanoic acid | CHRM5 |
| acetic acid | GNRHR | choline | GAD2 | hexanoic acid | GAST |
| acetic acid | PDHX | choline | EPT1 | hexanoic acid | GNG2 |
| acetic acid | LTA4H | choline | CDH1 | hexanoic acid | EDNRB |
| acetic acid | APOB | choline | PLD2 | hexanoic acid | CCKBR |
| acetic acid | ODC1 | choline | SLC5A7 | hexanoic acid | QRFPR |
| acetic acid | KISS1R | choline | LCT | hexanoic acid | CCK |
| acetic acid | TTR | choline | BCHE | hexanoic acid | EDN3 |
| acetic acid | ACYP1 | choline | CHKA | hexanoic acid | CIDEB |
| acetic acid | GPR68 | choline | ETNK1 | hexanoic acid | FPR2 |
| acetic acid | CAT | choline | TP53 | hexanoic acid | UTS2D |
| acetic acid | NAGK | choline | SLC44A3 | hexanoic acid | LPAR6 |
| acetic acid | GNRH2 | choline | REN | hexanoic acid | ACACA |
| acetic acid | DSTN | choline | BHMT | hexanoic acid | QRFP |
| acetic acid | UROD | choline | SLC22A3 | hexanoic acid | LPAR1 |
| acetic acid | FFAR2 | choline | PCYT1A | hexanoic acid | NPSR1 |
| acetic acid | FFAR1 | choline | DCD | hexanoic acid | AVPR1B |
| acetic acid | CSN1S1 | choline | MITF | hexanoic acid | KISS1 |
| acetic acid | F2RL3 | choline | KIAA0101 | hexanoic acid | NTSR1 |
| acetic acid | TST | choline | PRL | hexanoic acid | LPAR3 |
| acetic acid | NARFL | choline | ACHE | hexanoic acid | PTGFR |
| acetic acid | CYP2E1 | choline | CSN3 | hexanoic acid | OPN4 |
| acetic acid | ALDH3B2 | choline | FOS | hexanoic acid | EDN2 |
| acetic acid | CHRM3 | choline | PTDSS2 | hexanoic acid | LPAR4 |
| acetic acid | NTS | choline | PLA2G1B | hexanoic acid | CYSLTR1 |
| acetic acid | GRP | choline | ISYNA1 | hexanoic acid | TACR2 |
| acetic acid | ANXA1 | choline | PLD6 | hexanoic acid | HCRTR1 |
| acetic acid | NMBR | choline | CHDH | hexanoic acid | TBXA2R |
| acetic acid | HTR2B | choline | PCSK7 | hexanoic acid | GNA14 |
| acetic acid | IDH1 | choline | PMCH | hexanoic acid | HTR2A |
| acetic acid | SLC16A7 | choline | SLC44A2 | hexanoic acid | EDN1 |
| acetic acid | SYT1 | choline | CHAT | hexanoic acid | ADRA1D |
| acetic acid | ACSS3 | choline | PTDSS1 | hexanoic acid | GRPR |
| acetic acid | LYZ | choline | IGF2 | hexanoic acid | NPS |
| acetic acid | PEBP1 | choline | SMUG1 | hexanoic acid | GCGR |
| acetic acid | ALDH2 | choline | PLD1 | hexanoic acid | LPAR2 |
| acetic acid | FAH | choline | S100A4 | hexanoic acid | GCG |
| acetic acid | NDST1 | choline | IGBP1 | inositol | MIOX |
| acetic acid | TIMP2 | choline | PLD3 | inositol | CDIPT |
| acetic acid | PAFAH1B3 | choline | RASSF1 | inositol | OPRD1 |
| acetic acid | GNA15 | choline | NTRK1 | inositol | NPY |
| acetic acid | ASPA | choline | SGMS2 | inositol | GDE1 |
| acetic acid | SIAE | choline | SLC22A2 | inositol | PIK3C3 |
| acetic acid | TYRO3 | choline | SLC22A1 | inositol | IMPAD1 |
| acetic acid | SLC9A1 | choline | NGF | inositol | PI4K2B |
| acetic acid | NDST4 | choline | CHRNA4 | inositol | PRKAR2A |
| acetic acid | GRIA2 | choline | SLC44A5 | inositol | CHKA |
| acetic acid | KNG1 | choline | SLC18A3 | inositol | IMPA2 |
| acetic acid | EGF | choline | SLC44A1 | inositol | PI4KB |
| acetic acid | MLN | choline | SUV39H1 | inositol | SLC2A13 |
| acetic acid | NPFF | choline | MYC | inositol | AKR1B1 |
| acetic acid | GPR65 | choline | PCYT1B | inositol | SLC5A11 |
| acetic acid | ABAT | choline | TH | inositol | MITF |
| acetic acid | PROK1 | choline | GJB2 | inositol | PDP1 |
| acetic acid | KCNH1 | choline | PLD4 | inositol | PLK1 |
| acetic acid | REN | choline | CHKB | inositol | PDIA3 |
| acetic acid | GPR17 | choline | ZGLP1 | inositol | GBA |
| acetic acid | AGTR1 | choline | GCG | inositol | ISYNA1 |
| acetic acid | PLA2G7 | choline | SLC44A4 | inositol | INPP1 |
| acetic acid | LAT2 | choline | TNF | inositol | PIK3R4 |
| acetic acid | HTR2C | choline | AGPAT1 | inositol | PIK3CG |
| acetic acid | GNRH1 | choline | BDNF | inositol | SLC6A12 |
| acetic acid | NPFFR1 | choline | C4B | inositol | PI4K2A |
| acetic acid | IL18 | chrysoeriol | CYP1B1 | inositol | SLC5A3 |
| acetic acid | OXSM | chrysoeriol | ATN1 | inositol | IMPA1 |
| acetic acid | CYSLTR2 | chrysoeriol | CYP1A1 | isoeugenol | IL2 |
| acetic acid | HS3ST3A1 | chrysoeriol | ABCC1 | isoquercitrin | CYP1B1 |
| acetic acid | APP | cinnamaldehyde | TRPV1 | isoquercitrin | CYP1A1 |
| acetic acid | CA2 | cinnamaldehyde | MAPK14 | isorhamnetin | PON2 |
| acetic acid | HMGCR | cinnamaldehyde | TRPA1 | isorhamnetin | CYP1B1 |
| acetic acid | MDP1 | cinnamaldehyde | CASP3 | isorhamnetin | EGF |
| acetic acid | CREB3L1 | cinnamaldehyde | TRPM8 | isorhamnetin | ABCB1 |
| acetic acid | SLCO2B1 | cinnamaldehyde | RELA | isorhamnetin | AKT1 |
| acetic acid | MCAT | cinnamaldehyde | TNF | isorhamnetin | CYP1A2 |
| acetic acid | ACE | citronellol | ZNF143 | isorhamnetin | NR1H3 |
| acetic acid | PTGER1 | crocetin | CCND1 | isorhamnetin | CYP1A1 |
| acetic acid | HCRT | crocin | ADRBK1 | jatrorrhizine | SELE |
| acetic acid | CELA1 | crocin | PNLIP | jatrorrhizine | TNF |
| acetic acid | GNPDA2 | crocin | TNF | kaempferol | CD9 |
| acetic acid | CCKAR | d-mannitol | TECR | kaempferol | MAPK1 |
| acetic acid | PROK2 | d-mannitol | MIOX | kaempferol | NFKBIA |
| acetic acid | ALB | d-mannitol | TIMP1 | kaempferol | TNFRSF10A |
| acetic acid | ABHD6 | d-mannitol | AQP9 | kaempferol | CCL2 |
| acetic acid | CASR | d-mannitol | DECR1 | kaempferol | ABCG2 |
| acetic acid | NDST3 | d-mannitol | NFKB1 | kaempferol | TNFSF10 |
| acetic acid | F2RL2 | d-mannitol | LTF | kaempferol | AHR |
| acetic acid | F2RL1 | d-mannitol | CAT | kaempferol | ALOX12 |
| acetic acid | NOS3 | d-mannitol | SLC12A2 | kaempferol | IL6 |
| acetic acid | ALDH1A1 | d-mannitol | SLC9A1 | kaempferol | CYP1B1 |
| acetic acid | AVPR1A | d-mannitol | POMC | kaempferol | ABCB1 |
| acetic acid | CKB | d-mannitol | PIK3C2A | kaempferol | UGT3A1 |
| acetic acid | NDST2 | d-mannitol | SORD | kaempferol | TNFRSF10B |
| acetic acid | TAC3 | d-mannitol | PROK1 | kaempferol | AKR1B1 |
| acetic acid | KLK1 | d-mannitol | PTS | kaempferol | BAX |
| acetic acid | PTAFR | d-mannitol | TJP1 | kaempferol | HPGDS |
| acetic acid | ACHE | d-mannitol | ALB | kaempferol | CSF2 |
| acetic acid | ACOT12 | d-mannitol | IGF1 | kaempferol | TNFRSF11B |
| acetic acid | RNASE2 | d-mannitol | LEP | kaempferol | IL8 |
| acetic acid | TACR3 | d-mannitol | SPHK1 | kaempferol | CASP3 |
| acetic acid | TRH | d-mannitol | SOD2 | kaempferol | PCSK7 |
| acetic acid | TACR1 | d-mannitol | AMY2B | kaempferol | BCL2 |
| acetic acid | NTSR2 | d-mannitol | S100A4 | kaempferol | CASP9 |
| acetic acid | PROKR1 | d-mannitol | LOC100506658 | kaempferol | NR1I2 |
| acetic acid | PAFAH1B2 | d-mannitol | FAS | kaempferol | CYP3A4 |
| acetic acid | P2RY1 | d-mannitol | SRC | kaempferol | CYP1A2 |
| acetic acid | ANTXR2 | d-mannitol | CDKN2A | kaempferol | ESR2 |
| acetic acid | FOS | d-mannitol | PTGS2 | kaempferol | SRC |
| acetic acid | CHRM1 | d-mannitol | AMY1A | kaempferol | MAPK8 |
| acetic acid | ISG20 | d-mannitol | NPPA | kaempferol | STAT1 |
| acetic acid | ADRA1B | d-mannitol | AVP | kaempferol | PTGS2 |
| acetic acid | KIF5B | d-mannitol | AQP4 | kaempferol | CHUK |
| acetic acid | LTB4R | d-mannitol | AMY2A | kaempferol | TXN |
| acetic acid | AMDHD2 | d-mannoheptulose | GCK | kaempferol | CYP1A1 |
| acetic acid | CANT1 | d-mannoheptulose | SHPK | kaempferol | DIO2 |
| acetic acid | BDKRB2 | d-mannoheptulose | MLXIPL | kaempferol | GSTP1 |
| acetic acid | NPFFR2 | d-mannoheptulose | SLC2A2 | kaempferol | ABCC1 |
| acetic acid | GPRC6A | dibutyl phthalate | ART4 | kaempferol | MYH14 |
| acetic acid | P2RY6 | dibutyl phthalate | POLK | kaempferol | SLC2A1 |
| acetic acid | TRHR | dibutyl phthalate | FST | l-galactoheptulose | SHPK |
| acetic acid | P2RY2 | dibutyl phthalate | DMPK | linalool | KCNH1 |
| acetic acid | XCR1 | dibutyl phthalate | MBP | linalool | AMD1 |
| acetic acid | GNPDA1 | dibutyl phthalate | NR1I3 | linamarin | GBA2 |
| acetic acid | UCP2 | dibutyl phthalate | TM2D1 | loganin | TNF |
| acetic acid | PLA2G1B | dihydroquercetin | APOB | lupeol | PLK1 |
| acetic acid | LEP | dihydroquercetin | SPAG9 | lupeol | CDC25C |
| acetic acid | KLK15 | dihydroquercetin | ICAM1 | luteolin | MAPK1 |
| acetic acid | EDNRA | dihydroquercetin | MTTP | luteolin | GSK3A |
| acetic acid | CD55 | dihydroquercetin | NQO1 | luteolin | HGF |
| acetic acid | ACSS1 | dihydroquercetin | ABCC1 | luteolin | NFKB1 |
| acetic acid | GPR4 | dihydroresveratrol | LTA4H | luteolin | IL2 |
| acetic acid | PCK1 | diosgenin | HGF | luteolin | CCND1 |
| acetic acid | NOS1 | diosgenin | IFNG | luteolin | TNFSF11 |
| acetic acid | SLC2A4 | diosgenin | LPL | luteolin | IL6 |
| acetic acid | TAC1 | diosgenin | NR1I2 | luteolin | CYP1B1 |
| acetic acid | F2R | diosgenin | PTPN11 | luteolin | CDH1 |
| acetic acid | SLC16A8 | diosgenin | SRC | luteolin | STAT3 |
| acetic acid | UTS2R | diosgenin | PTGS2 | luteolin | EGF |
| acetic acid | HSPA5 | diosgenin | TNF | luteolin | CDK2 |
| acetic acid | OXTR | dulcitol | GALK1 | luteolin | ERBB2 |
| acetic acid | GPR132 | dulcitol | SORD | luteolin | AKT1 |
| acetic acid | CHRM5 | dulcitol | AKR1B1 | luteolin | EGFR |
| acetic acid | GAST | dulcitol | GALE | luteolin | TNFRSF10B |
| acetic acid | IDH2 | dulcitol | GALT | luteolin | PPARG |
| acetic acid | ALDH1A3 | dulcitol | ARIH1 | luteolin | CSF2 |
| acetic acid | ADSSL1 | ecdysterone | HSPB1 | luteolin | NOS3 |
| acetic acid | GNG2 | ecdysterone | CALCA | luteolin | HSP90B1 |
| acetic acid | EDNRB | ecdysterone | USP1 | luteolin | IGF1 |
| acetic acid | CCKBR | ecdysterone | PRDM1 | luteolin | FOS |
| acetic acid | QRFPR | ecdysterone | ECD | luteolin | IL8 |
| acetic acid | CCK | ecdysterone | TMEM132D | luteolin | OLR1 |
| acetic acid | SOD2 | enoxaparin | SERPIND1 | luteolin | CASP3 |
| acetic acid | EDN3 | enoxaparin | PLAT | luteolin | MET |
| acetic acid | CIDEB | enoxaparin | MPO | luteolin | CTRL |
| acetic acid | CRYZ | enoxaparin | TFPI | luteolin | GSK3B |
| acetic acid | FPR2 | enoxaparin | PROC | luteolin | PCSK7 |
| acetic acid | UTS2D | enoxaparin | GIF | luteolin | TBK1 |
| acetic acid | MAOA | enoxaparin | PIK3C2A | luteolin | CASP9 |
| acetic acid | CYP1A2 | enoxaparin | ALB | luteolin | SMAD3 |
| acetic acid | CS | enoxaparin | PF4 | luteolin | PTK2 |
| acetic acid | EXT2 | enoxaparin | KIAA0101 | luteolin | ESR2 |
| acetic acid | ESR2 | enoxaparin | F2 | luteolin | ATN1 |
| acetic acid | RNASE1 | enoxaparin | NEFH | luteolin | MAPK8 |
| acetic acid | LPAR6 | enoxaparin | F3 | luteolin | TOP1 |
| acetic acid | ACACA | enoxaparin | ACACA | luteolin | PTGS2 |
| acetic acid | QRFP | enoxaparin | SERPINC1 | luteolin | JUN |
| acetic acid | ALDH3A2 | enoxaparin | F5 | luteolin | VEGFA |
| acetic acid | ALDH9A1 | enoxaparin | TKTL1 | luteolin | MMP9 |
| acetic acid | CALM1 | enoxaparin | MMP9 | luteolin | PCNA |
| acetic acid | GM2A | enoxaparin | F10 | luteolin | EDN1 |
| acetic acid | LPAR1 | enoxaparin | PROS1 | luteolin | CYP1A1 |
| acetic acid | HAND2 | enoxaparin | TNF | luteolin | JAK2 |
| acetic acid | MB | epicatechin | NFKB1 | luteolin | AKT2 |
| acetic acid | NPSR1 | epicatechin | IL6 | luteolin | ABCC1 |
| acetic acid | ACSS2 | epicatechin | DPEP1 | luteolin | TNF |
| acetic acid | PRSS3 | epicatechin | CALCOCO1 | magnoflorine | ADIPOQ |
| acetic acid | FH | epicatechin | NOS3 | obaculactone | TBX21 |
| acetic acid | AVPR1B | epicatechin | COMT | palmatine | TNF |
| acetic acid | KISS1 | epicatechin | CEL | pentanoic acid | UTS2 |
| acetic acid | SLC16A1 | eugenol | KCNA5 | pentanoic acid | P2RY10 |
| acetic acid | NTSR1 | eugenol | MAOA | pentanoic acid | BDKRB1 |
| acetic acid | LPAR3 | eugenol | PTGS2 | pentanoic acid | PROKR2 |
| acetic acid | PTGFR | eugenol | ALOX5 | pentanoic acid | OXT |
| acetic acid | RAE1 | eugenol | GLI3 | pentanoic acid | MLNR |
| acetic acid | PTPN1 | eugenol | BDNF | pentanoic acid | GNRHR |
| acetic acid | OPN4 | farnesol | FDFT1 | pentanoic acid | KISS1R |
| acetic acid | EDN2 | farnesol | TLR2 | pentanoic acid | GPR68 |
| acetic acid | GNMT | farnesol | PPARA | pentanoic acid | GNRH2 |
| acetic acid | LPAR4 | farnesol | SRD5A3 | pentanoic acid | FFAR2 |
| acetic acid | CYSLTR1 | farnesol | HMGCR | pentanoic acid | FFAR1 |
| acetic acid | TACR2 | farnesol | UGT2B7 | pentanoic acid | F2RL3 |
| acetic acid | HCRTR1 | farnesol | HMGA1 | pentanoic acid | CHRM3 |
| acetic acid | PAFAH2 | farnesol | CASP3 | pentanoic acid | NTS |
| acetic acid | PLA2G2E | farnesol | MAOB | pentanoic acid | GRP |
| acetic acid | TBXA2R | ferulic acid | MAPK1 | pentanoic acid | ANXA1 |
| acetic acid | CLYBL | ferulic acid | FANCE | pentanoic acid | NMBR |
| acetic acid | GNA14 | ferulic acid | TPSG1 | pentanoic acid | HTR2B |
| acetic acid | ALDH1B1 | ferulic acid | CSN1S1 | pentanoic acid | GNA15 |
| acetic acid | MAOB | ferulic acid | ADIPOQ | pentanoic acid | KNG1 |
| acetic acid | EXT1 | ferulic acid | CYP1A2 | pentanoic acid | GAL |
| acetic acid | HTR2A | ferulic acid | PTGS2 | pentanoic acid | MLN |
| acetic acid | ESD | ferulic acid | VEGFA | pentanoic acid | NPFF |
| acetic acid | EDN1 | ferulic acid | AR | pentanoic acid | GPR65 |
| acetic acid | ADRA1D | ferulic acid | CYP1A1 | pentanoic acid | PROK1 |
| acetic acid | IL2RA | folinic acid | CSF3 | pentanoic acid | GPR17 |
| acetic acid | GRPR | folinic acid | PFN2 | pentanoic acid | AGTR1 |
| acetic acid | AKR1C3 | folinic acid | MTHFS | pentanoic acid | HTR2C |
| acetic acid | TNK2 | folinic acid | IFNA1 | pentanoic acid | GNRH1 |
| acetic acid | TYRP1 | folinic acid | QDPR | pentanoic acid | NPFFR1 |
| acetic acid | G6PD | folinic acid | STK36 | pentanoic acid | CYSLTR2 |
| acetic acid | PC | folinic acid | CSF2 | pentanoic acid | APP |
| acetic acid | CCT4 | folinic acid | SLC19A1 | pentanoic acid | PTGER1 |
| acetic acid | ACYP2 | folinic acid | TYMS | pentanoic acid | HCRT |
| acetic acid | TPI1 | folinic acid | DHFRL1 | pentanoic acid | CCKAR |
| acetic acid | CARD11 | folinic acid | RPL22 | pentanoic acid | PROK2 |
| acetic acid | PAFAH1B1 | folinic acid | SPTBN1 | pentanoic acid | CASR |
| acetic acid | HAGH | folinic acid | TOP1 | pentanoic acid | F2RL2 |
| acetic acid | NPS | folinic acid | DPYD | pentanoic acid | F2RL1 |
| acetic acid | DOT1L | folinic acid | FPGS | pentanoic acid | AVPR1A |
| acetic acid | GCGR | folinic acid | MTHFR | pentanoic acid | TAC3 |
| acetic acid | LPAR2 | folinic acid | IFNA2 | pentanoic acid | PTAFR |
| acetic acid | ALDH7A1 | folinic acid | DHFR | pentanoic acid | TACR3 |
| acetic acid | GCG | folinic acid | IKZF3 | pentanoic acid | TRH |
| acetic acid | TNF | formononetin | SIRT1 | pentanoic acid | TACR1 |
| acetic acid | HMGXB3 | formononetin | CYP1B1 | pentanoic acid | NTSR2 |
| acetic acid | LDHA | formononetin | JUN | pentanoic acid | PROKR1 |
| acetic acid | C2 | formononetin | VEGFA | pentanoic acid | P2RY1 |
| acetic acid | IL10 | formononetin | BMP2 | pentanoic acid | CHRM1 |
| aconitine | KCNA5 | formononetin | CYP1A1 | pentanoic acid | ADRA1B |
| aconitine | KCNH2 | geniposide | HMOX1 | pentanoic acid | LTB4R |
| aconitine | COLQ | geniposide | PRDM2 | pentanoic acid | BDKRB2 |
| adenine | PYGM | geniposide | IL6 | pentanoic acid | NPFFR2 |
| adenine | SIRT1 | geniposide | IL8 | pentanoic acid | GPRC6A |
| adenine | ADSL | geraniol | CYP2B6 | pentanoic acid | P2RY6 |
| adenine | AHCY | glycyrrhetinic acid | NR3C1 | pentanoic acid | TRHR |
| adenine | MPG | glycyrrhetinic acid | HSD11B1 | pentanoic acid | P2RY2 |
| adenine | DECR1 | glycyrrhetinic acid | ICAM1 | pentanoic acid | XCR1 |
| adenine | FCGRT | glycyrrhetinic acid | PTGR2 | pentanoic acid | EDNRA |
| adenine | PPIF | glycyrrhetinic acid | GJA1 | pentanoic acid | GPR4 |
| adenine | CPD | glycyrrhetinic acid | ANPEP | pentanoic acid | TAC1 |
| adenine | GAPDH | glycyrrhetinic acid | HSD11B2 | pentanoic acid | F2R |
| adenine | ATIC | glycyrrhetinic acid | NR3C2 | pentanoic acid | UTS2R |
| adenine | KCNJ8 | glycyrrhetinic acid | SRC | pentanoic acid | OXTR |
| adenine | NT5E | glycyrrhetinic acid | AKR1B10 | pentanoic acid | GPR132 |
| adenine | UCP1 | glycyrrhetinic acid | MGEA5 | pentanoic acid | CHRM5 |
| adenine | PPAT | glycyrrhizin | MMP2 | pentanoic acid | GAST |
| adenine | NNT | glycyrrhizin | CCL2 | pentanoic acid | GNG2 |
| adenine | PECR | glycyrrhizin | GOT2 | pentanoic acid | EDNRB |
| adenine | PRKAR2A | glycyrrhizin | IL6 | pentanoic acid | CCKBR |
| adenine | PNMT | glycyrrhizin | REN | pentanoic acid | QRFPR |
| adenine | PDE4A | glycyrrhizin | IL18 | pentanoic acid | CCK |
| adenine | ACP1 | glycyrrhizin | CCL5 | pentanoic acid | EDN3 |
| adenine | GLUD1 | glycyrrhizin | JUNB | pentanoic acid | CIDEB |
| adenine | SLC25A4 | glycyrrhizin | CXCL10 | pentanoic acid | FPR2 |
| adenine | PTH | glycyrrhizin | IL8 | pentanoic acid | UTS2D |
| adenine | SPOCK1 | glycyrrhizin | NAT1 | pentanoic acid | LPAR6 |
| adenine | ADK | glycyrrhizin | HSD11B2 | pentanoic acid | QRFP |
| adenine | ATP5O | glycyrrhizin | ACPP | pentanoic acid | LPAR1 |
| adenine | HPRT1 | glycyrrhizin | CYP11B2 | pentanoic acid | NPSR1 |
| adenine | METTL3 | glycyrrhizin | CYP3A4 | pentanoic acid | AVPR1B |
| adenine | P2RY1 | glycyrrhizin | NR3C2 | pentanoic acid | KISS1 |
| adenine | OGG1 | glycyrrhizin | MGEA5 | pentanoic acid | NTSR1 |
| adenine | P2RY2 | glycyrrhizin | MMP9 | pentanoic acid | LPAR3 |
| adenine | PRTFDC1 | heparin | DCN | pentanoic acid | PTGFR |
| adenine | PKM2 | heparin | FGF4 | pentanoic acid | OPN4 |
| adenine | CHFR | heparin | SERPIND1 | pentanoic acid | EDN2 |
| adenine | IMPDH2 | heparin | CTSG | pentanoic acid | LPAR4 |
| adenine | GLUD2 | heparin | CSNK2A1 | pentanoic acid | CYSLTR1 |
| adenine | DNMT3B | heparin | F9 | pentanoic acid | TACR2 |
| adenine | BRAP | heparin | TIMP1 | pentanoic acid | HCRTR1 |
| adenine | IDH2 | heparin | PLAT | pentanoic acid | TBXA2R |
| adenine | PKLR | heparin | HAMP | pentanoic acid | GNA14 |
| adenine | ACVR2B | heparin | GSK3A | pentanoic acid | HTR2A |
| adenine | CSN2 | heparin | HGF | pentanoic acid | EDN1 |
| adenine | IMPDH1 | heparin | TFPI2 | pentanoic acid | ADRA1D |
| adenine | DDX21 | heparin | SERPINE1 | pentanoic acid | GRPR |
| adenine | AK2 | heparin | MPO | pentanoic acid | NPS |
| adenine | ENTPD2 | heparin | COL1A1 | pentanoic acid | GCGR |
| adenine | LIG4 | heparin | VTN | pentanoic acid | LPAR2 |
| adenine | SLC29A2 | heparin | DAO | pentanoic acid | ALAD |
| adenine | DNAH8 | heparin | CDKN1B | pentanoic acid | GCG |
| adenine | EMP2 | heparin | IFNG | phenylacetaldehyde | ALDH3A1 |
| adenine | RYR1 | heparin | HBEGF | phenylacetaldehyde | AOC2 |
| adenine | PFKM | heparin | ST8SIA4 | phenylacetaldehyde | ALDH3B2 |
| adenine | PNP | heparin | NR3C1 | phenylacetaldehyde | ALDH2 |
| adenine | ADSS | heparin | LTF | phenylacetaldehyde | AOC3 |
| adenine | TFB1M | heparin | HRG | phenylacetaldehyde | ALDH1A3 |
| adenine | PTGS2 | heparin | TFPI | phenylacetaldehyde | MAOA |
| adenine | XPNPEP1 | heparin | APOB | phenylacetaldehyde | ALDH3A2 |
| adenine | ENTPD1 | heparin | IGFBP5 | phenylacetaldehyde | AOX1 |
| adenine | PRKAA2 | heparin | AZU1 | phenylacetaldehyde | MAOB |
| adenine | SLC25A5 | heparin | PROC | quercetin | CFTR |
| adenine | ADA | heparin | ODC1 | quercetin | CD9 |
| adenine | AK1 | heparin | TPSG1 | quercetin | DRD4 |
| adenine | SLC29A3 | heparin | TTR | quercetin | ESR1 |
| adenine | ADO | heparin | DUSP1 | quercetin | SIRT1 |
| adenine | NPPA | heparin | SRGN | quercetin | MAPK1 |
| adenine | STK24 | heparin | LRP1 | quercetin | XBP1 |
| adenine | TBC1D8 | heparin | EREG | quercetin | HMOX1 |
| adenine | ACACB | heparin | SLURP1 | quercetin | NFKBIA |
| adenine | APRT | heparin | CSN1S1 | quercetin | DECR1 |
| adenine | XDH | heparin | CMA1 | quercetin | PLAT |
| adenine | PRNP | heparin | ELN | quercetin | GSR |
| adenine | MTAP | heparin | APOE | quercetin | SLC5A5 |
| adenine | AK3 | heparin | APOC2 | quercetin | GSK3A |
| adenine | G6PD | heparin | F12 | quercetin | PON1 |
| adenine | SRPK2 | heparin | SDC1 | quercetin | PON2 |
| adenine | PAICS | heparin | APCS | quercetin | SERPINE1 |
| adenine | PARN | heparin | GIF | quercetin | CCL2 |
| adenine | GMPR2 | heparin | NT5E | quercetin | SULT1E1 |
| allantoin | ALLC | heparin | IL6 | quercetin | IL2 |
| allantoin | SLC2A4 | heparin | FGFBP1 | quercetin | HSPA8 |
| allantoin | XDH | heparin | SULF1 | quercetin | SCNN1A |
| astilbin | MMP2 | heparin | MMP13 | quercetin | EIF2AK2 |
| astilbin | MPO | heparin | THBS1 | quercetin | APOB |
| astilbin | MMP9 | heparin | VWF | quercetin | ODC1 |
| astragaloside i | NFKB1 | heparin | NDST1 | quercetin | PRDM2 |
| astragaloside iv | MMP2 | heparin | SERPINA10 | quercetin | ABCG2 |
| astragaloside iv | TNNI3 | heparin | ARFGEF1 | quercetin | TNFSF11 |
| astragaloside iv | PLN | heparin | ITGA2B | quercetin | EGR1 |
| astramembrannin i | MMP2 | heparin | IL1B | quercetin | KLRC4-KLRK1 |
| astramembrannin i | TNNI3 | heparin | ELANE | quercetin | CAT |
| astramembrannin i | PLN | heparin | STAM2 | quercetin | TNFSF10 |
| atractylenolide i | TNF | heparin | GPC1 | quercetin | AHR |
| benzaldehyde | BCL3 | heparin | AREG | quercetin | CD97 |
| benzaldehyde | ALDH3A1 | heparin | FGF2 | quercetin | CSN1S1 |
| benzaldehyde | NFKB1 | heparin | IL11 | quercetin | HSPB1 |
| benzaldehyde | CCND1 | heparin | FGF10 | quercetin | ALOX12 |
| benzaldehyde | LOC100293888 | heparin | KLKB1 | quercetin | GDF15 |
| benzaldehyde | CAPNS1 | heparin | FGFRL1 | quercetin | EIF2S1 |
| benzaldehyde | DLGAP5 | heparin | TNC | quercetin | CCNB1 |
| benzaldehyde | HBXIP | heparin | ENPEP | quercetin | NT5E |
| benzaldehyde | TANK | heparin | EGF | quercetin | IL6 |
| benzaldehyde | ALDH2 | heparin | NRP1 | quercetin | CCL21 |
| benzaldehyde | CDH1 | heparin | PRKAR2A | quercetin | MMP13 |
| benzaldehyde | XRCC1 | heparin | CHKA | quercetin | CYP19A1 |
| benzaldehyde | EPCAM | heparin | FGF7 | quercetin | CYP1B1 |
| benzaldehyde | DNMT3A | heparin | FURIN | quercetin | PPIG |
| benzaldehyde | TP53 | heparin | TP53 | quercetin | SLC16A7 |
| benzaldehyde | ERCC3 | heparin | REN | quercetin | LIAS |
| benzaldehyde | LOC100290337 | heparin | EGFR | quercetin | RPS6KA5 |
| benzaldehyde | MAT2A | heparin | CD44 | quercetin | ATP5B |
| benzaldehyde | FOS | heparin | SERPING1 | quercetin | SMAD7 |
| benzaldehyde | MCM7 | heparin | RNF111 | quercetin | PPARA |
| benzaldehyde | MMP14 | heparin | FGFR4 | quercetin | TYR |
| benzaldehyde | CASP3 | heparin | AQP5 | quercetin | IL1B |
| benzaldehyde | MMP1 | heparin | CELA1 | quercetin | STK17B |
| benzaldehyde | IL32 | heparin | RPL29 | quercetin | ICAM1 |
| benzaldehyde | OR1D2 | heparin | TGFA | quercetin | EGF |
| benzaldehyde | TBK1 | heparin | ALB | quercetin | SIL1 |
| benzaldehyde | MTA1 | heparin | ABHD6 | quercetin | ABCB1 |
| benzaldehyde | HIF1A | heparin | PPBP | quercetin | CDK2 |
| benzaldehyde | PTK2 | heparin | PF4 | quercetin | ERBB3 |
| benzaldehyde | SREBF1 | heparin | MASP1 | quercetin | RB1 |
| benzaldehyde | DNMT1 | heparin | CSF2 | quercetin | HDC |
| benzaldehyde | PTGS2 | heparin | COL1A2 | quercetin | TP53 |
| benzaldehyde | FASLG | heparin | NOS3 | quercetin | ERBB2 |
| benzaldehyde | PTEN | heparin | LIPC | quercetin | AKT1 |
| benzaldehyde | MAT1A | heparin | NDST2 | quercetin | UGT3A1 |
| benzaldehyde | MMP9 | heparin | MMP3 | quercetin | EGFR |
| benzaldehyde | KLF4 | heparin | KLK1 | quercetin | SLC25A4 |
| benzaldehyde | AR | heparin | PRL | quercetin | ATP5A1 |
| benzaldehyde | AOX1 | heparin | PPIC | quercetin | AKR1B1 |
| benzaldehyde | MYC | heparin | CTRB2 | quercetin | TXNDC12 |
| benzaldehyde | CA9 | heparin | CSN3 | quercetin | SLCO2B1 |
| benzaldehyde | RAD17 | heparin | NUDT6 | quercetin | AIRE |
| benzaldehyde | SMYD3 | heparin | FOS | quercetin | BAX |
| benzaldehyde | FUT2 | heparin | IL8 | quercetin | HPGDS |
| benzaldehyde | RHOA | heparin | SDC2 | quercetin | TGFA |
| benzaldehyde | POU5F1 | heparin | HPSE | quercetin | CSF2 |
| benzyl alcohol | ADH4 | heparin | F2 | quercetin | NOS3 |
| benzyl alcohol | AKR1B1 | heparin | PLG | quercetin | CASP7 |
| benzyl alcohol | GCG | heparin | LPL | quercetin | HSPA4 |
| benzyl alcohol | CREB1 | heparin | MLC1 | quercetin | FUT3 |
| benzyl benzoate | BOC | heparin | FGF5 | quercetin | FOS |
| berberine | APEX1 | heparin | BTC | quercetin | OLR1 |
| berberine | NTHL1 | heparin | PLA2G1B | quercetin | CASP3 |
| berberine | MPG | heparin | DAG1 | quercetin | PLA2G1B |
| berberine | PON1 | heparin | PRKDC | quercetin | ALOXE3 |
| berberine | VIM | heparin | SGSH | quercetin | NQO1 |
| berberine | CD69 | heparin | SFTPC | quercetin | ADIPOQ |
| berberine | TNFSF11 | heparin | NCAM1 | quercetin | SULT1A1 |
| berberine | AHR | heparin | BACE1 | quercetin | MMP1 |
| berberine | UNG | heparin | LPA | quercetin | SLC2A2 |
| berberine | LIPE | heparin | SERPINF2 | quercetin | GSK3B |
| berberine | LDLR | heparin | MMP1 | quercetin | TSPO |
| berberine | GDF15 | heparin | A2M | quercetin | SP1 |
| berberine | NR1H2 | heparin | SAV1 | quercetin | BCL2 |
| berberine | IL6 | heparin | GSK3B | quercetin | TBK1 |
| berberine | MSR1 | heparin | PCSK7 | quercetin | CASP9 |
| berberine | KCNH2 | heparin | BGN | quercetin | ABCC5 |
| berberine | PPARA | heparin | P4HB | quercetin | F3 |
| berberine | XRCC1 | heparin | FAM123B | quercetin | HSP90AA1 |
| berberine | PHYH | heparin | ZNF85 | quercetin | NR1I2 |
| berberine | LIG1 | heparin | SELE | quercetin | SOD2 |
| berberine | EPCAM | heparin | HIST2H2AC | quercetin | CYP3A4 |
| berberine | TNFRSF8 | heparin | PRKD1 | quercetin | HIF1A |
| berberine | POLB | heparin | F3 | quercetin | IKBKB |
| berberine | ETFA | heparin | GRK1 | quercetin | CAV1 |
| berberine | TP53 | heparin | FGF1 | quercetin | PLAUR |
| berberine | PLIN2 | heparin | IDS | quercetin | KAT5 |
| berberine | UCHL1 | heparin | PAX4 | quercetin | NPR2 |
| berberine | NEIL2 | heparin | MAPT | quercetin | PTK2 |
| berberine | AKR1B1 | heparin | PTN | quercetin | CYP1A2 |
| berberine | NOS3 | heparin | SLPI | quercetin | MAP3K14 |
| berberine | PTGER4 | heparin | ERBB4 | quercetin | CLDN4 |
| berberine | INSR | heparin | SDC3 | quercetin | HMGB1 |
| berberine | FEN1 | heparin | KRIT1 | quercetin | ESR2 |
| berberine | OGG1 | heparin | SFTPB | quercetin | CTNNB1 |
| berberine | NAT1 | heparin | FN1 | quercetin | UBC |
| berberine | CALB2 | heparin | PRODH | quercetin | QPCT |
| berberine | OLR1 | heparin | TNNT2 | quercetin | FN1 |
| berberine | CASP3 | heparin | BCS1L | quercetin | ATP5C1 |
| berberine | ADIPOQ | heparin | COL18A1 | quercetin | SRC |
| berberine | SLC2A4 | heparin | MDK | quercetin | PIK3CG |
| berberine | MMP1 | heparin | SULF2 | quercetin | DNAH8 |
| berberine | STK11 | heparin | F8 | quercetin | RUNX2 |
| berberine | BCL2 | heparin | CFHR3 | quercetin | HIBCH |
| berberine | CASP9 | heparin | CFH | quercetin | MAPK8 |
| berberine | SELE | heparin | SERPINC1 | quercetin | STAT1 |
| berberine | BSG | heparin | F5 | quercetin | COMT |
| berberine | SOD2 | heparin | SGK1 | quercetin | MTOR |
| berberine | DDIT3 | heparin | HDGF | quercetin | PTGS1 |
| berberine | ATF3 | heparin | S100A9 | quercetin | PARP1 |
| berberine | GATA2 | heparin | JUN | quercetin | PTGS2 |
| berberine | FN1 | heparin | PTGDS | quercetin | FASLG |
| berberine | NEIL1 | heparin | COL5A1 | quercetin | NR1I3 |
| berberine | ERCC5 | heparin | RALGDS | quercetin | HSF2 |
| berberine | SREBF1 | heparin | VEGFA | quercetin | SLC16A1 |
| berberine | LIG4 | heparin | CEL | quercetin | CHUK |
| berberine | FUT4 | heparin | MMP9 | quercetin | ABCC2 |
| berberine | TOP1 | heparin | SDC4 | quercetin | GADD45A |
| berberine | PARP1 | heparin | PLAU | quercetin | JUN |
| berberine | PTPRC | heparin | GSN | quercetin | PRKAA2 |
| berberine | PLA2G4A | heparin | ITPR3 | quercetin | CYP2C8 |
| berberine | PRKAA2 | heparin | CXCL12 | quercetin | VEGFA |
| berberine | PTPN1 | heparin | AR | quercetin | MMP9 |
| berberine | VEGFA | heparin | HSPG2 | quercetin | PLAU |
| berberine | MMP9 | heparin | F10 | quercetin | PIM1 |
| berberine | PLAU | heparin | F7 | quercetin | AR |
| berberine | CXCL12 | heparin | CKS2 | quercetin | HCK |
| berberine | APEX2 | heparin | CTRC | quercetin | ABCC4 |
| berberine | THBD | heparin | THBD | quercetin | MYC |
| berberine | LIG3 | heparin | MYC | quercetin | PCNA |
| berberine | PCNA | heparin | AGRN | quercetin | CYP1A1 |
| berberine | CYP1A1 | heparin | EDN1 | quercetin | IL2RA |
| berberine | ERCC2 | heparin | AVP | quercetin | AKR1C3 |
| berberine | TDG | heparin | HBD | quercetin | NKX3-1 |
| berberine | GCG | heparin | SERPINB1 | quercetin | HR |
| berberine | TNF | heparin | IGFBP3 | quercetin | JAK2 |
| berberine | SLC2A1 | heparin | TH | quercetin | ABCC8 |
| beta-eudesmol | CHRNA4 | heparin | ITPR2 | quercetin | NFE2L2 |
| beta-pinene | SAGE1 | heparin | SOD3 | quercetin | GSTP1 |
| beta-sitosterol | APOE | heparin | STAB2 | quercetin | ABCC1 |
| beta-sitosterol | CYP27A1 | heparin | EPHB6 | quercetin | SETD2 |
| beta-sitosterol | ABCG5 | heparin | PROS1 | quercetin | GCG |
| beta-sitosterol | ABCG8 | heparin | CFI | quercetin | TNF |
| beta-sitosterol | NPC1L1 | heparin | FGFR1 | quercetin | SHC1 |
| beta-sitosterol | CASP3 | heparin | DSPP | quercetin | GBE1 |
| beta-sitosterol | PARP1 | heparin | MASP2 | quercetin | SLC2A1 |
| beta-sitosterol | ABCA1 | heparin | TFDP3 | resveratrol | TNS1 |
| betaine | NFKB1 | heparin | CXCR4 | resveratrol | PPP1R13B |
| betaine | IFNG | heparin | TNF | resveratrol | ESR1 |
| betaine | APOB | heparin | CYR61 | resveratrol | SIRT1 |
| betaine | DMGDH | heparin | FGFR2 | resveratrol | MAPK1 |
| betaine | BHMT2 | heparin | SERPINE2 | resveratrol | XBP1 |
| betaine | PEMT | hexanoic acid | UTS2 | resveratrol | HMOX1 |
| betaine | MTRR | hexanoic acid | P2RY10 | resveratrol | APEX1 |
| betaine | BHMT | hexanoic acid | BDKRB1 | resveratrol | NFKBIA |
| betaine | AKR1B1 | hexanoic acid | PROKR2 | resveratrol | CSNK2A1 |
| betaine | SLC6A1 | hexanoic acid | OXT | resveratrol | FAM48A |
| betaine | ABR | hexanoic acid | MLNR | resveratrol | MMP2 |
| betaine | LPL | hexanoic acid | GNRHR | resveratrol | GSR |
| betaine | CHDH | hexanoic acid | KISS1R | resveratrol | CYP4F2 |
| betaine | SLC6A13 | hexanoic acid | GPR68 | resveratrol | TGFB1 |
| betaine | CBS | hexanoic acid | GNRH2 | resveratrol | GSK3A |
| betaine | SLC6A12 | hexanoic acid | FFAR2 | resveratrol | PON1 |
| betaine | SLC6A6 | hexanoic acid | FFAR1 | resveratrol | RPS6KB1 |
| betaine | MTR | hexanoic acid | F2RL3 | resveratrol | SULT1E1 |
| betaine | DLL1 | hexanoic acid | CHRM3 | resveratrol | NFKB1 |
| betaine | SARDH | hexanoic acid | NTS | resveratrol | CCND1 |
| betaine | GNMT | hexanoic acid | GRP | resveratrol | LTA4H |
| betaine | TLR4 | hexanoic acid | ANXA1 | resveratrol | IFNG |
| betaine | MTHFR | hexanoic acid | NMBR | resveratrol | MAPK14 |
| betaine | SLC5A3 | hexanoic acid | HTR2B | resveratrol | BCL6 |
| betaine | ALDH7A1 | hexanoic acid | GNA15 | resveratrol | APOB |
| caffeic acid | MIF | hexanoic acid | KNG1 | resveratrol | PRDM2 |
| caffeic acid | TYR | hexanoic acid | MLN | resveratrol | CTSD |
| resveratrol | CD97 | hexanoic acid | NPFF | resveratrol | TTR |
| resveratrol | VASP | hexanoic acid | GPR65 | resveratrol | TNFSF11 |
| resveratrol | KLF2 | hexanoic acid | PROK1 | resveratrol | EGR1 |
| resveratrol | SIRT2 | hexanoic acid | GPR17 | resveratrol | CAT |
| resveratrol | ALOX12 | stigmasterol | ABCG5 | resveratrol | TNFSF10 |
| resveratrol | GDF15 | stigmasterol | ABCG8 | resveratrol | AHR |
| resveratrol | CCNA1 | stigmasterol | BCL2 | resveratrol | NPY |
| resveratrol | EIF2S1 | sucrose | CD4 | ursolic acid | NFKBIA |
| resveratrol | IL6 | sucrose | ERCC1 | ursolic acid | IL2 |
| resveratrol | TLR2 | sucrose | APOH | ursolic acid | NR3C1 |
| resveratrol | MMP13 | sucrose | ESR1 | ursolic acid | PTPN3 |
| resveratrol | CYP19A1 | sucrose | OXT | ursolic acid | STAT3 |
| resveratrol | CYP1B1 | sucrose | GLA | ursolic acid | ABCB1 |
| resveratrol | PDGFRB | sucrose | MPG | ursolic acid | SORD |
| resveratrol | MAPK3 | sucrose | DECR1 | ursolic acid | MMP3 |
| resveratrol | IL1B | sucrose | RNASEH2A | ursolic acid | ACHE |
| resveratrol | ELANE | sucrose | NR3C1 | ursolic acid | IL8 |
| resveratrol | STAT3 | sucrose | APOB | ursolic acid | CASP3 |
| resveratrol | ICAM1 | sucrose | TTR | ursolic acid | MMP1 |
| resveratrol | CASP6 | sucrose | CAT | ursolic acid | BCL2 |
| resveratrol | EGF | sucrose | AHR | ursolic acid | TOP1 |
| resveratrol | CDK2 | sucrose | CLTA | ursolic acid | PARP1 |
| resveratrol | RB1 | sucrose | FNTB | ursolic acid | RELA |
| resveratrol | TP53 | sucrose | CSN1S1 | ursolic acid | TOP2A |
| resveratrol | ERBB2 | sucrose | NT5E | trans-resveratrol | PDGFRB |
| resveratrol | SOD1 | sucrose | COX5B | trans-resveratrol | MAPK3 |
| resveratrol | AKT1 | sucrose | HUS1 | trans-resveratrol | IL1B |
| resveratrol | CORIN | sucrose | KHK | trans-resveratrol | ELANE |
| resveratrol | CCNA2 | sucrose | LPO | trans-resveratrol | STAT3 |
| resveratrol | PHIP | sucrose | TREH | trans-resveratrol | ICAM1 |
| resveratrol | EGFR | sucrose | LCT | trans-resveratrol | CASP6 |
| resveratrol | IL18 | sucrose | POMC | trans-resveratrol | EGF |
| resveratrol | ATP5A1 | sucrose | HPX | trans-resveratrol | CDK2 |
| resveratrol | PRKCA | sucrose | TXNL4B | trans-resveratrol | RB1 |
| resveratrol | APP | sucrose | CLTC | trans-resveratrol | TP53 |
| resveratrol | OXA1L | sucrose | NLRP1 | trans-resveratrol | ERBB2 |
| resveratrol | PPARG | sucrose | REN | trans-resveratrol | SOD1 |
| resveratrol | ACE | sucrose | GALM | trans-resveratrol | AKT1 |
| resveratrol | AGRP | sucrose | CRH | trans-resveratrol | CORIN |
| resveratrol | CCR2 | sucrose | PTS | trans-resveratrol | CCNA2 |
| resveratrol | BAX | sucrose | PTH | trans-resveratrol | PHIP |
| resveratrol | MITF | sucrose | GJA1 | trans-resveratrol | EGFR |
| resveratrol | SLC45A2 | sucrose | ACSM3 | trans-resveratrol | IL18 |
| resveratrol | CSF2 | sucrose | ALB | trans-resveratrol | ATP5A1 |
| resveratrol | NOS3 | sucrose | SLC13A4 | trans-resveratrol | PRKCA |
| resveratrol | IFI27 | sucrose | GUSB | trans-resveratrol | APP |
| resveratrol | CUL5 | sucrose | ACHE | trans-resveratrol | OXA1L |
| resveratrol | MMP3 | sucrose | FNTA | trans-resveratrol | PPARG |
| resveratrol | MAP2K1 | sucrose | TRH | trans-resveratrol | ACE |
| resveratrol | BCL2L1 | sucrose | GAA | trans-resveratrol | AGRP |
| resveratrol | HSPA4 | sucrose | FOS | trans-resveratrol | CCR2 |
| resveratrol | CBR4 | sucrose | BDKRB2 | trans-resveratrol | BAX |
| resveratrol | FOS | sucrose | CLTB | trans-resveratrol | MITF |
| resveratrol | IL8 | sucrose | RAD9A | trans-resveratrol | SLC45A2 |
| resveratrol | CYCS | sucrose | AIFM2 | trans-resveratrol | CSF2 |
| resveratrol | GLUL | sucrose | LEP | trans-resveratrol | NOS3 |
| resveratrol | SULT1B1 | sucrose | RNF4 | trans-resveratrol | IFI27 |
| resveratrol | OLR1 | sucrose | ATOX1 | trans-resveratrol | CUL5 |
| resveratrol | LPL | sucrose | ACPP | trans-resveratrol | MMP3 |
| resveratrol | CASP3 | sucrose | FLII | trans-resveratrol | MAP2K1 |
| resveratrol | UCP2 | sucrose | YES1 | trans-resveratrol | BCL2L1 |
| resveratrol | CFLAR | sucrose | PGR | trans-resveratrol | HSPA4 |
| resveratrol | CASP2 | sucrose | GANC | trans-resveratrol | CBR4 |
| resveratrol | KLK3 | sucrose | TSPO | trans-resveratrol | FOS |
| resveratrol | CDC42 | sucrose | SULT4A1 | trans-resveratrol | IL8 |
| resveratrol | CD209 | sucrose | UGP2 | trans-resveratrol | CYCS |
| resveratrol | NQO1 | sucrose | BRIX1 | trans-resveratrol | GLUL |
| resveratrol | ADIPOQ | sucrose | CAV1 | trans-resveratrol | SULT1B1 |
| resveratrol | SLC2A4 | sucrose | RAD1 | trans-resveratrol | OLR1 |
| resveratrol | GSK3B | sucrose | AMY2B | trans-resveratrol | LPL |
| resveratrol | NOS2 | sucrose | CAV3 | trans-resveratrol | CASP3 |
| resveratrol | HBG1 | sucrose | RNASE1 | trans-resveratrol | UCP2 |
| resveratrol | SIRT7 | sucrose | TAS1R3 | trans-resveratrol | CFLAR |
| resveratrol | BCL2 | sucrose | S100A4 | trans-resveratrol | CASP2 |
| resveratrol | CASP9 | sucrose | SREBF1 | trans-resveratrol | KLK3 |
| resveratrol | CD86 | sucrose | DNAH8 | trans-resveratrol | CDC42 |
| resveratrol | BSG | sucrose | CES1 | trans-resveratrol | CD209 |
| resveratrol | ABCC5 | sucrose | PTGS2 | trans-resveratrol | NQO1 |
| resveratrol | F3 | sucrose | CNR1 | trans-resveratrol | ADIPOQ |
| resveratrol | NR1I2 | sucrose | AMY1A | trans-resveratrol | SLC2A4 |
| resveratrol | SOD2 | sucrose | AR | trans-resveratrol | GSK3B |
| resveratrol | NQO2 | sucrose | TP53INP2 | trans-resveratrol | NOS2 |
| resveratrol | CYP3A4 | sucrose | TAS1R2 | trans-resveratrol | HBG1 |
| resveratrol | HIF1A | sucrose | CASZ1 | trans-resveratrol | SIRT7 |
| resveratrol | HBG2 | sucrose | SLC2A5 | trans-resveratrol | BCL2 |
| resveratrol | IGF2 | sucrose | TNFRSF25 | trans-resveratrol | CASP9 |
| resveratrol | CAV1 | sucrose | EDN1 | trans-resveratrol | CD86 |
| resveratrol | FOXO3 | sucrose | PIGA | trans-resveratrol | BSG |
| resveratrol | PTK2 | sucrose | GPR109A | trans-resveratrol | ABCC5 |
| resveratrol | CYP1A2 | sucrose | G6PD | trans-resveratrol | F3 |
| resveratrol | PTGES | sucrose | AMY2A | trans-resveratrol | NR1I2 |
| resveratrol | ADCY2 | sucrose | CXCR4 | trans-resveratrol | SOD2 |
| resveratrol | ESR2 | sucrose | FLOT1 | trans-resveratrol | NQO2 |
| resveratrol | ATF3 | sucrose | TNF | trans-resveratrol | CYP3A4 |
| resveratrol | PRKAA1 | sucrose | MYH14 | trans-resveratrol | HIF1A |
| resveratrol | PDE5A | sucrose | BDNF | trans-resveratrol | HBG2 |
| resveratrol | C5AR1 | taxifolin | APOB | trans-resveratrol | IGF2 |
| resveratrol | SREBF1 | taxifolin | SPAG9 | trans-resveratrol | CAV1 |
| resveratrol | SRC | taxifolin | ICAM1 | trans-resveratrol | FOXO3 |
| resveratrol | PIK3CG | taxifolin | MTTP | trans-resveratrol | PTK2 |
| resveratrol | RUNX2 | taxifolin | NQO1 | trans-resveratrol | CYP1A2 |
| resveratrol | MAPK8 | taxifolin | ABCC1 | trans-resveratrol | PTGES |
| resveratrol | MTOR | trans-resveratrol | TNS1 | trans-resveratrol | ADCY2 |
| resveratrol | GLI2 | trans-resveratrol | PPP1R13B | trans-resveratrol | ESR2 |
| resveratrol | PTGS1 | trans-resveratrol | ESR1 | trans-resveratrol | ATF3 |
| resveratrol | CDKN2A | trans-resveratrol | SIRT1 | trans-resveratrol | PRKAA1 |
| resveratrol | RAGE | trans-resveratrol | MAPK1 | trans-resveratrol | PDE5A |
| resveratrol | PARP1 | trans-resveratrol | XBP1 | trans-resveratrol | C5AR1 |
| resveratrol | PTGS2 | trans-resveratrol | HMOX1 | trans-resveratrol | SREBF1 |
| resveratrol | FASLG | trans-resveratrol | APEX1 | trans-resveratrol | SRC |
| resveratrol | BGLAP | trans-resveratrol | NFKBIA | trans-resveratrol | PIK3CG |
| resveratrol | GNAS | trans-resveratrol | CSNK2A1 | trans-resveratrol | RUNX2 |
| resveratrol | JUN | trans-resveratrol | FAM48A | trans-resveratrol | MAPK8 |
| resveratrol | PRKAA2 | trans-resveratrol | MMP2 | trans-resveratrol | MTOR |
| resveratrol | PTEN | trans-resveratrol | GSR | trans-resveratrol | GLI2 |
| resveratrol | VEGFA | trans-resveratrol | CYP4F2 | trans-resveratrol | PTGS1 |
| resveratrol | CEL | trans-resveratrol | TGFB1 | trans-resveratrol | CDKN2A |
| resveratrol | MMP9 | trans-resveratrol | GSK3A | trans-resveratrol | RAGE |
| resveratrol | NOX1 | trans-resveratrol | PON1 | trans-resveratrol | PARP1 |
| resveratrol | RPS6KA1 | trans-resveratrol | RPS6KB1 | trans-resveratrol | PTGS2 |
| resveratrol | BAK1 | trans-resveratrol | SULT1E1 | trans-resveratrol | FASLG |
| resveratrol | KLF4 | trans-resveratrol | NFKB1 | trans-resveratrol | BGLAP |
| resveratrol | AR | trans-resveratrol | CCND1 | trans-resveratrol | GNAS |
| resveratrol | NPPB | trans-resveratrol | LTA4H | trans-resveratrol | JUN |
| resveratrol | NPPA | trans-resveratrol | IFNG | trans-resveratrol | PRKAA2 |
| resveratrol | HLA-A | trans-resveratrol | MAPK14 | trans-resveratrol | PTEN |
| resveratrol | ABCC4 | trans-resveratrol | BCL6 | trans-resveratrol | VEGFA |
| resveratrol | MYC | trans-resveratrol | APOB | trans-resveratrol | CEL |
| resveratrol | EDN1 | trans-resveratrol | PRDM2 | trans-resveratrol | MMP9 |
| resveratrol | FOXO1 | trans-resveratrol | CTSD | trans-resveratrol | NOX1 |
| resveratrol | CYP1A1 | trans-resveratrol | TTR | trans-resveratrol | RPS6KA1 |
| resveratrol | DIO2 | trans-resveratrol | TNFSF11 | trans-resveratrol | BAK1 |
| resveratrol | ABCC8 | trans-resveratrol | EGR1 | trans-resveratrol | KLF4 |
| resveratrol | ABCC1 | trans-resveratrol | CAT | trans-resveratrol | AR |
| resveratrol | RELA | trans-resveratrol | TNFSF10 | trans-resveratrol | NPPB |
| resveratrol | TNF | trans-resveratrol | AHR | trans-resveratrol | NPPA |
| resveratrol | WEE1 | trans-resveratrol | NPY | trans-resveratrol | HLA-A |
| resveratrol | GPX1 | trans-resveratrol | CD97 | trans-resveratrol | ABCC4 |
| resveratrol | TOP2A | trans-resveratrol | VASP | trans-resveratrol | MYC |
| rutin | GSR | trans-resveratrol | KLF2 | trans-resveratrol | EDN1 |
| rutin | ABCG2 | trans-resveratrol | SIRT2 | trans-resveratrol | FOXO1 |
| rutin | CYP1B1 | trans-resveratrol | ALOX12 | trans-resveratrol | CYP1A1 |
| rutin | ADRA2A | trans-resveratrol | GDF15 | trans-resveratrol | DIO2 |
| rutin | CHRM5 | trans-resveratrol | CCNA1 | trans-resveratrol | ABCC8 |
| rutin | MYLIP | trans-resveratrol | EIF2S1 | trans-resveratrol | ABCC1 |
| rutin | VEGFA | trans-resveratrol | IL6 | trans-resveratrol | RELA |
| rutin | GBA2 | trans-resveratrol | TLR2 | trans-resveratrol | TNF |
| rutin | AKR1C3 | trans-resveratrol | MMP13 | trans-resveratrol | WEE1 |
| rutin | ADRA2C | trans-resveratrol | CYP19A1 | trans-resveratrol | GPX1 |
| uridine | PYGM | trans-resveratrol | CYP1B1 | trans-resveratrol | TOP2A |
| uridine | PYGL | uridine | UCK2 | tryptophan | DAO |
| uridine | PYGB | uridine | DKC1 | tryptophan | IFNG |
| uridine | DHODH | uridine | SLC29A1 | tryptophan | WARS2 |
| uridine | RNASEH2A | uridine | GBGT1 | tryptophan | UROD |
| uridine | GAR1 | uridine | UCK1 | tryptophan | TPH1 |
| uridine | APOBEC1 | uridine | ADA | tryptophan | IDO1 |
| uridine | AICDA | uridine | SLC29A3 | tryptophan | KYNU |
| uridine | UMPS | uridine | UPRT | tryptophan | HPX |
| uridine | EIF2AK2 | uridine | P2RY4 | tryptophan | SLC16A2 |
| uridine | APOB | uridine | CDA | tryptophan | CRH |
| uridine | NT5C1A | uridine | SLC28A3 | tryptophan | TDO2 |
| uridine | NT5C3 | uridine | PUS1 | tryptophan | ALB |
| uridine | NT5C | uridine | NT5M | tryptophan | PRL |
| uridine | SLC35A2 | uridine | RPL13A | tryptophan | SLC6A19 |
| uridine | TYMP | uridine | ELAVL1 | tryptophan | CHRM1 |
| uridine | NT5E | uridine | UPP2 | tryptophan | SLC7A8 |
| uridine | IFNA1 | uridine | TNF | tryptophan | F2R |
| uridine | ADK | uridine | LSM2 | tryptophan | AFMID |
| uridine | DCK | uridine | TYMS | tryptophan | TPH2 |
| uridine | SLC28A1 | uridine | UGT2B17 | tryptophan | SYNM |
| uridine | LSM6 | uridine | UPP1 | tryptophan | IL4I1 |
| uridine | SLC45A2 | uridine | NOP10 | tryptophan | WARS |
| uridine | TRUB1 | uridine | UGP2 | tryptophan | DDC |
| uridine | UGT2B7 | uridine | NT5C2 | tryptophan | KMO |
| uridine | P2RY6 | uridine | UGT2B15 | tryptophan | SLC16A10 |
| uridine | P2RY2 | uridine | RNASE1 | tryptophan | NGF |
| uridine | SLC28A2 | uridine | UCKL1 | tryptophan | SLC3A2 |
| uridine | PNP | uridine | SLC29A2 | tryptophan | HTR2A |
| tryptophan | CHRM4 | uridine | NT5C1B-RDH14 | tryptophan | LYST |

**Supplement Table S7** GO enrichment analysis result of Y-Y-T formula.

| **GO Term** | **GO Levels** | **Nr Count** | **%**  **Associated Genes** | **Term P-Value** | **Benjamini** |
| --- | --- | --- | --- | --- | --- |
| heparin binding | 5 | 39 | 0.2 | 2.40E-14 | 1.20E-11 |
| peptide receptor activity, G-protein coupled | 5 | 38 | 0.2 | 4.90E-12 | 1.20E-09 |
| neuropeptide hormone activity | 5 | 14 | 0.1 | 1.90E-08 | 3.10E-06 |
| DNA N-glycosylase activity | 5 | 9 | 0.1 | 7.40E-07 | 9.10E-05 |
| serine-type endopeptidase activity | 5 | 34 | 0.2 | 4.40E-06 | 4.40E-04 |
| neuropeptide receptor activity | 5 | 15 | 0.1 | 8.30E-06 | 6.80E-04 |
| aldehyde dehydrogenase (NAD) activity | 5 | 7 | 0 | 1.60E-05 | 1.10E-03 |
| serine-type peptidase activity | 5 | 35 | 0.2 | 4.20E-05 | 2.60E-03 |
| phospholipase activity | 5 | 20 | 0.1 | 1.90E-04 | 1.00E-02 |
| nucleoside transmembrane transporter activity | 5 | 6 | 0 | 3.10E-04 | 1.50E-02 |
| magnesium ion binding | 5 | 65 | 0.4 | 4.70E-04 | 2.10E-02 |
| phosphoric diester hydrolase activity | 5 | 19 | 0.1 | 6.40E-04 | 2.60E-02 |
| FAD binding | 5 | 17 | 0.1 | 8.40E-04 | 3.10E-02 |
| aldehyde dehydrogenase [NAD(P)+] activity | 5 | 5 | 0 | 9.80E-04 | 3.40E-02 |
| symporter activity | 5 | 25 | 0.2 | 1.90E-03 | 6.10E-02 |
| nucleobase transmembrane transporter activity | 5 | 5 | 0 | 2.10E-03 | 6.40E-02 |
| peptide hormone receptor binding | 5 | 5 | 0 | 2.10E-03 | 6.40E-02 |
| endopeptidase activity | 5 | 53 | 0.3 | 2.50E-03 | 6.90E-02 |
| choline transmembrane transporter activity | 5 | 4 | 0 | 3.10E-03 | 8.10E-02 |
| S-adenosylmethionine-dependent methyltransferase activity | 5 | 17 | 0.1 | 4.50E-03 | 1.10E-01 |
| steroid hormone receptor activity | 5 | 12 | 0.1 | 4.80E-03 | 1.10E-01 |
| aromatase activity | 5 | 8 | 0 | 6.60E-03 | 1.40E-01 |
| amine oxidase activity | 5 | 4 | 0 | 7.10E-03 | 1.50E-01 |
| muscarinic acetylcholine receptor activity | 5 | 4 | 0 | 7.10E-03 | 1.50E-01 |
| phosphorylase activity | 5 | 4 | 0 | 7.10E-03 | 1.50E-01 |
| vasopressin receptor activity | 5 | 4 | 0 | 7.10E-03 | 1.50E-01 |
| 1-alkyl-2-acetylglycerophosphocholine esterase activity | 5 | 4 | 0 | 7.10E-03 | 1.50E-01 |
| thrombin receptor activity | 5 | 4 | 0 | 7.10E-03 | 1.50E-01 |
| G-protein coupled acetylcholine receptor activity | 5 | 4 | 0 | 7.10E-03 | 1.50E-01 |
| aldo-keto reductase activity | 5 | 6 | 0 | 9.70E-03 | 1.90E-01 |
| lipoxygenase activity | 5 | 4 | 0 | 1.30E-02 | 2.40E-01 |
| promoter binding | 5 | 12 | 0.1 | 1.50E-02 | 2.60E-01 |
| nucleotide receptor activity | 5 | 9 | 0.1 | 1.60E-02 | 2.70E-01 |
| double-stranded DNA binding | 5 | 17 | 0.1 | 1.80E-02 | 2.80E-01 |
| protein kinase binding | 5 | 23 | 0.1 | 1.80E-02 | 2.80E-01 |
| heparan sulfate sulfotransferase activity | 5 | 5 | 0 | 2.10E-02 | 3.00E-01 |
| cholesterol transporter activity | 5 | 5 | 0 | 2.10E-02 | 3.00E-01 |
| serine-type endopeptidase inhibitor activity | 5 | 16 | 0.1 | 2.30E-02 | 3.20E-01 |
| nitric-oxide synthase activity | 5 | 3 | 0 | 2.50E-02 | 3.30E-01 |
| bombesin receptor binding | 5 | 3 | 0 | 2.50E-02 | 3.30E-01 |
| bradykinin receptor activity | 5 | 3 | 0 | 2.50E-02 | 3.30E-01 |
| DNA ligase (ATP) activity | 5 | 3 | 0 | 2.50E-02 | 3.30E-01 |
| tachykinin receptor activity | 5 | 3 | 0 | 2.50E-02 | 3.30E-01 |
| vasopressin receptor binding | 5 | 3 | 0 | 2.50E-02 | 3.30E-01 |
| amylase activity | 5 | 3 | 0 | 2.50E-02 | 3.30E-01 |
| cholinesterase activity | 5 | 3 | 0 | 2.50E-02 | 3.30E-01 |
| pyridoxal phosphate binding | 5 | 11 | 0.1 | 2.70E-02 | 3.40E-01 |
| protein kinase activity | 5 | 72 | 0.4 | 2.70E-02 | 3.40E-01 |
| sugar transmembrane transporter activity | 5 | 7 | 0 | 3.00E-02 | 3.60E-01 |
| CDP-alcohol phosphatidyltransferase activity | 5 | 4 | 0 | 3.20E-02 | 3.70E-01 |
| adenyl ribonucleotide binding | 5 | 160 | 1 | 4.10E-02 | 4.40E-01 |
| nucleobase, nucleoside, nucleotide kinase activity | 5 | 9 | 0.1 | 4.40E-02 | 4.50E-01 |
| quaternary ammonium group transmembrane transporter activity | 5 | 3 | 0 | 4.60E-02 | 4.60E-01 |
| acetate-CoA ligase activity | 5 | 3 | 0 | 4.60E-02 | 4.60E-01 |
| 1-phosphatidylinositol 4-kinase activity | 5 | 3 | 0 | 4.60E-02 | 4.60E-01 |
| lipid kinase activity | 5 | 7 | 0 | 4.90E-02 | 4.70E-01 |
| glucuronosyltransferase activity | 5 | 6 | 0 | 4.90E-02 | 4.60E-01 |
| triacylglycerol lipase activity | 5 | 5 | 0 | 5.60E-02 | 5.00E-01 |
| phosphoinositide 3-kinase binding | 5 | 4 | 0 | 6.00E-02 | 5.20E-01 |
| NAD binding | 5 | 4 | 0 | 6.00E-02 | 5.20E-01 |
| DNA-methyltransferase activity | 5 | 3 | 0 | 7.20E-02 | 5.80E-01 |
| secondary active monocarboxylate transmembrane transporter activity | 5 | 3 | 0 | 7.20E-02 | 5.80E-01 |
| 3-chloroallyl aldehyde dehydrogenase activity | 5 | 3 | 0 | 7.20E-02 | 5.80E-01 |
| lipopolysaccharide receptor activity | 5 | 3 | 0 | 7.20E-02 | 5.80E-01 |
| low-density lipoprotein receptor binding | 5 | 4 | 0 | 7.60E-02 | 5.90E-01 |
| heparan sulfate proteoglycan binding | 5 | 4 | 0 | 7.60E-02 | 5.90E-01 |
| acetylcholine receptor activity | 5 | 5 | 0 | 8.10E-02 | 6.10E-01 |
| chemokine receptor binding | 5 | 9 | 0.1 | 8.30E-02 | 6.10E-01 |
| deoxyribonuclease activity | 5 | 7 | 0 | 9.30E-02 | 6.40E-01 |
| FMN binding | 5 | 4 | 0 | 9.50E-02 | 6.40E-01 |

**Supplement Table S8** KEGG pathway enrichment analysis result of Y-Y-T targets.

| **Pathway** | **Gens number** | **P-Value** | **Benjamini** | **Class** |
| --- | --- | --- | --- | --- |
| Metabolic pathways | 252 | 3.50E-21 | 1.00E-18 | Metabolism |
| Pathways in cancer | 107 | 9.60E-17 | 1.60E-14 | Human Diseases |
| Complement and coagulation cascades | 36 | 4.70E-15 | 4.40E-13 | Organismal Systems |
| Proteoglycans in cancer | 62 | 1.30E-12 | 9.50E-11 | Human Diseases |
| Prostate cancer | 37 | 3.10E-12 | 1.70E-10 | Human Diseases |
| Neuroactive ligand-receptor interaction | 76 | 4.80E-12 | 2.30E-10 | Environmental Information Processing |
| TNF signaling pathway | 39 | 1.40E-10 | 5.80E-09 | Environmental Information Processing |
| Choline metabolism in cancer | 38 | 1.70E-10 | 5.90E-09 | Human Diseases |
| Calcium signaling pathway | 53 | 2.80E-10 | 9.00E-09 | Environmental Information Processing |
| Insulin resistance | 39 | 3.70E-10 | 1.10E-08 | Human Diseases |
| Tryptophan metabolism | 22 | 6.60E-10 | 1.70E-08 | Metabolism |
| Hepatitis B | 46 | 7.70E-10 | 1.80E-08 | Human Diseases |
| Pancreatic cancer | 28 | 1.30E-09 | 3.00E-08 | Human Diseases |
| PI3K-Akt signaling pathway | 82 | 1.50E-09 | 3.10E-08 | Environmental Information Processing |
| Tyrosine metabolism | 20 | 2.10E-09 | 4.00E-08 | Metabolism |
| Apoptosis | 27 | 3.20E-09 | 5.70E-08 | Cellular Processes |
| Prolactin signaling pathway | 29 | 4.20E-09 | 7.00E-08 | Organismal Systems |
| Bladder cancer | 21 | 5.20E-09 | 8.30E-08 | Human Diseases |
| Drug metabolism - other enzymes | 17 | 7.20E-09 | 1.10E-07 | Metabolism |
| Malaria | 23 | 1.10E-08 | 1.60E-07 | Human Diseases |
| FoxO signaling pathway | 41 | 1.80E-08 | 2.50E-07 | Environmental Information Processing |
| Base excision repair | 18 | 2.30E-08 | 3.00E-07 | Genetic Information Processing |
| Chagas disease (American trypanosomiasis) | 35 | 2.70E-08 | 3.30E-07 | Human Diseases |
| HIF-1 signaling pathway | 33 | 7.10E-08 | 8.50E-07 | Environmental Information Processing |
| Adipocytokine signaling pathway | 26 | 2.80E-07 | 3.20E-06 | Organismal Systems |
| Colorectal cancer | 24 | 3.80E-07 | 4.20E-06 | Human Diseases |
| Melanoma | 25 | 8.50E-07 | 9.00E-06 | Human Diseases |
| Pyruvate metabolism | 18 | 1.40E-06 | 1.40E-05 | Metabolism |
| Influenza A | 44 | 3.00E-06 | 2.90E-05 | Human Diseases |
| Central carbon metabolism in cancer | 23 | 3.00E-06 | 2.90E-05 | Human Diseases |
| Rap1 signaling pathway | 50 | 3.60E-06 | 3.30E-05 | Environmental Information Processing |
| Histidine metabolism | 13 | 3.70E-06 | 3.30E-05 | Metabolism |
| Phenylalanine metabolism | 11 | 5.90E-06 | 5.10E-05 | Metabolism |
| ErbB signaling pathway | 27 | 7.70E-06 | 6.40E-05 | Environmental Information Processing |
| Measles | 35 | 1.00E-05 | 8.50E-05 | Human Diseases |
| African trypanosomiasis | 15 | 1.20E-05 | 9.80E-05 | Human Diseases |
| beta-Alanine metabolism | 14 | 3.00E-05 | 2.30E-04 | Metabolism |
| Non-alcoholic fatty liver disease (NAFLD) | 37 | 3.20E-05 | 2.40E-04 | Human Diseases |
| Estrogen signaling pathway | 28 | 3.30E-05 | 2.40E-04 | Organismal Systems |
| Toxoplasmosis | 31 | 5.20E-05 | 3.70E-04 | Human Diseases |
| Inflammatory mediator regulation of TRP channels | 27 | 6.30E-05 | 4.40E-04 | Organismal Systems |
| AMPK signaling pathway | 31 | 7.30E-05 | 5.00E-04 | Environmental Information Processing |
| Endometrial cancer | 18 | 8.30E-05 | 5.50E-04 | Human Diseases |
| Chronic myeloid leukemia | 22 | 8.50E-05 | 5.50E-04 | Human Diseases |
| Galactose metabolism | 13 | 1.00E-04 | 6.60E-04 | Metabolism |
| Toll-like receptor signaling pathway | 28 | 1.20E-04 | 7.30E-04 | Organismal Systems |
| Ras signaling pathway | 48 | 1.30E-04 | 7.80E-04 | Environmental Information Processing |
| Small cell lung cancer | 24 | 1.40E-04 | 8.40E-04 | Human Diseases |
| T cell receptor signaling pathway | 27 | 1.60E-04 | 9.10E-04 | Organismal Systems |
| GnRH signaling pathway | 25 | 1.60E-04 | 9.00E-04 | Organismal Systems |
| NOD-like receptor signaling pathway | 18 | 1.80E-04 | 1.00E-03 | Organismal Systems |
| Neurotrophin signaling pathway | 30 | 1.80E-04 | 1.00E-03 | Organismal Systems |
| VEGF signaling pathway | 19 | 2.30E-04 | 1.20E-03 | Environmental Information Processing |
| Non-small cell lung cancer | 18 | 2.30E-04 | 1.20E-03 | Human Diseases |
| Glucagon signaling pathway | 26 | 2.40E-04 | 1.30E-03 | Organismal Systems |
| Biosynthesis of antibiotics | 45 | 2.40E-04 | 1.20E-03 | Metabolism |
| Cysteine and methionine metabolism | 14 | 2.50E-04 | 1.30E-03 | Metabolism |
| Glycolysis / Gluconeogenesis | 20 | 2.70E-04 | 1.30E-03 | Metabolism |
| Glioma | 19 | 3.60E-04 | 1.70E-03 | Human Diseases |
| mTOR signaling pathway | 18 | 3.70E-04 | 1.80E-03 | Environmental Information Processing |
| Alanine, aspartate and glutamate metabolism | 13 | 4.20E-04 | 1.90E-03 | Metabolism |
| Insulin signaling pathway | 32 | 4.50E-04 | 2.10E-03 | Organismal Systems |
| Pertussis | 21 | 4.70E-04 | 2.10E-03 | Human Diseases |
| Leishmaniasis | 20 | 6.10E-04 | 2.70E-03 | Human Diseases |
| Arginine and proline metabolism | 16 | 6.20E-04 | 2.70E-03 | Metabolism |
| Amoebiasis | 26 | 7.40E-04 | 3.20E-03 | Human Diseases |
| Epithelial cell signaling in Helicobacter pylori infection | 19 | 8.00E-04 | 3.40E-03 | Human Diseases |
| Arachidonic acid metabolism | 18 | 8.60E-04 | 3.60E-03 | Metabolism |
| MAPK signaling pathway | 49 | 1.10E-03 | 4.50E-03 | Environmental Information Processing |
| Prion diseases | 12 | 1.20E-03 | 5.10E-03 | Human Diseases |
| Inflammatory bowel disease (IBD) | 18 | 1.30E-03 | 5.10E-03 | Human Diseases |
| Chemokine signaling pathway | 38 | 1.40E-03 | 5.50E-03 | Organismal Systems |
| Renal cell carcinoma | 18 | 1.50E-03 | 6.00E-03 | Human Diseases |
| Amyotrophic lateral sclerosis (ALS) | 15 | 1.90E-03 | 7.50E-03 | Human Diseases |
| Ether lipid metabolism | 14 | 2.10E-03 | 7.80E-03 | Metabolism |
| Sphingolipid signaling pathway | 27 | 2.20E-03 | 8.20E-03 | Environmental Information Processing |
| Starch and sucrose metabolism | 16 | 2.20E-03 | 8.10E-03 | Metabolism |
| Acute myeloid leukemia | 16 | 2.20E-03 | 8.10E-03 | Human Diseases |
| Serotonergic synapse | 25 | 2.20E-03 | 8.10E-03 | Organismal Systems |
| Rheumatoid arthritis | 21 | 2.90E-03 | 1.00E-02 | Human Diseases |

**Supplement Table S9** Differentially expressed genes of AS.

| **Gene name** | **P-value** | **Attribute** | **Gene name** | **P-value** | **Attribute** |
| --- | --- | --- | --- | --- | --- |
| SNIP1 | 0.00542807 | Down-regulated | C7ORF11 | 0.010703826 | Down-regulated |
| BCL6B | 0.006111887 | Down-regulated | ILF2 | 0.039219802 | Down-regulated |
| SERTAD2 | 0.001690608 | Down-regulated | ZMAT5 | 0.002989585 | Down-regulated |
| BRD9 | 0.007611852 | Down-regulated | POLR2F | 0.003065413 | Down-regulated |
| DCK | 0.006350987 | Down-regulated | FBXO21 | 0.048779687 | Down-regulated |
| NCOA6IP | 0.007595217 | Down-regulated | LOC641849 | 0.029779202 | Down-regulated |
| LOC649150 | 0.003290824 | Down-regulated | IRAK2 | 0.001412984 | Down-regulated |
| TGOLN2 | 0.008499044 | Down-regulated | SDHC | 0.044166649 | Down-regulated |
| UBIAD1 | 0.028685124 | Down-regulated | RPS25 | 0.000340487 | Down-regulated |
| HMGN1 | 0.005886797 | Down-regulated | POLR1C | 0.01106732 | Down-regulated |
| COX4I1 | 0.008212686 | Down-regulated | TRAPPC2L | 0.007741443 | Down-regulated |
| LOC647361 | 0.003310768 | Down-regulated | DDX47 | 0.009983064 | Down-regulated |
| SUCLG2 | 0.043146662 | Down-regulated | CCL3L1 | 0.001903374 | Down-regulated |
| HIC2 | 0.022168867 | Down-regulated | POLR2G | 0.001856676 | Down-regulated |
| LOC440589 | 0.04927015 | Down-regulated | GIMAP4 | 0.037628335 | Down-regulated |
| ATP5J | 0.00251219 | Down-regulated | MAPK13 | 0.021135797 | Down-regulated |
| PLAC8 | 0.00925529 | Down-regulated | STX5 | 0.001853427 | Down-regulated |
| C8ORF59 | 0.025687297 | Down-regulated | MRPL32 | 0.007614653 | Down-regulated |
| RPL12 | 0.00100656 | Down-regulated | NDUFB11 | 0.022302734 | Down-regulated |
| C14ORF80 | 0.004926814 | Down-regulated | RAB9A | 0.013592296 | Down-regulated |
| SCMH1 | 0.033848837 | Down-regulated | NCR1 | 0.035212575 | Down-regulated |
| MRLC2 | 0.006875753 | Down-regulated | ARHGAP30 | 0.005917823 | Down-regulated |
| PARD6A | 0.025530881 | Down-regulated | PACSIN1 | 0.00561871 | Down-regulated |
| COX6C | 0.008644456 | Down-regulated | SKAP1 | 0.009460904 | Down-regulated |
| TXN2 | 0.049851486 | Down-regulated | NUDCD2 | 0.046327582 | Down-regulated |
| LOC649169 | 0.008236478 | Down-regulated | LOC649049 | 0.034442463 | Down-regulated |
| CECR5 | 0.004394546 | Down-regulated | MGST3 | 0.021343883 | Down-regulated |
| SERF1B | 0.031696387 | Down-regulated | TMEM60 | 0.002980944 | Down-regulated |
| PSMC6 | 0.004442164 | Down-regulated | P2RY11 | 0.003069703 | Down-regulated |
| RIOK2 | 0.003482432 | Down-regulated | KIAA1143 | 0.030897683 | Down-regulated |
| MRPS22 | 0.013018361 | Down-regulated | KLRC1 | 0.009396522 | Down-regulated |
| RPL17 | 0.001138521 | Down-regulated | STX8 | 0.010157217 | Down-regulated |
| TSPAN3 | 0.014777441 | Down-regulated | HNRNPA0 | 0.001552335 | Down-regulated |
| ARPC3 | 0.017059233 | Down-regulated | OSGEP | 0.020080349 | Down-regulated |
| MRPS21 | 0.004743919 | Down-regulated | EIF2B1 | 0.023815878 | Down-regulated |
| PES1 | 0.031606027 | Down-regulated | GTF2A2 | 0.020419435 | Down-regulated |
| NMT2 | 0.031897005 | Down-regulated | HDDC2 | 0.003098317 | Down-regulated |
| SNRPN | 0.047948824 | Down-regulated | LOC654121 | 0.023672518 | Down-regulated |
| RPS6 | 0.018158697 | Down-regulated | RPL31 | 0.009832298 | Down-regulated |
| RPL27 | 0.002897091 | Down-regulated | RPL30 | 0.00048667 | Down-regulated |
| MDH1 | 0.021579553 | Down-regulated | AYP1P1 | 0.013669112 | Down-regulated |
| ALKBH5 | 0.003442667 | Down-regulated | MED19 | 0.019722669 | Down-regulated |
| GALT | 0.000653583 | Down-regulated | AP1M1 | 0.031302682 | Down-regulated |
| LOC285900 | 0.014413033 | Down-regulated | C1ORF162 | 0.000656532 | Down-regulated |
| ZMYM6 | 0.000262473 | Down-regulated | ARL2BP | 0.044554751 | Down-regulated |
| ZNF644 | 0.024097627 | Down-regulated | RPLP2 | 0.038456788 | Down-regulated |
| FIBP | 0.004403758 | Down-regulated | C13ORF7 | 0.048669937 | Down-regulated |
| TMEM14C | 0.018603481 | Down-regulated | DBI | 0.000107115 | Down-regulated |
| RHEB | 0.000981544 | Down-regulated | ALDOC | 0.003661153 | Down-regulated |
| KLRG1 | 0.041859021 | Down-regulated | UFSP2 | 0.025852485 | Down-regulated |
| MRPS18C | 0.000253811 | Down-regulated | NDUFS3 | 0.022232956 | Down-regulated |
| SFXN4 | 0.016895634 | Down-regulated | RPL35A | 0.004524212 | Down-regulated |
| C5ORF44 | 0.023332126 | Down-regulated | SCRN1 | 0.022707608 | Down-regulated |
| LOC653884 | 0.003372768 | Down-regulated | MXD4 | 0.006029874 | Down-regulated |
| KRTCAP2 | 0.003456204 | Down-regulated | GIMAP1 | 0.004580726 | Down-regulated |
| ARAF | 0.000431198 | Down-regulated | RAN | 0.001770925 | Down-regulated |
| LOC647349 | 0.000100028 | Down-regulated | LOC647673 | 0.036690892 | Down-regulated |
| DPM1 | 0.004568221 | Down-regulated | ALKBH1 | 0.016943273 | Down-regulated |
| ACOT9 | 0.00963745 | Down-regulated | PRPS2 | 0.029424487 | Down-regulated |
| MTMR14 | 0.014333184 | Down-regulated | COPS7A | 0.00907796 | Down-regulated |
| PRPF6 | 0.024293482 | Down-regulated | PRKRA | 0.030318637 | Down-regulated |
| C6ORF66 | 0.027445852 | Down-regulated | FADD | 0.028685124 | Down-regulated |
| LOC388654 | 0.003481374 | Down-regulated | TMEM93 | 0.000770089 | Down-regulated |
| SEC61A1 | 0.043416646 | Down-regulated | BRD2 | 0.000810898 | Down-regulated |
| GALK2 | 0.04921758 | Down-regulated | ZNF689 | 0.001433758 | Down-regulated |
| WWP2 | 0.025530881 | Down-regulated | UQCRB | 0.001174345 | Down-regulated |
| LOC645683 | 0.011121464 | Down-regulated | NDUFA8 | 0.015291832 | Down-regulated |
| C7ORF68 | 0.038806185 | Down-regulated | MGC3731 | 0.027778913 | Down-regulated |
| ACAA2 | 0.040574235 | Down-regulated | DNMT1 | 0.026418356 | Down-regulated |
| PEX5 | 0.03718362 | Down-regulated | NXT1 | 0.013440872 | Down-regulated |
| CPSF4 | 0.013582191 | Down-regulated | LOC643357 | 2.22E-05 | Down-regulated |
| LOC651149 | 0.001186965 | Down-regulated | EOMES | 0.006626928 | Down-regulated |
| ATP5G2 | 0.010037311 | Down-regulated | POLR3GL | 0.0083672 | Down-regulated |
| CRLF3 | 0.031876469 | Down-regulated | RPS3 | 0.00679491 | Down-regulated |
| TCP1 | 0.000490236 | Down-regulated | NDUFA9 | 0.030021182 | Down-regulated |
| DYRK1A | 0.00306567 | Down-regulated | GNL2 | 0.009088376 | Down-regulated |
| PPP2R2B | 0.007683134 | Down-regulated | ALG13 | 0.003365652 | Down-regulated |
| SUCLA2 | 0.000955878 | Down-regulated | ZC3HC1 | 0.003199394 | Down-regulated |
| PAQR4 | 0.01589154 | Down-regulated | HLA-DMB | 0.036859353 | Down-regulated |
| LBH | 0.013793319 | Down-regulated | ALDH9A1 | 0.001095363 | Down-regulated |
| NDUFB8 | 0.043635492 | Down-regulated | LOC647037 | 0.014777441 | Down-regulated |
| PGAM1 | 0.022222815 | Down-regulated | FAM96A | 0.015069049 | Down-regulated |
| C14ORF156 | 0.000805938 | Down-regulated | ORAOV1 | 0.022773664 | Down-regulated |
| ISY1 | 0.011247047 | Down-regulated | QARS | 0.008828454 | Down-regulated |
| RCN2 | 0.001858383 | Down-regulated | PLSCR2 | 0.042570934 | Down-regulated |
| LOC730316 | 0.00181839 | Down-regulated | IER3IP1 | 0.007442031 | Down-regulated |
| RANGAP1 | 0.003127925 | Down-regulated | TM2D1 | 7.36E-05 | Down-regulated |
| DCTN2 | 0.003152173 | Down-regulated | PTRH1 | 0.030797441 | Down-regulated |
| RABL3 | 0.014129267 | Down-regulated | ABCF1 | 0.001403632 | Down-regulated |
| SQSTM1 | 3.46E-05 | Down-regulated | GNPTG | 0.00549533 | Down-regulated |
| PUS1 | 0.04082021 | Down-regulated | LOC124512 | 0.032476481 | Down-regulated |
| CCT7 | 0.004906475 | Down-regulated | GLTP | 0.002566503 | Down-regulated |
| SRP54 | 0.045771554 | Down-regulated | COPS6 | 0.002731081 | Down-regulated |
| MRPL22 | 0.001242633 | Down-regulated | ATP5A1 | 0.035497986 | Down-regulated |
| AMY1C | 0.021033266 | Down-regulated | C3ORF10 | 0.018158697 | Down-regulated |
| LOC642250 | 0.001050528 | Down-regulated | MFNG | 0.036039128 | Down-regulated |
| COX5B | 0.001356684 | Down-regulated | PSMB2 | 0.030239953 | Down-regulated |
| B4GALT7 | 0.000443051 | Down-regulated | EMP3 | 0.012615567 | Down-regulated |
| DGCR6 | 0.000475415 | Down-regulated | COMMD8 | 0.029509226 | Down-regulated |
| CYCSL1 | 0.000442312 | Down-regulated | C19ORF70 | 0.007611852 | Down-regulated |
| CENPB | 0.000618103 | Down-regulated | LOC399942 | 0.001022046 | Down-regulated |
| SEC22C | 0.001076286 | Down-regulated | SBDSP | 0.001487454 | Down-regulated |
| RPF2 | 0.029509226 | Down-regulated | C6ORF48 | 0.004340432 | Down-regulated |
| FAU | 0.006050181 | Down-regulated | COMMD10 | 0.043042409 | Down-regulated |
| TMBIM4 | 0.005733314 | Down-regulated | TMEM126B | 0.000836397 | Down-regulated |
| TMEM138 | 0.015963509 | Down-regulated | UNC50 | 0.000193713 | Down-regulated |
| C19ORF12 | 0.045405208 | Down-regulated | VPS25 | 0.03417992 | Down-regulated |
| GTF2E2 | 0.001331108 | Down-regulated | MAN2B2 | 0.008089097 | Down-regulated |
| SSU72 | 0.025464172 | Down-regulated | LAIR1 | 0.002697899 | Down-regulated |
| LOC648249 | 0.034774233 | Down-regulated | PSMC2 | 0.049352253 | Down-regulated |
| CRTAP | 6.04E-05 | Down-regulated | Septin 9 | 0.025852485 | Down-regulated |
| DIABLO | 0.000270066 | Down-regulated | ARFGAP2 | 0.039949964 | Down-regulated |
| TUBB | 0.049078785 | Down-regulated | LOC651436 | 0.000356297 | Down-regulated |
| LOC648024 | 0.00029195 | Down-regulated | CNBP | 0.004870519 | Down-regulated |
| LOC390466 | 0.000139207 | Down-regulated | GEMIN4 | 0.006334822 | Down-regulated |
| WDR61 | 0.002841599 | Down-regulated | RPAIN | 0.002841599 | Down-regulated |
| CALM3 | 0.001134578 | Down-regulated | ANAPC10 | 0.0458385 | Down-regulated |
| PSMB6 | 0.005635097 | Down-regulated | UBE2V2 | 0.022425124 | Down-regulated |
| UQCRQ | 0.030897683 | Down-regulated | FAM58A | 0.004087282 | Down-regulated |
| SLC25A19 | 0.008499044 | Down-regulated | COQ5 | 0.024348213 | Down-regulated |
| LOC652489 | 7.78E-06 | Down-regulated | ACN9 | 0.034110449 | Down-regulated |
| IRF8 | 0.023884043 | Down-regulated | PEA15 | 0.00060729 | Down-regulated |
| LYPLA1 | 0.018892472 | Down-regulated | C14ORF124 | 0.04883477 | Down-regulated |
| IL8 | 0.018158697 | Down-regulated | LOC728564 | 0.035618748 | Down-regulated |
| GPR114 | 0.022261888 | Down-regulated | NUP37 | 0.02372263 | Down-regulated |
| PLD3 | 0.000656532 | Down-regulated | MAN1B1 | 0.000238301 | Down-regulated |
| FBXO7 | 0.031391668 | Down-regulated | ATP5B | 0.002228129 | Down-regulated |
| RRAS2 | 0.031552245 | Down-regulated | GLG1 | 0.00953923 | Down-regulated |
| C12ORF52 | 0.039164588 | Down-regulated | PDHA1 | 0.023570254 | Down-regulated |
| BRDT | 0.017011107 | Down-regulated | CNOT2 | 0.000191925 | Down-regulated |
| TAF1B | 0.026878878 | Down-regulated | C10ORF32 | 0.025687297 | Down-regulated |
| STK25 | 0.04665237 | Down-regulated | BMS1 | 0.035447434 | Down-regulated |
| CDC37 | 0.000106918 | Down-regulated | PIGK | 0.014784692 | Down-regulated |
| LRRC47 | 0.020169549 | Down-regulated | DYNLL2 | 0.002980944 | Down-regulated |
| CCND3 | 0.003885843 | Down-regulated | LOC645688 | 0.034707656 | Down-regulated |
| ZNF823 | 0.011096506 | Down-regulated | LOC647436 | 0.003461461 | Down-regulated |
| ZNF672 | 0.00031054 | Down-regulated | CDKN2AIP | 0.032884691 | Down-regulated |
| LOC641788 | 0.003278221 | Down-regulated | HSPA5 | 0.0267602 | Down-regulated |
| RASSF5 | 0.000193713 | Down-regulated | U2AF1L2 | 0.01777632 | Down-regulated |
| YWHAZ | 0.000809464 | Down-regulated | SLC25A5 | 0.001514103 | Down-regulated |
| MATK | 0.012798077 | Down-regulated | TMEM141 | 0.000446243 | Down-regulated |
| EI24 | 0.017972966 | Down-regulated | RXRB | 0.000431198 | Down-regulated |
| PAF1 | 0.005856828 | Down-regulated | URM1 | 0.007889868 | Down-regulated |
| ACP1 | 0.047249338 | Down-regulated | HIGD2A | 0.034775477 | Down-regulated |
| NDUFS7 | 0.04682981 | Down-regulated | ZHX3 | 0.00353637 | Down-regulated |
| WDR33 | 0.002708311 | Down-regulated | SNRPB | 0.001419018 | Down-regulated |
| EXOSC10 | 0.005907494 | Down-regulated | LAS1L | 0.013987346 | Down-regulated |
| TSR2 | 0.000582117 | Down-regulated | MT1X | 0.047335597 | Down-regulated |
| C3ORF21 | 4.40E-05 | Down-regulated | LOC400652 | 0.024147896 | Down-regulated |
| MRPL13 | 0.021459486 | Down-regulated | TYSND1 | 0.003278221 | Down-regulated |
| LPIN1 | 0.04682981 | Down-regulated | TRAPPC6A | 0.001153133 | Down-regulated |
| MED30 | 0.00691158 | Down-regulated | PHF5A | 0.045828321 | Down-regulated |
| C10ORF78 | 0.000338611 | Down-regulated | OSTC | 0.00013315 | Down-regulated |
| SAMM50 | 0.008614577 | Down-regulated | LOC148915 | 0.018876311 | Down-regulated |
| TOB1 | 0.048342673 | Down-regulated | GTPBP6 | 0.001187217 | Down-regulated |
| DOLPP1 | 0.018055652 | Down-regulated | PSMD10 | 0.001983124 | Down-regulated |
| ATP5F1 | 0.002802053 | Down-regulated | NDUFS5 | 0.032840773 | Down-regulated |
| PIGY | 0.04855771 | Down-regulated | LOC653505 | 0.001345287 | Down-regulated |
| CRYZ | 0.033907539 | Down-regulated | PIN1 | 0.011247047 | Down-regulated |
| RPL11 | 0.023691695 | Down-regulated | RPL26L1 | 0.0458385 | Down-regulated |
| C10ORF57 | 0.009423413 | Down-regulated | COG2 | 0.035497986 | Down-regulated |
| FBXO8 | 0.034391147 | Down-regulated | INTS9 | 0.0013204 | Down-regulated |
| LOC652624 | 0.014651573 | Down-regulated | VCP | 0.042770286 | Down-regulated |
| CHCHD3 | 0.002939532 | Down-regulated | RPS13 | 0.035410134 | Down-regulated |
| AIMP2 | 0.008323013 | Down-regulated | MED20 | 0.008682124 | Down-regulated |
| RHOC | 0.000111914 | Down-regulated | MAPRE1 | 0.011326009 | Down-regulated |
| SEPHS1 | 0.018281788 | Down-regulated | C8ORF55 | 0.002570215 | Down-regulated |
| LOC646483 | 0.004322733 | Down-regulated | FAM133B | 0.008550208 | Down-regulated |
| KHSRP | 0.005117403 | Down-regulated | C12ORF10 | 0.022865455 | Down-regulated |
| MAT2B | 0.00197109 | Down-regulated | RPRC1 | 0.003772315 | Down-regulated |
| TKTL1 | 0.046096995 | Down-regulated | ACADM | 0.040646868 | Down-regulated |
| IKZF5 | 0.020080349 | Down-regulated | SLC2A6 | 0.010052509 | Down-regulated |
| NCKAP1L | 1.84E-05 | Down-regulated | CCDC28B | 0.001138521 | Down-regulated |
| YWHAQ | 0.004059186 | Down-regulated | WDR41 | 0.029176328 | Down-regulated |
| CXCR6 | 0.018876311 | Down-regulated | TAF9 | 0.004036226 | Down-regulated |
| CIRBP | 0.001983124 | Down-regulated | RTN4RL1 | 0.04892147 | Down-regulated |
| LOC650845 | 0.005696074 | Down-regulated | ZFAND6 | 0.006242134 | Down-regulated |
| LOC283412 | 0.031010688 | Down-regulated | LOC644774 | 0.00063211 | Down-regulated |
| LOC650518 | 0.027042051 | Down-regulated | GZMA | 0.002697899 | Down-regulated |
| QRICH1 | 0.011490144 | Down-regulated | PUF60 | 3.39E-05 | Down-regulated |
| MRPS26 | 0.024862927 | Down-regulated | SUCLG1 | 0.044945578 | Down-regulated |
| LOC388275 | 0.001969628 | Down-regulated | PSMD6 | 0.001803737 | Down-regulated |
| ATP5G1 | 0.032794152 | Down-regulated | FAM96B | 0.015578271 | Down-regulated |
| LOC644330 | 0.040066974 | Down-regulated | PSMC5 | 0.015629784 | Down-regulated |
| LOC390354 | 0.004581259 | Down-regulated | PMM1 | 0.044602683 | Down-regulated |
| CD247 | 0.004191042 | Down-regulated | THAP11 | 0.04682981 | Down-regulated |
| AKR7A2 | 0.037923818 | Down-regulated | LOC400027 | 0.038932014 | Down-regulated |
| SFRS13A | 0.024413313 | Down-regulated | LOC653506 | 0.005700028 | Down-regulated |
| CYFIP2 | 0.035124795 | Down-regulated | ERH | 0.010354866 | Down-regulated |
| CHCHD9 | 0.000100028 | Down-regulated | FASLG | 0.006528935 | Down-regulated |
| C8ORF40 | 0.005881643 | Down-regulated | AP1B1 | 0.00316915 | Down-regulated |
| REXO4 | 0.001803737 | Down-regulated | PAIP2 | 0.028872513 | Down-regulated |
| EXOSC1 | 0.000546765 | Down-regulated | COX6A1 | 0.000825054 | Down-regulated |
| LOC643287 | 0.00963745 | Down-regulated | MRPL34 | 0.002814846 | Down-regulated |
| HIRIP3 | 0.000375241 | Down-regulated | EPRS | 0.026072759 | Down-regulated |
| TTC4 | 0.004721189 | Down-regulated | RRAGA | 0.001814179 | Down-regulated |
| LSM7 | 0.022261888 | Down-regulated | NDUFA1 | 1.44E-05 | Down-regulated |
| MSL3L2 | 0.049646714 | Down-regulated | GNPDA1 | 0.001021679 | Down-regulated |
| SLC37A4 | 0.019034231 | Down-regulated | ATP6V0E2 | 0.022707608 | Down-regulated |
| DBR1 | 0.037265172 | Down-regulated | UBE2G2 | 0.018707897 | Down-regulated |
| SIL1 | 0.030459954 | Down-regulated | POLE3 | 0.000502918 | Down-regulated |
| SNRPF | 0.023522919 | Down-regulated | STAT4 | 0.044155622 | Down-regulated |
| SLC3A2 | 0.001468893 | Down-regulated | ZNF8 | 0.020772143 | Down-regulated |
| NSMCE4A | 0.017011107 | Down-regulated | NEDD8 | 0.0306964 | Down-regulated |
| LOC643997 | 0.003127925 | Down-regulated | MKI67IP | 0.042424489 | Down-regulated |
| CCT2 | 0.006166933 | Down-regulated | LOC649821 | 0.038883891 | Down-regulated |
| TRPM4 | 0.00963745 | Down-regulated | RNF113A | 0.027061551 | Down-regulated |
| LOC730746 | 0.032869666 | Down-regulated | C11ORF10 | 0.001858383 | Down-regulated |
| LOC651202 | 0.026273976 | Down-regulated | ICAM2 | 0.020080349 | Down-regulated |
| FAM120B | 0.004533415 | Down-regulated | SEC61G | 0.00335594 | Down-regulated |
| BRIX1 | 0.038324436 | Down-regulated | HEATR1 | 0.016608532 | Down-regulated |
| GSTP1 | 0.00048667 | Down-regulated | ARL2 | 0.035757951 | Down-regulated |
| LOC284821 | 0.001076286 | Down-regulated | RPS15A | 0.000265422 | Down-regulated |
| RPP38 | 0.007578327 | Down-regulated | LSM4 | 0.008068454 | Down-regulated |
| GTF2IRD2B | 0.015069049 | Down-regulated | NCALD | 0.00497338 | Down-regulated |
| MRRF | 0.02372263 | Down-regulated | PSMB1 | 0.013095584 | Down-regulated |
| ZNF419 | 0.040066974 | Down-regulated | OBFC1 | 0.006166933 | Down-regulated |
| LEMD2 | 0.003480832 | Down-regulated | GNL1 | 0.001853593 | Down-regulated |
| SLC9A3R1 | 1.54E-05 | Down-regulated | NUPL2 | 0.002081963 | Down-regulated |
| CIP29 | 0.003656646 | Down-regulated | LOC644511 | 0.002344681 | Down-regulated |
| SPNS1 | 0.010432292 | Down-regulated | C20ORF20 | 0.008894676 | Down-regulated |
| 1-Mar | 0.002359486 | Down-regulated | SLC25A28 | 0.014316019 | Down-regulated |
| C1ORF41 | 0.023109155 | Down-regulated | METTL13 | 0.047980079 | Down-regulated |
| LOC652271 | 0.022853801 | Down-regulated | PDE6D | 0.025713609 | Down-regulated |
| TMEM69 | 0.001478181 | Down-regulated | C9ORF103 | 0.037789639 | Down-regulated |
| C1ORF59 | 0.025149172 | Down-regulated | LOC642904 | 0.023109155 | Down-regulated |
| C21ORF33 | 0.03417992 | Down-regulated | LOC401206 | 0.005113243 | Down-regulated |
| PAFAH2 | 0.02710374 | Down-regulated | CD7 | 0.024797275 | Down-regulated |
| LYPLAL1 | 0.000228365 | Down-regulated | ARIH2 | 0.010623129 | Down-regulated |
| NKG7 | 0.008014079 | Down-regulated | APIP | 0.005849633 | Down-regulated |
| PSMB10 | 0.002339815 | Down-regulated | UBE2N | 0.020467253 | Down-regulated |
| SNAPC5 | 0.011108331 | Down-regulated | SCAMP3 | 0.041866252 | Down-regulated |
| LOC440737 | 0.015316297 | Down-regulated | TMCO1 | 0.026240319 | Down-regulated |
| TOMM40 | 0.002031879 | Down-regulated | ID2 | 3.39E-05 | Down-regulated |
| EIF3H | 0.021190273 | Down-regulated | MTX2 | 0.035212575 | Down-regulated |
| VPS24 | 0.025530881 | Down-regulated | NDUFS8 | 0.032231907 | Down-regulated |
| LOC286016 | 0.010540937 | Down-regulated | SNRPD3 | 0.021941596 | Down-regulated |
| GPKOW | 0.001721956 | Down-regulated | RASAL3 | 0.039651325 | Down-regulated |
| CIB1 | 0.023214369 | Down-regulated | C6ORF47 | 0.00306567 | Down-regulated |
| IL2RB | 0.00103687 | Down-regulated | RPUSD3 | 0.049415915 | Down-regulated |
| ZFP90 | 0.048083779 | Down-regulated | LTA | 0.047477571 | Down-regulated |
| LOC644322 | 0.046103212 | Down-regulated | POLR3C | 0.019594297 | Down-regulated |
| TRAT1 | 0.03199326 | Down-regulated | PLEK | 0.022630211 | Down-regulated |
| CHI3L2 | 0.02372263 | Down-regulated | DIS3L | 0.026284311 | Down-regulated |
| CNO | 0.002725785 | Down-regulated | VIL2 | 0.018181718 | Down-regulated |
| ASCC1 | 0.00306567 | Down-regulated | C22ORF28 | 0.003092394 | Down-regulated |
| TMEM14B | 0.002555415 | Down-regulated | BCDIN3D | 0.020613466 | Down-regulated |
| C16ORF58 | 0.000306978 | Down-regulated | CD2BP2 | 0.002513155 | Down-regulated |
| LOC728973 | 0.000811846 | Down-regulated | SS18L2 | 0.033907539 | Down-regulated |
| KLRF1 | 0.009777412 | Down-regulated | CLIC3 | 0.013904552 | Down-regulated |
| LOC158345 | 0.002397384 | Down-regulated | B4GALNT4 | 0.039952533 | Down-regulated |
| CBLB | 0.048944233 | Down-regulated | CCDC102A | 0.013154367 | Down-regulated |
| TMEM41A | 0.012787402 | Down-regulated | HSPA14 | 0.032783762 | Down-regulated |
| SDCCAG10 | 0.040646868 | Down-regulated | UROS | 0.001591697 | Down-regulated |
| LOC728739 | 0.025020715 | Down-regulated | PLEKHA3 | 0.023109155 | Down-regulated |
| GFM2 | 0.003661153 | Down-regulated | MRPL17 | 0.001251715 | Down-regulated |
| LOC402644 | 0.001282949 | Down-regulated | KLRD1 | 0.002212712 | Down-regulated |
| RPS10 | 0.049340055 | Down-regulated | COX7C | 0.036540647 | Down-regulated |
| MRPL47 | 0.043931341 | Down-regulated | ATP6V1E1 | 0.047091225 | Down-regulated |
| RFWD3 | 0.013631203 | Down-regulated | ZNF22 | 0.00197109 | Down-regulated |
| MRPS23 | 0.048529076 | Down-regulated | TMEM85 | 0.005716098 | Down-regulated |
| SNAP23 | 0.027778913 | Down-regulated | SOCS2 | 0.030240484 | Down-regulated |
| LOC652773 | 0.000773551 | Down-regulated | SNAPIN | 0.00339686 | Down-regulated |
| DNAJC4 | 0.047091225 | Down-regulated | ACAT1 | 0.028872513 | Down-regulated |
| RAD17 | 0.002422734 | Down-regulated | LOC652826 | 2.27E-06 | Down-regulated |
| PPA2 | 0.015664157 | Down-regulated | MRPL36 | 0.000562939 | Down-regulated |
| SEPHS2 | 0.004798989 | Down-regulated | TUBA1B | 0.000378437 | Down-regulated |
| PSMC4 | 8.26E-05 | Down-regulated | ZNF511 | 0.02426631 | Down-regulated |
| JMJD8 | 0.00013315 | Down-regulated | RPS21 | 0.002737207 | Down-regulated |
| RP9 | 0.004169601 | Down-regulated | RFTN1 | 0.030459954 | Down-regulated |
| SLC25A22 | 0.000262473 | Down-regulated | CFDP1 | 0.030897683 | Down-regulated |
| NAT1 | 0.007813751 | Down-regulated | COQ3 | 0.021613705 | Down-regulated |
| LOC341457 | 0.002661563 | Down-regulated | EMD | 0.001028368 | Down-regulated |
| LCMT1 | 0.025993221 | Down-regulated | CXCR4 | 0.022168565 | Down-regulated |
| RNFT1 | 0.002188319 | Down-regulated | LOC347544 | 0.006624654 | Down-regulated |
| LOC652864 | 0.005474371 | Down-regulated | ATF1 | 0.025612807 | Down-regulated |
| NOL11 | 0.040633097 | Down-regulated | APOBEC3G | 0.019289998 | Down-regulated |
| ZNF329 | 0.021210861 | Down-regulated | AGA | 0.009269899 | Down-regulated |
| LOC730534 | 0.016005206 | Down-regulated | TSSC4 | 0.014650995 | Down-regulated |
| NUBP1 | 0.00241476 | Down-regulated | DIMT1L | 0.000263962 | Down-regulated |
| EIF3G | 0.04277573 | Down-regulated | C9ORF114 | 0.045920222 | Down-regulated |
| RNMTL1 | 0.034668453 | Down-regulated | SNAPC3 | 0.010824163 | Down-regulated |
| HLA-DRA | 0.011982571 | Down-regulated | FAM162A | 0.028706919 | Down-regulated |
| NAE1 | 0.049235859 | Down-regulated | ADA | 0.034103643 | Down-regulated |
| TMEM5 | 0.019948925 | Down-regulated | LOC649555 | 0.005635097 | Down-regulated |
| LOC440927 | 0.017617352 | Down-regulated | ASTE1 | 0.000255745 | Down-regulated |
| MRFAP1L1 | 0.001108115 | Down-regulated | NOL7 | 0.004349861 | Down-regulated |
| ASMTL | 3.03E-05 | Down-regulated | HCP5 | 0.005262761 | Down-regulated |
| MRTO4 | 0.008455592 | Down-regulated | MRPL18 | 0.002008394 | Down-regulated |
| CD81 | 2.58E-05 | Down-regulated | C7ORF27 | 7.00E-05 | Down-regulated |
| NGDN | 0.01934361 | Down-regulated | C12ORF62 | 0.00737942 | Down-regulated |
| HNRPUL1 | 0.006166372 | Down-regulated | THOC7 | 0.029509226 | Down-regulated |
| LOC648343 | 0.025182529 | Down-regulated | TAPBPL | 0.019975455 | Down-regulated |
| TPRKB | 0.000407642 | Down-regulated | LSM8 | 0.024627283 | Down-regulated |
| COMMD3 | 0.01394791 | Down-regulated | PGAP3 | 0.004572605 | Down-regulated |
| GZMM | 0.040446851 | Down-regulated | SIN3A | 0.009036805 | Down-regulated |
| RASSF4 | 0.011233105 | Down-regulated | NT5C | 0.005831952 | Down-regulated |
| HNRPA1P4 | 0.004533415 | Down-regulated | NDUFB5 | 0.025916811 | Down-regulated |
| SNRPB2 | 0.007689002 | Down-regulated | BCAS4 | 0.049106939 | Down-regulated |
| C16ORF87 | 0.006913297 | Down-regulated | C11ORF24 | 0.03794687 | Down-regulated |
| LOC91561 | 0.037074576 | Down-regulated | GSTO1 | 0.006078099 | Down-regulated |
| LOC644162 | 0.002676208 | Down-regulated | TOMM5 | 0.003349264 | Down-regulated |
| PRDM4 | 0.012798077 | Down-regulated | PSMC3 | 0.011833404 | Down-regulated |
| LOC646630 | 0.00215594 | Down-regulated | XBP1 | 0.012843704 | Down-regulated |
| GNPDA2 | 0.02372263 | Down-regulated | SPCS2 | 0.023522919 | Down-regulated |
| GTF3C6 | 0.005708068 | Down-regulated | COX8A | 0.000623518 | Down-regulated |
| LOC645436 | 0.004354532 | Down-regulated | COMMD7 | 0.000190715 | Down-regulated |
| PSMA1 | 0.000412898 | Down-regulated | DNAJC27 | 0.043635492 | Down-regulated |
| BTBD1 | 0.001058289 | Down-regulated | RPS4X | 0.028188623 | Down-regulated |
| LOC728006 | 0.002849371 | Down-regulated | KRIT1 | 0.045696819 | Down-regulated |
| ZNF35 | 0.041468097 | Down-regulated | DERA | 0.004870519 | Down-regulated |
| NSDHL | 0.004871947 | Down-regulated | NDUFB10 | 0.004547718 | Down-regulated |
| ZNRD1 | 0.036178185 | Down-regulated | ABCB1 | 0.020703713 | Down-regulated |
| LOC646849 | 0.02372263 | Down-regulated | NDUFS4 | 0.000517533 | Down-regulated |
| TMED1 | 0.004613203 | Down-regulated | CCDC21 | 0.045544597 | Down-regulated |
| MRPL20 | 0.003098317 | Down-regulated | RAB35 | 0.000736964 | Down-regulated |
| BCKDK | 0.001469894 | Down-regulated | ATP5L | 0.000387797 | Down-regulated |
| LOC205251 | 0.008109525 | Down-regulated | ZSWIM1 | 0.009309792 | Down-regulated |
| HAVCR2 | 0.022876425 | Down-regulated | C6ORF136 | 0.021613705 | Down-regulated |
| HAT1 | 0.001964523 | Down-regulated | MRPS7 | 0.022707608 | Down-regulated |
| GPD1L | 0.039500832 | Down-regulated | NDUFB7 | 0.005414311 | Down-regulated |
| LOC650761 | 0.010525344 | Down-regulated | CHCHD4 | 0.000669984 | Down-regulated |
| TRIM68 | 0.006957605 | Down-regulated | LOC731985 | 0.014650995 | Down-regulated |
| PLEKHO1 | 0.001359251 | Down-regulated | NR1H3 | 0.0272392 | Down-regulated |
| RNF220 | 0.002827973 | Down-regulated | PSMB7 | 0.003069703 | Down-regulated |
| DDA1 | 0.000391 | Down-regulated | PQLC3 | 0.008499044 | Down-regulated |
| RPS29 | 0.026853585 | Down-regulated | TCF25 | 0.001423291 | Down-regulated |
| LOC642817 | 0.009840846 | Down-regulated | C6ORF153 | 0.000391196 | Down-regulated |
| HLA-DMA | 0.015949396 | Down-regulated | HS.568928 | 0.001176298 | Down-regulated |
| LOC645968 | 0.000412898 | Down-regulated | HS.282153 | 0.042302968 | Down-regulated |
| ADRB2 | 0.013075469 | Down-regulated | HS.250648 | 0.019887515 | Down-regulated |
| LOC641987 | 0.023662041 | Down-regulated | HS.551538 | 0.00418936 | Down-regulated |
| PRICKLE4 | 0.000618103 | Down-regulated | LOC401397 | 0.043816632 | Down-regulated |
| C5ORF15 | 0.018101277 | Down-regulated | HS.540121 | 0.010586482 | Down-regulated |
| SIVA | 0.006913297 | Down-regulated | HS.546375 | 0.002232433 | Down-regulated |
| POLA2 | 0.003963819 | Down-regulated | HS.572219 | 0.000100028 | Down-regulated |
| LOC441126 | 0.004394546 | Down-regulated | HS.555208 | 0.03562368 | Down-regulated |
| MED22 | 0.049078785 | Down-regulated | HS.512090 | 0.033123429 | Down-regulated |
| C20ORF27 | 0.000669984 | Down-regulated | HS.355933 | 0.01213327 | Down-regulated |
| TINF2 | 0.000109522 | Down-regulated | HS.534439 | 0.003456204 | Down-regulated |
| TPM3 | 0.000674244 | Down-regulated | MRPL37 | 0.005696074 | Down-regulated |
| NHP2L1 | 0.028344463 | Down-regulated | FANCE | 0.030897683 | Down-regulated |
| ATP6AP1 | 0.000723606 | Down-regulated | LPAR5 | 0.024348213 | Down-regulated |
| EWSR1 | 0.022061532 | Down-regulated | NCR3 | 0.022550031 | Down-regulated |
| EXOSC2 | 0.000340914 | Down-regulated | ESRRAP2 | 0.04305108 | Down-regulated |
| C3ORF38 | 0.002270761 | Down-regulated | SUGT1 | 0.03417992 | Down-regulated |
| HINT2 | 0.037214838 | Down-regulated | MRFAP1 | 0.005530952 | Down-regulated |
| ATP2A3 | 0.021460323 | Down-regulated | PSMA2 | 0.033365134 | Down-regulated |
| NSMCE1 | 0.01504808 | Down-regulated | IGFBP7 | 0.008754638 | Down-regulated |
| NCK1 | 0.000604033 | Down-regulated | AFG3L2 | 0.002097166 | Down-regulated |
| TCEA1 | 0.001993367 | Down-regulated | RRP8 | 0.001031232 | Down-regulated |
| AGPS | 0.03071558 | Down-regulated | LSM2 | 0.000604245 | Down-regulated |
| DNAJC8 | 0.000409026 | Down-regulated | HNRNPUL2 | 0.020997439 | Down-regulated |
| CMTM7 | 0.006821529 | Down-regulated | TMEM203 | 0.004524212 | Down-regulated |
| IK | 0.010588536 | Down-regulated | S1PR5 | 0.043921972 | Down-regulated |
| TPD52L2 | 0.027008771 | Down-regulated | PDIA3P | 0.007578327 | Down-regulated |
| LOC732007 | 0.017195725 | Down-regulated | C11ORF2 | 0.002870496 | Down-regulated |
| NIPSNAP3A | 0.03441018 | Down-regulated | C1ORF109 | 0.007442031 | Down-regulated |
| HMGN4 | 0.0176337 | Down-regulated | MDH2 | 0.001770925 | Down-regulated |
| RBM14 | 0.047335597 | Down-regulated | KLRB1 | 0.001015781 | Down-regulated |
| LOC728481 | 0.013987346 | Down-regulated | PMS2L5 | 0.001373569 | Down-regulated |
| LOC646567 | 0.002790945 | Down-regulated | M6PR | 0.000100028 | Down-regulated |
| SH2D1B | 0.015543874 | Down-regulated | ARPC1B | 0.03331842 | Down-regulated |
| ATP5C1 | 0.001928754 | Down-regulated | CPSF3 | 0.018158697 | Down-regulated |
| COX7A2 | 0.000695399 | Down-regulated | PSMG2 | 0.036873806 | Down-regulated |
| RPS20 | 0.027226309 | Down-regulated | ADAMTS4 | 0.019569697 | Down-regulated |
| AKR1B1 | 0.018508974 | Down-regulated | VWA3B | 0.030368669 | Down-regulated |
| LOC644063 | 0.00100656 | Down-regulated | MRPL51 | 0.021459486 | Down-regulated |
| PPP1CC | 0.004534602 | Down-regulated | NUDC | 7.91E-05 | Down-regulated |
| DLG1 | 0.009720645 | Down-regulated | UNC84B | 0.014903616 | Down-regulated |
| MFSD5 | 0.019585634 | Down-regulated | GZMB | 0.045001079 | Down-regulated |
| GLO1 | 0.01438819 | Down-regulated | UFC1 | 0.003772315 | Down-regulated |
| HDHD2 | 0.03496583 | Down-regulated | DNLZ | 0.02221977 | Down-regulated |
| APOBEC3F | 0.004508969 | Down-regulated | SEMA4C | 0.016182083 | Down-regulated |
| LYRM2 | 0.000267958 | Down-regulated | GSDM1 | 0.004521435 | Down-regulated |
| LOC653479 | 0.00899324 | Down-regulated | GPR89A | 0.020132403 | Down-regulated |
| KLHL36 | 0.002093621 | Down-regulated | NDUFB2 | 0.008195624 | Down-regulated |
| PLSCR3 | 0.022707608 | Down-regulated | FAM125A | 0.000193713 | Down-regulated |
| GLTSCR2 | 0.047891863 | Down-regulated | RBMX | 0.015745895 | Down-regulated |
| SF3B14 | 0.000326054 | Down-regulated | LOC728643 | 0.000383409 | Down-regulated |
| PTGER2 | 0.002616197 | Down-regulated | TSSC1 | 0.014851063 | Down-regulated |
| TMEM160 | 0.000731128 | Down-regulated | UTP11L | 0.013999039 | Down-regulated |
| PSMA6 | 2.86E-05 | Down-regulated | NENF | 0.04855771 | Down-regulated |
| LOC645317 | 0.000604033 | Down-regulated | NACAP1 | 0.001242633 | Down-regulated |
| NIP7 | 0.00672423 | Down-regulated | C15ORF24 | 0.025916811 | Down-regulated |
| LOC389435 | 0.012223182 | Down-regulated | ZFP36L2 | 0.012910545 | Down-regulated |
| PPIA | 0.00511389 | Down-regulated | CBWD5 | 0.018357323 | Down-regulated |
| LOC648470 | 0.022911942 | Down-regulated | ZNF821 | 0.004361557 | Down-regulated |
| VPS29 | 0.000441794 | Down-regulated | UPRT | 0.019720785 | Down-regulated |
| TCEB1 | 0.003608006 | Down-regulated | COX6B1 | 0.019926652 | Down-regulated |
| ACTG1 | 1.27E-05 | Down-regulated | IMP4 | 0.002281267 | Down-regulated |
| SF3A3 | 2.74E-05 | Down-regulated | RPL24 | 0.000814011 | Down-regulated |
| KLHL22 | 3.47E-05 | Down-regulated | LOC389286 | 0.028480781 | Down-regulated |
| RDH14 | 0.047091225 | Down-regulated | NDUFA11 | 0.001797833 | Down-regulated |
| TIGD5 | 0.005770547 | Down-regulated | HNRPR | 0.006624654 | Down-regulated |
| SH2D1A | 0.015763284 | Down-regulated | CUL5 | 0.047249338 | Down-regulated |
| RPL7L1 | 0.000993462 | Down-regulated | C12ORF24 | 0.005088601 | Down-regulated |
| FAM43A | 0.00353637 | Down-regulated | MEPCE | 0.00339686 | Down-regulated |
| CS | 0.035849824 | Down-regulated | ARMET | 0.043623335 | Down-regulated |
| MRPL33 | 0.000618103 | Down-regulated | COX7B | 0.028682213 | Down-regulated |
| ABHD6 | 0.024491901 | Down-regulated | RPP21 | 0.005796807 | Down-regulated |
| WDR68 | 0.045405208 | Down-regulated | COX17 | 1.54E-05 | Down-regulated |
| UBE2D4 | 0.029992578 | Down-regulated | RPL36AL | 0.00559622 | Down-regulated |
| GEMIN6 | 0.009441048 | Down-regulated | VAMP8 | 0.003127925 | Down-regulated |
| C1ORF144 | 0.003722698 | Down-regulated | TUSC4 | 0.002668674 | Down-regulated |
| MED10 | 0.014799755 | Down-regulated | EIF3M | 0.004676604 | Down-regulated |
| CMC1 | 0.009840846 | Down-regulated | PGRMC2 | 0.042770286 | Down-regulated |
| CCDC72 | 0.000475415 | Down-regulated | NIT2 | 0.026746311 | Down-regulated |
| PLEKHF1 | 0.011556043 | Down-regulated | C17ORF61 | 0.008537808 | Down-regulated |
| LIX1L | 0.0301287 | Down-regulated | LOC134997 | 0.029176328 | Down-regulated |
| EHF | 0.029988984 | Down-regulated | GPN2 | 2.27E-06 | Down-regulated |
| C3ORF75 | 0.007255272 | Down-regulated | NDST2 | 0.004438565 | Down-regulated |
| LOC652481 | 0.004329986 | Down-regulated | DERL1 | 0.040623306 | Down-regulated |
| LOC653226 | 2.27E-06 | Down-regulated | CDC26 | 0.013859266 | Down-regulated |
| ACAT2 | 0.016945964 | Down-regulated | TRAPPC2P1 | 0.013294986 | Down-regulated |
| GNLY | 0.023109155 | Down-regulated | TMED10P | 0.001261932 | Down-regulated |
| C2ORF29 | 0.005051493 | Down-regulated | MAGOH | 0.029992578 | Down-regulated |
| EXOSC3 | 0.003663053 | Down-regulated | LSM1 | 0.000209915 | Down-regulated |
| GAR1 | 0.049235859 | Down-regulated | C5ORF37 | 0.049101542 | Down-regulated |
| RPLP0 | 0.008735268 | Down-regulated | PTCD1 | 0.003185338 | Down-regulated |
| PSMC1 | 0.00013315 | Down-regulated | CCDC65 | 0.024589606 | Down-regulated |
| GZMK | 0.000253811 | Down-regulated | PDCD5 | 0.040541742 | Down-regulated |
| ZNF330 | 0.028480781 | Down-regulated | STARD3NL | 0.040606972 | Down-regulated |
| LOC387867 | 0.022752673 | Down-regulated | AUH | 0.007626818 | Down-regulated |
| GMDS | 0.0122618 | Down-regulated | SSRP1 | 0.036154675 | Down-regulated |
| CD96 | 0.039185677 | Down-regulated | FAM108A3 | 0.000623518 | Down-regulated |
| LOC442454 | 2.27E-06 | Down-regulated | EEF1G | 0.022064992 | Down-regulated |
| ENOPH1 | 0.006722858 | Down-regulated | TMEM150A | 0.002339815 | Down-regulated |
| ANXA2 | 0.045416696 | Down-regulated | ATP5J2 | 0.026714701 | Down-regulated |
| TMEM115 | 0.006754486 | Down-regulated | UBA3 | 0.018654158 | Down-regulated |
| ANKRD46 | 0.039434877 | Down-regulated | PTGES2 | 0.016013382 | Down-regulated |
| DDX50 | 0.030897683 | Down-regulated | BEX4 | 0.028480781 | Down-regulated |
| CUTA | 0.026644059 | Down-regulated | HSD17B10 | 0.012489087 | Down-regulated |
| RPS27L | 0.042664882 | Down-regulated | TMEM231 | 0.039754664 | Down-regulated |
| ABHD8 | 0.00353637 | Down-regulated | PRDX3 | 0.003152173 | Down-regulated |
| HADHA | 0.033106426 | Down-regulated | SNHG10 | 0.044155622 | Down-regulated |
| LRRC41 | 6.59E-05 | Down-regulated | LOC100130919 | 0.002682636 | Down-regulated |
| YIF1A | 0.004593062 | Down-regulated | LOC100129585 | 0.002682636 | Down-regulated |
| RPL27A | 0.000442312 | Down-regulated | C21ORF126 | 0.031704731 | Down-regulated |
| AKR1C3 | 0.034391147 | Down-regulated | LOC100129379 | 0.007930181 | Down-regulated |
| C12ORF41 | 0.009309792 | Down-regulated | LOC100130624 | 0.019132913 | Down-regulated |
| RPL13A | 0.024003511 | Down-regulated | C21ORF30 | 0.03718362 | Down-regulated |
| LOC441876 | 0.005849326 | Down-regulated | LOC100130135 | 0.041684758 | Down-regulated |
| ZNF671 | 0.003645188 | Down-regulated | LOC100128086 | 0.007486951 | Down-regulated |
| TSNAX | 6.28E-05 | Down-regulated | LOC100129982 | 0.042094567 | Down-regulated |
| UBAC2 | 0.000336489 | Down-regulated | LOC100128060 | 0.005724532 | Down-regulated |
| MRPS12 | 0.040793326 | Down-regulated | LOC100130291 | 0.002555415 | Down-regulated |
| CAMLG | 0.006624654 | Down-regulated | LOC100129657 | 0.037628335 | Down-regulated |
| TOMM22 | 0.007484549 | Down-regulated | LOC400013 | 0.038307118 | Down-regulated |
| GPR174 | 0.039227081 | Down-regulated | LOC645430 | 0.018452266 | Down-regulated |
| BOP1 | 0.003232404 | Down-regulated | LOC389404 | 0.005255667 | Down-regulated |
| ZNF784 | 0.045771554 | Down-regulated | LOC649553 | 0.014784692 | Down-regulated |
| PMVK | 0.001028368 | Down-regulated | LOC442232 | 0.002814846 | Down-regulated |
| LOC642210 | 0.004128034 | Down-regulated | LOC728128 | 0.040417913 | Down-regulated |
| NSUN4 | 0.010052509 | Down-regulated | LOC392285 | 0.034554698 | Down-regulated |
| ARMCX6 | 0.014228917 | Down-regulated | LOC100132795 | 0.001098791 | Down-regulated |
| LOC342892 | 0.009461129 | Down-regulated | LOC643779 | 0.035704105 | Down-regulated |
| C6ORF64 | 0.014491377 | Down-regulated | LOC100131859 | 0.049988858 | Down-regulated |
| FAM44B | 0.008499044 | Down-regulated | LOC644563 | 0.000428656 | Down-regulated |
| MED27 | 0.00194121 | Down-regulated | LOC648294 | 0.019975455 | Down-regulated |
| NFKBIE | 0.000449747 | Down-regulated | LOC100134189 | 0.034715732 | Down-regulated |
| LIPT1 | 0.003372137 | Down-regulated | LOC100132797 | 0.006342684 | Down-regulated |
| SYT11 | 0.033410636 | Down-regulated | LOC644315 | 0.006181111 | Down-regulated |
| MTIF3 | 0.036104467 | Down-regulated | LOC645969 | 0.0056276 | Down-regulated |
| C7ORF50 | 0.000582117 | Down-regulated | LOC646909 | 0.023239646 | Down-regulated |
| HPS6 | 5.76E-06 | Down-regulated | LOC100131166 | 0.040552787 | Down-regulated |
| ARHGAP17 | 0.002668674 | Down-regulated | LOC648822 | 0.003388824 | Down-regulated |
| TSPAN32 | 0.044275587 | Down-regulated | LOC100132715 | 0.012489087 | Down-regulated |
| ETFA | 0.021885357 | Down-regulated | LOC100131810 | 0.038195 | Down-regulated |
| SERPINB8 | 0.0458385 | Down-regulated | LOC645693 | 0.001076795 | Down-regulated |
| LSM3 | 0.045312412 | Down-regulated | LOC727803 | 0.00644021 | Down-regulated |
| FBL | 0.013681512 | Down-regulated | LOC728139 | 0.041866252 | Down-regulated |
| CKS1B | 0.014903616 | Down-regulated | LOC402112 | 0.003625299 | Down-regulated |
| TMED3 | 0.005917823 | Down-regulated | LOC439953 | 0.009396522 | Down-regulated |
| MAF | 0.045771554 | Down-regulated | LOC644877 | 0.002138207 | Down-regulated |
| LOC644762 | 0.02037522 | Down-regulated | LOC128192 | 0.013987346 | Down-regulated |
| CCT6A | 0.031942542 | Down-regulated | LOC644353 | 0.027778913 | Down-regulated |
| SLC27A3 | 0.006795149 | Down-regulated | LOC100132761 | 2.04E-05 | Down-regulated |
| HADH | 0.019608605 | Down-regulated | LOC100131609 | 0.004040549 | Down-regulated |
| RCC2 | 0.009088376 | Down-regulated | LOC646949 | 0.026644059 | Down-regulated |
| E4F1 | 0.001257295 | Down-regulated | LOC646294 | 0.018159385 | Down-regulated |
| LOC645385 | 0.000600342 | Down-regulated | LOC345645 | 0.008499044 | Down-regulated |
| LOC653566 | 0.009257142 | Down-regulated | LOC645387 | 0.001092629 | Down-regulated |
| DECR1 | 0.010425582 | Down-regulated | LOC647081 | 0.013018361 | Down-regulated |
| TOMM34 | 0.046210429 | Down-regulated | LOC728658 | 0.003179044 | Down-regulated |
| RANBP1 | 0.026954165 | Down-regulated | RPS6P1 | 0.009269899 | Down-regulated |
| LOC643668 | 0.000456333 | Down-regulated | LOC728666 | 0.03258048 | Down-regulated |
| IDH3B | 0.021436045 | Down-regulated | LOC728748 | 0.044157869 | Down-regulated |
| CARD11 | 0.005907494 | Down-regulated | PSMG4 | 0.049791316 | Down-regulated |
| SAFB | 0.000263962 | Down-regulated | LOC729903 | 0.030605991 | Down-regulated |
| APRT | 0.041890653 | Down-regulated | LOC729742 | 0.003737433 | Down-regulated |
| MRPS11 | 0.005512643 | Down-regulated | RPL14L | 0.0126802 | Down-regulated |
| ZCCHC9 | 0.042664882 | Down-regulated | RPS10P3 | 0.010459215 | Down-regulated |
| HACL1 | 0.008930307 | Down-regulated | LOC728368 | 0.004593062 | Down-regulated |
| PIGC | 0.00306567 | Down-regulated | LOC728698 | 0.000275594 | Down-regulated |
| MRPS6 | 0.007797281 | Down-regulated | LOC728620 | 6.78E-05 | Down-regulated |
| TARP | 0.008942649 | Down-regulated | RPL12P6 | 0.01566825 | Down-regulated |
| SLC2A8 | 0.003696147 | Down-regulated | LOC730074 | 0.006164548 | Down-regulated |
| TSPAN31 | 0.002792944 | Down-regulated | LOC729646 | 0.007894861 | Down-regulated |
| EEF1E1 | 0.007441475 | Down-regulated | LOC729259 | 0.023287024 | Down-regulated |
| FAM50A | 2.58E-05 | Down-regulated | LOC729423 | 0.03470218 | Down-regulated |
| INTS12 | 0.040574235 | Down-regulated | LOC728576 | 0.047249338 | Down-regulated |
| TBCE | 0.007290427 | Down-regulated | LOC728324 | 0.001116203 | Down-regulated |
| B2M | 0.039662147 | Down-regulated | LOC729500 | 0.001015781 | Down-regulated |
| SUMO3 | 0.013793319 | Down-regulated | LOC390557 | 0.000406109 | Down-regulated |
| PAK1IP1 | 0.040541742 | Down-regulated | LOC645296 | 0.001839715 | Down-regulated |
| TBCA | 0.008619072 | Down-regulated | TOMM6 | 0.018763773 | Down-regulated |
| AMY1B | 0.01733105 | Down-regulated | MUL1 | 0.000875491 | Down-regulated |
| RPL14 | 0.035447434 | Down-regulated | RADIL | 0.036049873 | Down-regulated |
| ZNF182 | 0.026349196 | Down-regulated | LOC728835 | 0.027627965 | Down-regulated |
| LOC648638 | 0.004040549 | Down-regulated | C1ORF174 | 6.28E-05 | Down-regulated |
| DDX46 | 0.007813751 | Down-regulated | PPIAL4A | 0.023239646 | Down-regulated |
| HDAC1 | 0.015643633 | Down-regulated | LOC100129086 | 1.47E-05 | Down-regulated |
| FEZ1 | 0.024491901 | Down-regulated | ROBLD3 | 0.019192057 | Down-regulated |
| TNF | 0.004658469 | Down-regulated | PTPMT1 | 0.004062509 | Down-regulated |
| C20ORF45 | 0.040606972 | Down-regulated | FAM32A | 0.001649317 | Down-regulated |
| MED29 | 0.003362329 | Down-regulated | COX7A2L | 0.039399731 | Down-regulated |
| CLCN4 | 0.021916875 | Down-regulated | BTBD2 | 0.00228162 | Down-regulated |
| GHITM | 0.004726707 | Down-regulated | EIF3CL | 0.000391 | Down-regulated |
| COPZ1 | 0.023425285 | Down-regulated | SDHAF2 | 0.000157151 | Down-regulated |
| PMPCB | 0.039500832 | Down-regulated | FAM165B | 0.023884043 | Down-regulated |
| ZNF816A | 0.021210861 | Down-regulated | USP17 | 0.044278357 | Down-regulated |
| PAK4 | 0.049576438 | Down-regulated | LOC645332 | 0.031404126 | Down-regulated |
| SCO1 | 0.00197109 | Down-regulated | LOC729375 | 0.043293114 | Down-regulated |
| RPL9 | 0.009309792 | Down-regulated | LOC644907 | 0.003362329 | Down-regulated |
| SPHK2 | 0.000675124 | Down-regulated | SNORD59B | 0.040552787 | Down-regulated |
| PEX7 | 0.015261768 | Down-regulated | LOC100130707 | 0.001330922 | Down-regulated |
| VDAC3 | 0.019171505 | Down-regulated | LOC641844 | 0.021460323 | Down-regulated |
| RNF216 | 0.00015921 | Down-regulated | LOC100133372 | 3.70E-05 | Down-regulated |
| RPUSD2 | 0.02106211 | Down-regulated | LOC641814 | 0.00027967 | Down-regulated |
| COMT | 0.000255745 | Down-regulated | EAPP | 0.00092415 | Down-regulated |
| CCDC115 | 0.047091225 | Down-regulated | NDUFB9 | 0.015636609 | Down-regulated |
| DBNDD2 | 0.046306496 | Down-regulated | TMEM14D | 0.000118737 | Down-regulated |
| TMEM205 | 0.007626818 | Down-regulated | LOC148413 | 3.46E-05 | Down-regulated |
| CCDC92 | 0.044155622 | Down-regulated | LOC728877 | 0.00679491 | Down-regulated |
| PARL | 0.018757821 | Down-regulated | LOC441089 | 0.000409026 | Down-regulated |
| RPL26 | 0.029779202 | Down-regulated | LOC729617 | 0.018242818 | Down-regulated |
| DAD1 | 0.016133368 | Down-regulated | LOC100133931 | 0.003534871 | Down-regulated |
| LOC646200 | 0.012678128 | Down-regulated | DGCR6L | 0.034391147 | Down-regulated |
| MAPKAPK3 | 0.019779801 | Down-regulated | TOMM20L | 0.02712097 | Down-regulated |
| TMEM216 | 0.020987405 | Down-regulated | LOC100133697 | 0.00221453 | Down-regulated |
| RASSF7 | 0.032884691 | Down-regulated | LOC100130553 | 0.016804399 | Down-regulated |
| PSMD8 | 0.002668674 | Down-regulated | LOC92249 | 0.042447732 | Down-regulated |
| LOC374395 | 0.029043415 | Down-regulated | GLRX3 | 0.000235827 | Down-regulated |
| PDCD6 | 0.00963745 | Down-regulated | LOC728188 | 0.006085115 | Down-regulated |
| LOC388122 | 0.00561871 | Down-regulated | LOC100132717 | 0.000205654 | Down-regulated |
| DDOST | 0.040673752 | Down-regulated | SNHG6 | 0.00082857 | Down-regulated |
| C1ORF19 | 0.013987346 | Down-regulated | C7ORF40 | 0.04082021 | Down-regulated |
| MCEE | 0.01269129 | Down-regulated | LOC100127999 | 0.04782276 | Down-regulated |
| R3HCC1 | 0.001153133 | Down-regulated | LOC100127918 | 1.54E-05 | Down-regulated |
| CDC42SE2 | 0.000601991 | Down-regulated | LOC100129759 | 0.049514383 | Down-regulated |
| BCKDHA | 0.011972065 | Down-regulated | LOC100129645 | 0.02196722 | Down-regulated |
| COPS5 | 0.029509226 | Down-regulated | LOC100130003 | 0.041133866 | Down-regulated |
| PDXP | 2.18E-05 | Down-regulated | LOC100129902 | 0.009441048 | Down-regulated |
| GNB2L1 | 0.034715732 | Down-regulated | KRT18P34 | 0.022707608 | Down-regulated |
| PHACTR4 | 0.00341337 | Down-regulated | LOC100130633 | 0.00251219 | Down-regulated |
| TXLNA | 0.019577882 | Down-regulated | LOC100129553 | 0.001976686 | Down-regulated |
| CRLS1 | 0.004489073 | Down-regulated | LOC100128353 | 0.000102006 | Down-regulated |
| NDUFA12 | 0.000675383 | Down-regulated | LOC100128899 | 0.011156961 | Down-regulated |
| GTF2B | 0.009110998 | Down-regulated | LOC727826 | 0.007611852 | Down-regulated |
| PIGU | 0.001062856 | Down-regulated | LOC392501 | 0.0458385 | Down-regulated |
| RNPEP | 0.013147846 | Down-regulated | LOC391126 | 0.012489616 | Down-regulated |
| LOC402694 | 0.005336939 | Down-regulated | LOC387930 | 0.009177721 | Down-regulated |
| NUP62 | 0.00242528 | Down-regulated | LOC100132510 | 0.048779687 | Down-regulated |
| DEXI | 0.04855771 | Down-regulated | LOC643507 | 0.004533415 | Down-regulated |
| LOC643284 | 0.006603876 | Down-regulated | LOC651697 | 0.00899324 | Down-regulated |
| PDHB | 0.005517239 | Down-regulated | LOC648771 | 0.003382483 | Down-regulated |
| GTF2H5 | 0.023109155 | Down-regulated | LOC440991 | 0.041866252 | Down-regulated |
| CRSP9 | 0.017971745 | Down-regulated | LOC100132037 | 0.006051896 | Down-regulated |
| SLC41A3 | 0.001046549 | Down-regulated | LOC728207 | 0.016466291 | Down-regulated |
| VKORC1 | 0.00061679 | Down-regulated | LOC100133390 | 0.029760124 | Down-regulated |
| DUSP11 | 0.020987405 | Down-regulated | LOC645630 | 0.003069703 | Down-regulated |
| SLC2A4RG | 0.001381744 | Down-regulated | LOC100131785 | 0.01335213 | Down-regulated |
| RPS11 | 0.003632128 | Down-regulated | LOC100132918 | 0.000723606 | Down-regulated |
| UROD | 0.046102506 | Down-regulated | LOC100132199 | 0.046103212 | Down-regulated |
| KLHDC2 | 0.006210761 | Down-regulated | LOC643733 | 0.009165572 | Down-regulated |
| ZNHIT1 | 0.038007745 | Down-regulated | LOC643863 | 0.020613466 | Down-regulated |
| DUSP28 | 0.008323013 | Down-regulated | LOC391833 | 0.049890572 | Down-regulated |
| CD160 | 0.001679402 | Down-regulated | LOC643531 | 0.01805512 | Down-regulated |
| C8ORF76 | 3.16E-06 | Down-regulated | LOC646672 | 0.033365134 | Down-regulated |
| CCNH | 0.047335597 | Down-regulated | LOC442727 | 0.015166811 | Down-regulated |
| SFRS10 | 0.021461635 | Down-regulated | LOC402175 | 0.000255745 | Down-regulated |
| PXMP3 | 0.042617943 | Down-regulated | LOC391532 | 3.46E-05 | Down-regulated |
| TBCC | 0.016613571 | Down-regulated | LOC642975 | 0.031018637 | Down-regulated |
| POMT1 | 0.015166811 | Down-regulated | LOC100131387 | 0.047335597 | Down-regulated |
| TJAP1 | 0.00197109 | Down-regulated | LOC730187 | 0.032966474 | Down-regulated |
| MIF4GD | 0.00027967 | Down-regulated | LOC728873 | 0.003149011 | Down-regulated |
| DCTN6 | 0.001903374 | Down-regulated | LOC730167 | 0.001707371 | Down-regulated |
| SDHA | 0.035757951 | Down-regulated | LOC729687 | 0.0458385 | Down-regulated |
| WDR67 | 0.001497672 | Down-regulated | LOC729102 | 0.01082311 | Down-regulated |
| NOTUM | 0.004046877 | Down-regulated | MIR652 | 0.033030308 | Down-regulated |
| PARK7 | 0.022707608 | Down-regulated | SNORD113-1 | 0.040646868 | Down-regulated |
| MIR20B | 0.047335597 | Down-regulated | LOC100132112 | 0.016484525 | up-regulated |
| LPP | 0.000810332 | up-regulated | UBE4A | 0.000517533 | up-regulated |
| LOC645349 | 0.034296896 | up-regulated | USP34 | 0.024491901 | up-regulated |
| MFN2 | 0.000306978 | up-regulated | DPRXP4 | 0.04317976 | up-regulated |
| RTN3 | 0.022707608 | up-regulated | ROCK1 | 0.003924007 | up-regulated |
| STK10 | 0.023229601 | up-regulated | PPP1R12A | 0.008323013 | up-regulated |
| MKL1 | 0.008754638 | up-regulated | RAI1 | 0.030897683 | up-regulated |
| FHOD1 | 0.043921972 | up-regulated | C15ORF29 | 0.046316854 | up-regulated |
| MAP3K11 | 0.002052843 | up-regulated | PCNX | 0.013793319 | up-regulated |
| BASP1 | 0.003092394 | up-regulated | C14ORF102 | 0.001007016 | up-regulated |
| RPS6KA3 | 0.004262661 | up-regulated | CEP164 | 0.029043415 | up-regulated |
| ERF | 0.007060121 | up-regulated | RAB11FIP2 | 0.031883113 | up-regulated |
| PITPNM1 | 0.006806159 | up-regulated | UBR5 | 0.037330939 | up-regulated |
| DNAJB14 | 0.002079023 | up-regulated | DCTN4 | 0.011972065 | up-regulated |
| C6ORF62 | 0.001424292 | up-regulated | SERINC1 | 0.003185338 | up-regulated |
| MKNK2 | 0.003226236 | up-regulated | LOC441087 | 0.044089059 | up-regulated |
| KHNYN | 0.007338024 | up-regulated | CEP350 | 5.90E-05 | up-regulated |
| FCHO1 | 0.03566587 | up-regulated | LOC651309 | 0.003378824 | up-regulated |
| SLCO3A1 | 0.03075231 | up-regulated | TAPBP | 0.000235827 | up-regulated |
| LSP1 | 0.041942442 | up-regulated | GRB2 | 0.039651325 | up-regulated |
| LENG8 | 0.003625299 | up-regulated | MKLN1 | 0.001330922 | up-regulated |
| SMEK1 | 0.018603481 | up-regulated | SPATA13 | 0.004097273 | up-regulated |
| ZBTB34 | 0.023214369 | up-regulated | NXNL1 | 0.039609671 | up-regulated |
| TRIM27 | 0.00868839 | up-regulated | PTGFRN | 0.011955536 | up-regulated |
| STAU1 | 0.001853427 | up-regulated | BAT2D1 | 0.035284115 | up-regulated |
| GLT1D1 | 0.0267602 | up-regulated | MICALCL | 0.020080349 | up-regulated |
| C16ORF59 | 0.027042051 | up-regulated | LOC653829 | 0.027599865 | up-regulated |
| OR14A16 | 0.01504808 | up-regulated | VASP | 0.034668453 | up-regulated |
| DNM2 | 0.033365134 | up-regulated | MGC18216 | 0.000674244 | up-regulated |
| LIN37 | 0.037923818 | up-regulated | EP300 | 0.000675383 | up-regulated |
| STARD3 | 0.000263962 | up-regulated | ZNFX1 | 3.46E-05 | up-regulated |
| TSNAXIP1 | 0.046306496 | up-regulated | FLJ10081 | 0.01580325 | up-regulated |
| OR2AG2 | 0.039357166 | up-regulated | REM2 | 0.043524606 | up-regulated |
| LOC90120 | 0.026683606 | up-regulated | BBX | 0.014880636 | up-regulated |
| TMEM137 | 0.035659558 | up-regulated | MGRN1 | 0.008894676 | up-regulated |
| CRTC2 | 0.01791551 | up-regulated | KIAA0492 | 0.007553465 | up-regulated |
| LOC642035 | 0.025419298 | up-regulated | CORO1C | 0.026811148 | up-regulated |
| BAZ1A | 0.00825571 | up-regulated | MBD6 | 0.013530748 | up-regulated |
| CHD4 | 0.01805237 | up-regulated | EVI5 | 0.028966746 | up-regulated |
| C10ORF26 | 0.003221923 | up-regulated | LOC729843 | 0.000252753 | up-regulated |
| GNB2 | 0.003651729 | up-regulated | TMEM184B | 0.000190715 | up-regulated |
| PPP1R10 | 0.000263962 | up-regulated | ALDOB | 0.000731128 | up-regulated |
| ROCK2 | 0.001015781 | up-regulated | LHFPL2 | 0.029377639 | up-regulated |
| LOC653158 | 0.003278221 | up-regulated | PIM2 | 6.59E-05 | up-regulated |
| MSN | 0.000590959 | up-regulated | CXCL14 | 0.001514103 | up-regulated |
| RNF44 | 0.005497285 | up-regulated | CCS | 0.011833404 | up-regulated |
| LOC401622 | 0.014333184 | up-regulated | S100A12 | 0.043387289 | up-regulated |
| NBEAL2 | 0.00168398 | up-regulated | ATG10 | 0.004670161 | up-regulated |
| SPTBN1 | 0.016484525 | up-regulated | RBM20 | 0.006193622 | up-regulated |
| Septin 6 | 0.013793319 | up-regulated | DMTF1 | 2.86E-05 | up-regulated |
| KIAA0408 | 0.030270457 | up-regulated | LBA1 | 0.000562939 | up-regulated |
| DIAPH1 | 0.014571173 | up-regulated | C17ORF62 | 0.000391 | up-regulated |
| ZMYM2 | 0.015664157 | up-regulated | GSTM5 | 0.021663899 | up-regulated |
| SMEK2 | 0.041015136 | up-regulated | ZFP42 | 0.013631203 | up-regulated |
| CNNM3 | 0.012313781 | up-regulated | ITM2B | 0.028348748 | up-regulated |
| CXCR1 | 0.035987389 | up-regulated | UBE3B | 0.009950861 | up-regulated |
| EDG4 | 0.016527398 | up-regulated | SH2B3 | 0.000442312 | up-regulated |
| GPSM3 | 0.005733314 | up-regulated | TMEM55A | 0.009532568 | up-regulated |
| CPPED1 | 0.040358837 | up-regulated | SLC35E1 | 0.004403758 | up-regulated |
| SON | 0.006773102 | up-regulated | DOCK5 | 0.021436045 | up-regulated |
| UBE2B | 0.04927015 | up-regulated | IFNAR1 | 0.031876469 | up-regulated |
| EPHA10 | 0.033212697 | up-regulated | KIAA1600 | 0.000136978 | up-regulated |
| STAT3 | 0.029466978 | up-regulated | WNK1 | 0.002097109 | up-regulated |
| OR1S2 | 0.039434877 | up-regulated | LOC221442 | 0.000715172 | up-regulated |
| CANT1 | 3.03E-05 | up-regulated | KIAA0556 | 0.040214723 | up-regulated |
| LOC644590 | 0.010403531 | up-regulated | SAMD4B | 0.003069703 | up-regulated |
| DHTKD1 | 0.007578327 | up-regulated | STAT5A | 0.000100028 | up-regulated |
| TP53BP1 | 0.013793249 | up-regulated | PRKD2 | 8.35E-05 | up-regulated |
| INPP5A | 0.023749461 | up-regulated | LOC643738 | 0.0013204 | up-regulated |
| ATG16L2 | 0.033722531 | up-regulated | XPO6 | 0.018802827 | up-regulated |
| SPG11 | 0.000562939 | up-regulated | DIP2B | 0.001814179 | up-regulated |
| SH3BP5L | 0.005060423 | up-regulated | INTS3 | 0.000386424 | up-regulated |
| USP15 | 0.005856828 | up-regulated | MFSD11 | 0.000391196 | up-regulated |
| CLPTM1 | 0.028060859 | up-regulated | LOC647691 | 0.029509226 | up-regulated |
| RNF149 | 0.021006136 | up-regulated | LOC642489 | 9.59E-07 | up-regulated |
| C14ORF106 | 0.000590959 | up-regulated | 6-Mar | 0.030897683 | up-regulated |
| TRIM56 | 0.003663053 | up-regulated | TMEM88 | 0.000623518 | up-regulated |
| STK3 | 0.032488549 | up-regulated | TAOK1 | 0.000274815 | up-regulated |
| LPPR2 | 0.003069703 | up-regulated | CYGB | 0.041866252 | up-regulated |
| SLA | 0.008906378 | up-regulated | TRAFD1 | 0.004349861 | up-regulated |
| IFRD1 | 0.046306496 | up-regulated | TTYH1 | 0.008499044 | up-regulated |
| C1ORF24 | 0.015316297 | up-regulated | P2RX1 | 0.03572862 | up-regulated |
| ZDHHC18 | 0.000503273 | up-regulated | RNF31 | 0.023884043 | up-regulated |
| WASPIP | 0.010459215 | up-regulated | GNA13 | 0.00221453 | up-regulated |
| GVIN1 | 0.00353637 | up-regulated | NADK | 0.004438565 | up-regulated |
| KIAA0913 | 0.005700028 | up-regulated | INTS8 | 0.002138207 | up-regulated |
| GLRB | 0.045771554 | up-regulated | LOC644144 | 0.030459954 | up-regulated |
| LOC440345 | 0.026273976 | up-regulated | DPP8 | 0.018067621 | up-regulated |
| DCP2 | 0.004721189 | up-regulated | SORL1 | 1.50E-05 | up-regulated |
| LPIN2 | 0.000656532 | up-regulated | VAX2 | 0.008569264 | up-regulated |
| NRBP1 | 0.002232433 | up-regulated | ADRM1 | 0.044145153 | up-regulated |
| ARID3A | 0.004138708 | up-regulated | RNF160 | 0.015316297 | up-regulated |
| SERPING1 | 0.022707608 | up-regulated | BAT2 | 0.00025444 | up-regulated |
| FCHO2 | 0.043495374 | up-regulated | ZNF75D | 0.040646868 | up-regulated |
| GALNT3 | 0.013294986 | up-regulated | EIF4G2 | 0.003885843 | up-regulated |
| GAPVD1 | 0.020080349 | up-regulated | SLC40A1 | 0.0187227 | up-regulated |
| CACNA1H | 0.049023401 | up-regulated | C4ORF29 | 0.028356058 | up-regulated |
| HOMER2 | 0.032840773 | up-regulated | HELZ | 8.93E-05 | up-regulated |
| HSD17B7 | 0.005497285 | up-regulated | STAT6 | 0.032083814 | up-regulated |
| MLF2 | 0.0053565 | up-regulated | PIK3CB | 0.041089251 | up-regulated |
| LOC650128 | 0.01504808 | up-regulated | MTF1 | 5.99E-05 | up-regulated |
| SMPD2 | 0.042408385 | up-regulated | ANPEP | 0.001007016 | up-regulated |
| MAP3K7IP2 | 1.72E-06 | up-regulated | MAGMAS | 0.014650995 | up-regulated |
| MYO9B | 0.002248212 | up-regulated | TECPR2 | 0.016482457 | up-regulated |
| SEMA4B | 0.002918198 | up-regulated | ZNF384 | 0.005796807 | up-regulated |
| PKM2 | 0.000809464 | up-regulated | HEATR4 | 0.015636609 | up-regulated |
| CCDC109A | 0.042770286 | up-regulated | NUFIP2 | 0.02316296 | up-regulated |
| SSH2 | 0.026683606 | up-regulated | DOCK11 | 0.040804245 | up-regulated |
| LOC440704 | 0.037996121 | up-regulated | ABCA1 | 0.044360791 | up-regulated |
| SLC6A6 | 0.02712097 | up-regulated | REL | 0.03219496 | up-regulated |
| FLJ35848 | 0.037734861 | up-regulated | PLEKHF2 | 0.001251715 | up-regulated |
| LOC440341 | 0.00353637 | up-regulated | LARP4B | 0.000151888 | up-regulated |
| CTSD | 0.014228917 | up-regulated | PIK3CD | 0.009088376 | up-regulated |
| FLJ42291 | 0.035618748 | up-regulated | GATAD2B | 2.18E-05 | up-regulated |
| TM9SF4 | 0.013218493 | up-regulated | TUBGCP6 | 0.007375457 | up-regulated |
| LOC652578 | 0.016528111 | up-regulated | MFAP4 | 0.042770286 | up-regulated |
| C19ORF35 | 0.001898897 | up-regulated | PAK1 | 0.048810498 | up-regulated |
| DDX17 | 0.015567266 | up-regulated | SLC12A6 | 0.009263503 | up-regulated |
| UBE4B | 0.005917823 | up-regulated | RPGR | 0.001679402 | up-regulated |
| KPNA1 | 0.001559463 | up-regulated | LOC728888 | 0.009540068 | up-regulated |
| C1ORF152 | 0.008886918 | up-regulated | DLG4 | 0.022876425 | up-regulated |
| MTMR3 | 0.049078785 | up-regulated | CAPZA2 | 0.003166052 | up-regulated |
| LOC440776 | 0.009117523 | up-regulated | CCDC7 | 0.039754664 | up-regulated |
| DISC1 | 0.029093171 | up-regulated | CDC42SE1 | 0.000412898 | up-regulated |
| C20ORF95 | 0.03496583 | up-regulated | SETD2 | 0.007611852 | up-regulated |
| CHD7 | 0.01791551 | up-regulated | RAB5C | 0.00092415 | up-regulated |
| PTGS2 | 0.048389939 | up-regulated | C7ORF43 | 0.001007016 | up-regulated |
| TARDBP | 0.009378419 | up-regulated | HECA | 0.000407925 | up-regulated |
| LOC648852 | 0.022707608 | up-regulated | AKNA | 0.02743255 | up-regulated |
| TRIM21 | 0.000111153 | up-regulated | BNIP2 | 0.028480781 | up-regulated |
| KIAA1267 | 0.000592965 | up-regulated | HAUS4 | 0.040673752 | up-regulated |
| VPS8 | 0.024748943 | up-regulated | MTMR4 | 3.37E-05 | up-regulated |
| RHOT1 | 0.03417992 | up-regulated | DNAJC30 | 0.026170934 | up-regulated |
| LOC653867 | 0.011720808 | up-regulated | ABCD1 | 0.0458385 | up-regulated |
| TAF15 | 0.016761795 | up-regulated | ARHGEF1 | 0.005011089 | up-regulated |
| BCYRN1 | 0.025139071 | up-regulated | LOC143188 | 0.030605991 | up-regulated |
| HERC4 | 0.007256062 | up-regulated | CHPF2 | 0.007619357 | up-regulated |
| KIDINS220 | 0.000637614 | up-regulated | UNC13D | 0.00181839 | up-regulated |
| PELI1 | 0.04890509 | up-regulated | C13ORF18 | 0.009840846 | up-regulated |
| C3ORF62 | 0.009178629 | up-regulated | C16ORF72 | 0.047249338 | up-regulated |
| LOC729012 | 0.048194431 | up-regulated | LOC645052 | 0.018016867 | up-regulated |
| AGPAT1 | 0.000111153 | up-regulated | CALCOCO1 | 0.04621173 | up-regulated |
| LOC728519 | 0.048810466 | up-regulated | NAB1 | 0.036816789 | up-regulated |
| APOBEC3A | 0.048599907 | up-regulated | MMP14 | 0.032489773 | up-regulated |
| MAPK8IP1 | 0.026663506 | up-regulated | FLJ12078 | 0.030642161 | up-regulated |
| LOC730994 | 0.015532938 | up-regulated | FAM100B | 0.008813325 | up-regulated |
| BAZ1B | 0.031515656 | up-regulated | EIF4G3 | 0.001107733 | up-regulated |
| MYO1F | 0.002041524 | up-regulated | GNAI2 | 0.002504102 | up-regulated |
| PAN3 | 2.27E-06 | up-regulated | LRRK2 | 0.026284311 | up-regulated |
| TMSB15A | 0.003098317 | up-regulated | C5ORF41 | 2.74E-05 | up-regulated |
| GRK6 | 0.007797281 | up-regulated | ZSWIM6 | 0.004885264 | up-regulated |
| RNF19B | 0.000306978 | up-regulated | C16ORF56 | 0.001829367 | up-regulated |
| NT5C2 | 0.031573899 | up-regulated | PREX1 | 0.001015904 | up-regulated |
| VCPIP1 | 0.000562939 | up-regulated | EXTL3 | 0.016732111 | up-regulated |
| CYBB | 0.044155622 | up-regulated | CD44 | 0.038169784 | up-regulated |
| TRIM33 | 0.014857746 | up-regulated | FAM111A | 6.28E-05 | up-regulated |
| MASTL | 0.02013585 | up-regulated | NFIB | 0.00925529 | up-regulated |
| ARF3 | 0.007611852 | up-regulated | FAM126B | 0.00794827 | up-regulated |
| ZNF281 | 0.000425754 | up-regulated | ZCCHC6 | 0.000551283 | up-regulated |
| CCNL1 | 0.002668674 | up-regulated | EDEM1 | 4.40E-05 | up-regulated |
| TMEM154 | 0.005257245 | up-regulated | TBC1D14 | 0.001114475 | up-regulated |
| ACSL4 | 0.009088376 | up-regulated | PELI2 | 0.007972082 | up-regulated |
| UBE1 | 2.13E-05 | up-regulated | KCNJ2 | 0.005011089 | up-regulated |
| FKBP1A | 1.45E-05 | up-regulated | MYO18A | 0.047477571 | up-regulated |
| LHX6 | 0.045771554 | up-regulated | LYN | 0.034707656 | up-regulated |
| TNPO3 | 0.03970975 | up-regulated | KIAA1009 | 0.047980079 | up-regulated |
| MED13L | 0.00083043 | up-regulated | PPP1R3D | 0.024280832 | up-regulated |
| STAT5B | 4.66E-05 | up-regulated | PGLYRP4 | 0.03071558 | up-regulated |
| WASF2 | 0.014889095 | up-regulated | SMAP2 | 0.005917823 | up-regulated |
| PXN | 0.001839068 | up-regulated | BRI3 | 0.005917423 | up-regulated |
| ACSL1 | 0.016527398 | up-regulated | CEBPD | 0.048209743 | up-regulated |
| TNFRSF1A | 0.019344632 | up-regulated | KIAA1033 | 0.007960071 | up-regulated |
| ITGAM | 0.00221453 | up-regulated | POLR2A | 3.16E-06 | up-regulated |
| SLC22A4 | 0.026284311 | up-regulated | OSBPL8 | 0.009646346 | up-regulated |
| SLU7 | 0.035849824 | up-regulated | STAG2 | 0.006166128 | up-regulated |
| UBN2 | 0.000981742 | up-regulated | LOC153684 | 0.004083116 | up-regulated |
| MAGEA11 | 0.049216239 | up-regulated | PCNXL3 | 0.010586482 | up-regulated |
| THBS1 | 0.010109324 | up-regulated | UBP1 | 0.028685124 | up-regulated |
| CEP152 | 0.010063364 | up-regulated | ZNF12 | 0.008499044 | up-regulated |
| BAT1 | 0.000534591 | up-regulated | S1PR4 | 0.006200538 | up-regulated |
| C15ORF28 | 0.010764159 | up-regulated | CIC | 0.04682981 | up-regulated |
| MGEA5 | 0.015901916 | up-regulated | DENND5A | 0.000298682 | up-regulated |
| PCBP3 | 0.040737709 | up-regulated | BRD3 | 0.007910337 | up-regulated |
| RXRA | 0.013669112 | up-regulated | LMOD3 | 0.003166052 | up-regulated |
| FLNA | 0.017523134 | up-regulated | C1QC | 0.040606601 | up-regulated |
| SEMA4D | 6.36E-05 | up-regulated | HERC1 | 0.00197109 | up-regulated |
| UPF1 | 0.016981771 | up-regulated | CLK2 | 0.025646921 | up-regulated |
| TBC1D4 | 0.010586482 | up-regulated | DAAM1 | 0.023292886 | up-regulated |
| ZEB2 | 0.014161486 | up-regulated | CENPO | 0.023306755 | up-regulated |
| PJA2 | 0.023236046 | up-regulated | PCGF5 | 0.021352865 | up-regulated |
| AAK1 | 0.006070238 | up-regulated | HEY1 | 0.040062174 | up-regulated |
| SFRS14 | 0.008955866 | up-regulated | SNX19 | 0.025713609 | up-regulated |
| FLJ20273 | 0.002682636 | up-regulated | LOC652755 | 1.54E-05 | up-regulated |
| CCNK | 0.013656711 | up-regulated | COTL1 | 0.02372263 | up-regulated |
| FAM63A | 0.016527398 | up-regulated | SIN3B | 0.022752673 | up-regulated |
| LCOR | 0.001046549 | up-regulated | WBP2 | 0.016969337 | up-regulated |
| C5AR1 | 0.003773796 | up-regulated | PHIP | 0.003608006 | up-regulated |
| ANKRD13A | 0.004438565 | up-regulated | DOCK8 | 0.00015268 | up-regulated |
| PLEKHO2 | 0.037923818 | up-regulated | LOC646043 | 0.010956998 | up-regulated |
| CDC42BPB | 0.039500832 | up-regulated | ITPR1 | 0.006468541 | up-regulated |
| LOC284672 | 0.000340487 | up-regulated | DNAJC5 | 0.036690892 | up-regulated |
| PCM1 | 0.003663053 | up-regulated | NUAK2 | 0.040226785 | up-regulated |
| SLC9A8 | 0.001007016 | up-regulated | CFLAR | 0.002041524 | up-regulated |
| SP100 | 0.021978233 | up-regulated | SLC30A7 | 0.030990922 | up-regulated |
| ASAP1 | 0.035212575 | up-regulated | FURIN | 0.017450744 | up-regulated |
| ELF2 | 0.039443542 | up-regulated | XPC | 0.000431198 | up-regulated |
| TAGLN2 | 0.002021312 | up-regulated | NFE2L2 | 0.015240124 | up-regulated |
| ZNF14 | 0.028561527 | up-regulated | HUWE1 | 0.001839068 | up-regulated |
| DOK3 | 0.019722669 | up-regulated | MTPN | 0.00740403 | up-regulated |
| EPHB1 | 0.000441794 | up-regulated | C10ORF76 | 0.03331842 | up-regulated |
| SMARCD2 | 0.005504715 | up-regulated | MNT | 0.035308912 | up-regulated |
| TTYH3 | 0.022406946 | up-regulated | ZHX2 | 0.011169765 | up-regulated |
| ZC3H7A | 0.009257142 | up-regulated | MED12 | 0.014376512 | up-regulated |
| TMEM19 | 0.009460904 | up-regulated | PISD | 0.005856828 | up-regulated |
| P4HA1 | 0.035187626 | up-regulated | FBXL3 | 0.001084723 | up-regulated |
| C16ORF7 | 0.043013106 | up-regulated | SNRK | 0.000391 | up-regulated |
| NFIX | 0.008323013 | up-regulated | FGR | 0.039519444 | up-regulated |
| SMARCC1 | 0.035177424 | up-regulated | ERCC5 | 0.003128198 | up-regulated |
| FLJ11292 | 0.029509226 | up-regulated | LOC644935 | 0.012910545 | up-regulated |
| NCOA6 | 0.03417992 | up-regulated | CORO7 | 0.000665777 | up-regulated |
| PARVG | 0.011359431 | up-regulated | LOC642678 | 0.008763802 | up-regulated |
| CLK4 | 0.006398539 | up-regulated | KIAA1012 | 0.010656331 | up-regulated |
| KPNA6 | 0.000604033 | up-regulated | GRINA | 0.013793249 | up-regulated |
| LOC642333 | 0.002743384 | up-regulated | FYB | 0.000194466 | up-regulated |
| KIAA0240 | 0.001215702 | up-regulated | NLRC4 | 0.003166052 | up-regulated |
| PYGL | 0.016751735 | up-regulated | SNX13 | 0.016484525 | up-regulated |
| CTSB | 0.026444758 | up-regulated | C3ORF58 | 0.025919596 | up-regulated |
| IL6R | 0.020714383 | up-regulated | CHERP | 0.032142922 | up-regulated |
| SPI1 | 0.01071651 | up-regulated | JARID1C | 0.023884043 | up-regulated |
| TLN1 | 0.006051896 | up-regulated | KIAA1468 | 0.039609671 | up-regulated |
| LOC613037 | 0.001370725 | up-regulated | PAPOLA | 0.000269279 | up-regulated |
| PDZD8 | 0.002303895 | up-regulated | PCF11 | 0.007375457 | up-regulated |
| SASH3 | 0.009859257 | up-regulated | SRPK1 | 0.001169581 | up-regulated |
| G6PD | 0.001514103 | up-regulated | SRBD1 | 0.045389517 | up-regulated |
| OGT | 0.035308912 | up-regulated | CAMTA2 | 0.015578271 | up-regulated |
| TRIM38 | 0.001022449 | up-regulated | NFKB2 | 0.003333727 | up-regulated |
| SLC19A1 | 0.002048017 | up-regulated | TRY1 | 0.041133866 | up-regulated |
| ENDOD1 | 0.036967413 | up-regulated | PRPF38B | 0.005859703 | up-regulated |
| COPB1 | 0.017898204 | up-regulated | SAMD9L | 0.021883023 | up-regulated |
| UBR4 | 0.012697837 | up-regulated | MARS | 0.04855771 | up-regulated |
| PHF21A | 0.003773796 | up-regulated | NRD1 | 0.000106918 | up-regulated |
| ARCN1 | 0.034668453 | up-regulated | ZFP106 | 0.006051896 | up-regulated |
| SP8 | 0.030897683 | up-regulated | LOC648984 | 0.039765399 | up-regulated |
| PTOV1 | 0.021804758 | up-regulated | CCDC97 | 0.001950816 | up-regulated |
| CLASP1 | 0.00083138 | up-regulated | LOC647655 | 0.03897118 | up-regulated |
| RNF135 | 0.010540937 | up-regulated | APBB1IP | 5.76E-06 | up-regulated |
| SLK | 0.039293994 | up-regulated | RERE | 0.000425754 | up-regulated |
| LOC642684 | 0.032783762 | up-regulated | LOC654103 | 0.036949775 | up-regulated |
| KREMEN1 | 0.036273434 | up-regulated | MVP | 0.005512643 | up-regulated |
| RP5-1022P6.2 | 0.000244845 | up-regulated | KCTD20 | 0.001966499 | up-regulated |
| LOC399900 | 0.001058289 | up-regulated | LAT2 | 0.012619786 | up-regulated |
| SYNJ1 | 0.005231074 | up-regulated | ZFC3H1 | 0.008122902 | up-regulated |
| ZC3H3 | 0.032238583 | up-regulated | AP2A2 | 0.024797275 | up-regulated |
| SEMA4A | 0.018383501 | up-regulated | TRIB1 | 0.032142922 | up-regulated |
| LOC645958 | 0.035117914 | up-regulated | IQGAP1 | 0.000407642 | up-regulated |
| N-PAC | 0.001138074 | up-regulated | DKFZP586I1420 | 0.023109155 | up-regulated |
| FEM1C | 0.001356684 | up-regulated | C14ORF4 | 0.007984018 | up-regulated |
| LOC653136 | 0.013151732 | up-regulated | LRMP | 0.04082021 | up-regulated |
| RBM38 | 0.024003511 | up-regulated | MEFV | 0.000193713 | up-regulated |
| DHRSX | 0.004885264 | up-regulated | C7ORF53 | 0.000306978 | up-regulated |
| FAM53B | 0.008314187 | up-regulated | ITPRIP | 0.049216239 | up-regulated |
| FNDC3A | 0.019653429 | up-regulated | GMIP | 0.001457446 | up-regulated |
| NUMB | 0.001412984 | up-regulated | CPNE5 | 0.01504808 | up-regulated |
| C6ORF85 | 0.038828772 | up-regulated | ZNF700 | 0.006603876 | up-regulated |
| SAFB2 | 0.036509919 | up-regulated | FAM8A1 | 0.007630532 | up-regulated |
| NRGN | 0.009950861 | up-regulated | SPOPL | 0.039754664 | up-regulated |
| NXF1 | 0.012910545 | up-regulated | SFRS17A | 0.019177536 | up-regulated |
| NUP153 | 0.0012434 | up-regulated | MICAL1 | 0.001158177 | up-regulated |
| LONRF1 | 0.033015157 | up-regulated | SMCHD1 | 0.049315756 | up-regulated |
| HECTD1 | 0.00963745 | up-regulated | IQSEC1 | 0.000456333 | up-regulated |
| HOXA7 | 0.013999039 | up-regulated | SBNO2 | 6.04E-05 | up-regulated |
| KDM3B | 0.031117923 | up-regulated | XRN1 | 0.002285473 | up-regulated |
| PKN2 | 3.03E-05 | up-regulated | LOC729021 | 0.006681436 | up-regulated |
| PTBP2 | 0.017972966 | up-regulated | FAM49B | 0.013357031 | up-regulated |
| LOC647797 | 0.029509226 | up-regulated | TDRD1 | 0.005955774 | up-regulated |
| LOC653778 | 0.018802827 | up-regulated | JUND | 0.00561103 | up-regulated |
| PANK2 | 0.04927015 | up-regulated | DYSF | 0.00027967 | up-regulated |
| COPG | 0.04783107 | up-regulated | TLK1 | 0.008323013 | up-regulated |
| ADAM8 | 0.011021787 | up-regulated | POU2AF1 | 0.048821568 | up-regulated |
| GNL3L | 0.01676963 | up-regulated | RELB | 0.022119295 | up-regulated |
| BIN3 | 0.023672518 | up-regulated | LOC648615 | 0.043921972 | up-regulated |
| NCOA3 | 0.000157187 | up-regulated | MAPK8IP3 | 0.001702526 | up-regulated |
| CCNY | 0.006255066 | up-regulated | ARHGAP21 | 0.044155622 | up-regulated |
| F5 | 0.022773664 | up-regulated | CYLN2 | 0.005308868 | up-regulated |
| TM9SF1 | 0.017873328 | up-regulated | MYCBP2 | 0.001393609 | up-regulated |
| CHMP1A | 0.007910337 | up-regulated | ANTXR2 | 0.004349861 | up-regulated |
| RASSF2 | 0.002583063 | up-regulated | PHKA2 | 0.045346657 | up-regulated |
| VPS37B | 0.021302809 | up-regulated | UBAP2L | 0.000102006 | up-regulated |
| APLP2 | 0.023109155 | up-regulated | MAN2A2 | 0.00061679 | up-regulated |
| BCL3 | 0.034095196 | up-regulated | POU5F2 | 0.012295065 | up-regulated |
| RC3H2 | 0.00068179 | up-regulated | GTF2IP1 | 0.000201239 | up-regulated |
| PARP10 | 0.006255066 | up-regulated | HS.440088 | 0.017191666 | up-regulated |
| YPEL5 | 0.007901799 | up-regulated | HS.158923 | 0.012910545 | up-regulated |
| ARHGAP26 | 0.009776864 | up-regulated | HS.162932 | 0.009209899 | up-regulated |
| SCYL2 | 0.003842119 | up-regulated | HS.571887 | 0.046306496 | up-regulated |
| SLC10A3 | 0.024003511 | up-regulated | HS.143018 | 0.001886008 | up-regulated |
| LOC642103 | 0.015687897 | up-regulated | LOC728653 | 0.017720281 | up-regulated |
| DGCR2 | 0.000193713 | up-regulated | HS.296031 | 0.007626818 | up-regulated |
| LITAF | 0.044360791 | up-regulated | HS.513580 | 0.023768631 | up-regulated |
| VAMP3 | 0.008499044 | up-regulated | HS.574671 | 0.00027967 | up-regulated |
| TNRC6A | 0.031878998 | up-regulated | HS.5724 | 0.000200867 | up-regulated |
| HARS2 | 0.000785852 | up-regulated | HS.374460 | 0.01206044 | up-regulated |
| USP8 | 0.00149753 | up-regulated | HS.14555 | 0.004895643 | up-regulated |
| PICALM | 0.000232992 | up-regulated | HS.516646 | 0.001261932 | up-regulated |
| PRPF3 | 0.017109967 | up-regulated | HS.572642 | 0.040623306 | up-regulated |
| C20ORF94 | 0.00181839 | up-regulated | HS.483906 | 0.00651007 | up-regulated |
| ITPK1 | 0.003502685 | up-regulated | HS.541352 | 0.013987346 | up-regulated |
| MLL4 | 0.001046549 | up-regulated | HS.566647 | 0.040975079 | up-regulated |
| LMF2 | 0.000262123 | up-regulated | HS.475334 | 0.019171505 | up-regulated |
| CBL | 0.000442835 | up-regulated | HS.386275 | 0.036273434 | up-regulated |
| PACS1 | 0.000269279 | up-regulated | HS.546148 | 0.028949059 | up-regulated |
| GORASP1 | 0.002764334 | up-regulated | HS.573047 | 0.002270761 | up-regulated |
| PPM1B | 0.002668674 | up-regulated | KIAA1881 | 0.027627965 | up-regulated |
| TXNRD1 | 0.019536992 | up-regulated | HS.481659 | 0.045737069 | up-regulated |
| F13A1 | 0.011548907 | up-regulated | HS.485155 | 0.014413033 | up-regulated |
| C6ORF122 | 0.048040231 | up-regulated | HS.184721 | 0.000378453 | up-regulated |
| DKFZP761E198 | 0.000270066 | up-regulated | HS.520591 | 1.91E-05 | up-regulated |
| FLJ43752 | 0.01791551 | up-regulated | HS.551143 | 0.040633097 | up-regulated |
| TIAL1 | 0.02101554 | up-regulated | HS.555181 | 0.003152173 | up-regulated |
| RNF24 | 0.003234641 | up-regulated | HS.452702 | 0.04927015 | up-regulated |
| AGTPBP1 | 0.01791551 | up-regulated | HS.374257 | 0.018345448 | up-regulated |
| PVRL2 | 0.012827254 | up-regulated | HS.99472 | 0.000100028 | up-regulated |
| LOC728499 | 0.033030308 | up-regulated | ATXN7L3 | 0.001147126 | up-regulated |
| CTSA | 0.000283271 | up-regulated | HS.546105 | 0.016691286 | up-regulated |
| CRYZL1 | 0.020419435 | up-regulated | HS.482960 | 0.000262123 | up-regulated |
| WDR26 | 0.030459954 | up-regulated | HS.571741 | 0.01664389 | up-regulated |
| NFKBIZ | 0.035726497 | up-regulated | HS.544637 | 0.000974833 | up-regulated |
| ZFAND3 | 0.012182917 | up-regulated | HS.202577 | 0.02712097 | up-regulated |
| ETS2 | 0.017098808 | up-regulated | HS.352677 | 0.000193713 | up-regulated |
| TRIP12 | 0.019923633 | up-regulated | HS.549989 | 0.029093171 | up-regulated |
| PHF2 | 0.000129483 | up-regulated | HS.571151 | 0.03417992 | up-regulated |
| BAZ2B | 0.000306978 | up-regulated | HS.545615 | 0.034688919 | up-regulated |
| C14ORF159 | 0.033365134 | up-regulated | HS.531457 | 0.028706919 | up-regulated |
| MAGT1 | 0.000582117 | up-regulated | HS.442696 | 0.044155622 | up-regulated |
| TSC22D4 | 0.000109522 | up-regulated | HS.551136 | 0.048233123 | up-regulated |
| PNN | 0.008212686 | up-regulated | HS.254477 | 0.045771554 | up-regulated |
| USP6 | 0.015724501 | up-regulated | HS.572130 | 2.04E-06 | up-regulated |
| ITGA2B | 0.009110998 | up-regulated | HS.22689 | 0.003179044 | up-regulated |
| LOC728452 | 0.017972966 | up-regulated | HS.388347 | 0.047378121 | up-regulated |
| DUSP19 | 0.00925529 | up-regulated | HS.556018 | 0.001077296 | up-regulated |
| TLR5 | 0.011319337 | up-regulated | HS.240801 | 0.024627283 | up-regulated |
| MAP3K1 | 0.014034397 | up-regulated | HS.452445 | 0.042770286 | up-regulated |
| IPO9 | 0.015628162 | up-regulated | HS.580797 | 0.007414419 | up-regulated |
| LOC402176 | 0.006210761 | up-regulated | HS.570343 | 0.001889095 | up-regulated |
| INO80E | 0.022193907 | up-regulated | HS.130036 | 0.001131441 | up-regulated |
| TACC3 | 0.003850064 | up-regulated | HS.193767 | 0.016945964 | up-regulated |
| SELL | 0.034715732 | up-regulated | HS.231861 | 0.00282466 | up-regulated |
| PPP4R1 | 0.001165174 | up-regulated | HS.542993 | 0.004163219 | up-regulated |
| NIN | 0.000809464 | up-regulated | HS.572444 | 0.00732047 | up-regulated |
| XPO1 | 0.038895298 | up-regulated | HS.34558 | 0.000235827 | up-regulated |
| RFX1 | 0.000391196 | up-regulated | FLJ36644 | 0.026746311 | up-regulated |
| RAPGEF6 | 0.001050528 | up-regulated | HS.443185 | 0.018308825 | up-regulated |
| TGFBR2 | 0.001702526 | up-regulated | HS.163752 | 0.023522919 | up-regulated |
| C12ORF35 | 0.038932014 | up-regulated | HS.569411 | 0.021561003 | up-regulated |
| LILRA5 | 0.044581464 | up-regulated | HS.562875 | 0.001007016 | up-regulated |
| MAP3K5 | 0.023229601 | up-regulated | HS.131087 | 0.001993367 | up-regulated |
| OLFML3 | 0.020175899 | up-regulated | ZNF341 | 0.018768914 | up-regulated |
| SSH1 | 0.0458385 | up-regulated | TMX3 | 0.019996931 | up-regulated |
| TNFAIP2 | 0.006164548 | up-regulated | MPHOSPH8 | 0.002504102 | up-regulated |
| RAB33B | 0.018767416 | up-regulated | SENP6 | 0.016223317 | up-regulated |
| PML | 0.015631879 | up-regulated | MBD4 | 0.005989879 | up-regulated |
| EIF4EBP2 | 0.026878878 | up-regulated | TBC1D2B | 0.000442312 | up-regulated |
| PUM2 | 0.000391196 | up-regulated | FAM197Y2 | 0.022116499 | up-regulated |
| LOC400499 | 0.000504375 | up-regulated | PPM1K | 0.007255272 | up-regulated |
| ARFGEF1 | 0.008892149 | up-regulated | HSPC268 | 0.043635492 | up-regulated |
| HDAC7A | 0.004885264 | up-regulated | ALPK1 | 0.002219945 | up-regulated |
| GDE1 | 0.006722858 | up-regulated | LOC440157 | 0.014258957 | up-regulated |
| IL17RA | 0.002270588 | up-regulated | JUNB | 0.026273976 | up-regulated |
| PRUNE | 0.02712097 | up-regulated | ZC3H4 | 0.00083138 | up-regulated |
| IGSF8 | 0.029605035 | up-regulated | OTUD5 | 0.004349861 | up-regulated |
| PHF12 | 0.031942542 | up-regulated | MYST3 | 3.62E-05 | up-regulated |
| CDK5R1 | 0.037844702 | up-regulated | MAP2K4 | 0.00013315 | up-regulated |
| LOC643313 | 0.003956165 | up-regulated | BLZF1 | 0.001852122 | up-regulated |
| LOC644931 | 0.025182529 | up-regulated | CHES1 | 0.007362007 | up-regulated |
| SNRNP70 | 0.037307743 | up-regulated | PIK3C2B | 0.044155622 | up-regulated |
| FMO2 | 0.021613705 | up-regulated | LRAP | 0.001412984 | up-regulated |
| SIK3 | 0.000814011 | up-regulated | RGL2 | 0.003194899 | up-regulated |
| OR2AG1 | 0.048083779 | up-regulated | SLC36A1 | 0.030897683 | up-regulated |
| KIAA1539 | 0.00843064 | up-regulated | SEC16A | 0.000456909 | up-regulated |
| C9ORF164 | 0.001591697 | up-regulated | FOXJ3 | 0.032231907 | up-regulated |
| C1RL | 0.037923818 | up-regulated | DULLARD | 0.001122862 | up-regulated |
| GOLGA3 | 0.02926584 | up-regulated | C14ORF85 | 0.01141311 | up-regulated |
| LOC653103 | 0.029424487 | up-regulated | SHISA5 | 0.033015157 | up-regulated |
| FAM100A | 0.037859132 | up-regulated | CRIPAK | 0.01791551 | up-regulated |
| OGDH | 0.031876469 | up-regulated | FAR1 | 0.014903616 | up-regulated |
| GDI1 | 0.00039386 | up-regulated | LOC440348 | 0.00577578 | up-regulated |
| MAPRE3 | 0.023780888 | up-regulated | MAN2A1 | 2.27E-06 | up-regulated |
| BSDC1 | 0.002364141 | up-regulated | AKIRIN2 | 0.00342208 | up-regulated |
| SELPLG | 0.009777412 | up-regulated | SSTR2 | 0.008894438 | up-regulated |
| DHX38 | 0.047378121 | up-regulated | STK38 | 0.002299675 | up-regulated |
| KLF2 | 0.013510121 | up-regulated | SHOC2 | 0.020419435 | up-regulated |
| YRDC | 0.001050528 | up-regulated | STXBP2 | 0.002697899 | up-regulated |
| GNMT | 0.002698755 | up-regulated | C6ORF111 | 0.005213947 | up-regulated |
| POU2F2 | 0.033365134 | up-regulated | EPOR | 0.022116499 | up-regulated |
| RND2 | 0.033907539 | up-regulated | PFTK1 | 0.005865326 | up-regulated |
| MARK2 | 0.002422734 | up-regulated | RAB8B | 0.006166933 | up-regulated |
| FLII | 0.003900549 | up-regulated | ZNF69 | 0.00100656 | up-regulated |
| BCL6 | 0.02372263 | up-regulated | LOC200030 | 0.047942355 | up-regulated |
| PCMTD1 | 4.66E-05 | up-regulated | NCOR1 | 0.033515158 | up-regulated |
| LOC88523 | 0.00181839 | up-regulated | FLJ10213 | 0.024135312 | up-regulated |
| RBM6 | 0.023951086 | up-regulated | FAM122B | 0.032957014 | up-regulated |
| MYD88 | 0.008076757 | up-regulated | CARD8 | 0.034999584 | up-regulated |
| MAST3 | 0.022861341 | up-regulated | RGS2 | 0.016761795 | up-regulated |
| PPP2R5A | 0.011972065 | up-regulated | ST3GAL4 | 0.049890572 | up-regulated |
| ATG9A | 0.027061551 | up-regulated | BAGE5 | 0.005474371 | up-regulated |
| LOC100129211 | 0.011319337 | up-regulated | SHROOM4 | 0.001297419 | up-regulated |
| BMS1P5 | 0.00741113 | up-regulated | CNOT3 | 0.006806159 | up-regulated |
| CYTH4 | 0.016322582 | up-regulated | TMEM39B | 0.003558767 | up-regulated |
| LOC100132491 | 0.008231904 | up-regulated | PRO0628 | 0.001015781 | up-regulated |
| PLGLB2 | 0.013413434 | up-regulated | MXD1 | 0.008236478 | up-regulated |
| CENPT | 0.014857746 | up-regulated | EPHB4 | 0.0267602 | up-regulated |
| LOC144438 | 0.000442312 | up-regulated | LRRFIP1 | 0.002021312 | up-regulated |
| LOC100132391 | 0.005451881 | up-regulated | PPM2C | 0.01501942 | up-regulated |
| RNY3 | 0.003651729 | up-regulated | GM2A | 0.00776484 | up-regulated |
| CA5BP | 0.025646921 | up-regulated | KIAA0247 | 0.004145168 | up-regulated |
| RAX2 | 0.02712097 | up-regulated | FLJ44124 | 0.04855771 | up-regulated |
| SMCR5 | 0.04672352 | up-regulated | MX2 | 0.021033266 | up-regulated |
| LOC100133998 | 0.047091225 | up-regulated | MYO10 | 0.037882787 | up-regulated |
| LOC100133773 | 0.031240425 | up-regulated | TLE3 | 0.001403632 | up-regulated |
| QRFPR | 0.015172046 | up-regulated | CUTL1 | 3.46E-05 | up-regulated |
| RRP7B | 0.001447426 | up-regulated | FCAR | 0.002696303 | up-regulated |
| LOC100132317 | 0.006200538 | up-regulated | CSF3R | 1.48E-05 | up-regulated |
| TMEM233 | 0.003808242 | up-regulated | PRDM10 | 0.025530881 | up-regulated |
| LOC100132960 | 0.025530881 | up-regulated | OS9 | 0.018335439 | up-regulated |
| LOC440353 | 0.003382483 | up-regulated | HNRNPL | 0.002397384 | up-regulated |
| C7ORF64 | 0.043416646 | up-regulated | KDM6B | 0.014784692 | up-regulated |
| DDX60L | 0.017064411 | up-regulated | AP1G1 | 0.001474709 | up-regulated |
| C9ORF69 | 0.041866252 | up-regulated | CYTH1 | 0.00923961 | up-regulated |
| FAM156B | 0.026496673 | up-regulated | FNDC3B | 0.016794182 | up-regulated |
| SCARNA3 | 0.000955878 | up-regulated | LOC100129441 | 0.000157187 | up-regulated |
| CCDC125 | 0.040013246 | up-regulated | LOC100130353 | 0.016969337 | up-regulated |
| ASAP1IT1 | 8.23E-05 | up-regulated | LOC100129905 | 0.000442312 | up-regulated |
| EHBP1L1 | 0.000206489 | up-regulated | LOC100129094 | 0.029315432 | up-regulated |
| C12ORF51 | 0.017617352 | up-regulated | LOC100128084 | 0.016013382 | up-regulated |
| DENND4B | 3.46E-05 | up-regulated | LOC100130492 | 0.029721412 | up-regulated |
| FBRS | 3.55E-06 | up-regulated | LOC440366 | 0.020987405 | up-regulated |
| ZNF860 | 0.016484525 | up-regulated | LOC727908 | 0.000168457 | up-regulated |
| LRRC37B2 | 0.016761795 | up-regulated | LOC646438 | 0.042559067 | up-regulated |
| MSL1 | 0.001333182 | up-regulated | LOC100133876 | 0.017971745 | up-regulated |
| LOC100133516 | 0.003483998 | up-regulated | LOC100131541 | 0.008443134 | up-regulated |
| SNORA22 | 0.008614577 | up-regulated | LOC642623 | 0.024003511 | up-regulated |
| LOC100132247 | 0.000609246 | up-regulated | LOC644518 | 0.002285814 | up-regulated |
| LOC100190986 | 7.66E-05 | up-regulated | LOC255167 | 0.033015157 | up-regulated |
| LOC100134734 | 0.006623456 | up-regulated | LOC100132526 | 0.035987389 | up-regulated |
| LOC100233209 | 0.004394546 | up-regulated | LOC645452 | 0.035549026 | up-regulated |
| LOC100134634 | 0.010052509 | up-regulated | LOC645822 | 0.036499559 | up-regulated |
| LOC727758 | 0.000498068 | up-regulated | LOC100133177 | 0.000442312 | up-regulated |
| LOC730387 | 0.021041326 | up-regulated | LOC728787 | 0.004798989 | up-regulated |
| LOC100134241 | 0.003365652 | up-regulated | LOC730284 | 0.03044958 | up-regulated |
| MAPK1IP1L | 0.032884691 | up-regulated | LOC728362 | 0.00225393 | up-regulated |
| LOC100134530 | 0.044228304 | up-regulated | LOC728903 | 0.011548907 | up-regulated |
| LOC100128274 | 0.008763802 | up-regulated | LOC730060 | 0.042990751 | up-regulated |
| LOC100128505 | 0.028706919 | up-regulated | MGC26356 | 0.021286047 | up-regulated |
| LOC100129362 | 0.004349861 | up-regulated | LOC731542 | 0.009773908 | up-regulated |
| LOC100129502 | 0.038660223 | up-regulated | LOC100130516 | 0.01349233 | up-regulated |
| LOC100130835 | 0.004508969 | up-regulated | LOC728105 | 0.044208743 | up-regulated |
| LOC100130276 | 0.033051242 | up-regulated | KIAA1731 | 0.000847239 | up-regulated |
| ARAP1 | 0.001556492 | up-regulated | HIATL2 | 0.023109155 | up-regulated |
| LOC100130367 | 0.032794152 | up-regulated | LOC100128288 | 0.00353637 | up-regulated |
| LOC100133607 | 0.001814179 | up-regulated | LOC100133923 | 0.010403531 | up-regulated |
| LOC100131696 | 0.042744391 | up-regulated | LPAR2 | 0.002555415 | up-regulated |
| LOC646547 | 0.027546871 | up-regulated | LOC100129550 | 0.036482748 | up-regulated |
| LOC100190938 | 0.020080349 | up-regulated | UBXN2B | 0.006261863 | up-regulated |
| LOC401098 | 0.008361638 | up-regulated | FAM175A | 0.013999039 | up-regulated |
| UBA1 | 0.004062509 | up-regulated | LOC100132585 | 0.046316854 | up-regulated |
| LOC729793 | 0.023815878 | up-regulated | LOC730313 | 0.005917823 | up-regulated |
| LOC729978 | 0.024677692 | up-regulated | PTAR1 | 0.001853427 | up-regulated |
| LOC729040 | 0.000955878 | up-regulated | CCNJL | 0.019779801 | up-regulated |
| MIR1267 | 0.037862259 | up-regulated | FBXL11 | 0.003128198 | up-regulated |

**Supplement Table S10** The list of drugs for AS treatment and drug targets.

| **Drug name** | **Target name** | **Target ID** |
| --- | --- | --- |
| Etanercept | Tumor necrosis factor | 7124 |
| Etanercept | Tumor necrosis factor receptor superfamily member 1B | 7133 |
| Etanercept | High affinity immunoglobulin gamma Fc receptor I | 2209 |
| Etanercept | Low affinity immunoglobulin gamma Fc region receptor III-A | 2214 |
| Etanercept | Low affinity immunoglobulin gamma Fc region receptor II-a | 2212 |
| Etanercept | Low affinity immunoglobulin gamma Fc region receptor II-b | 2213 |
| Etanercept | Low affinity immunoglobulin gamma Fc region receptor II-c | 9103 |
| Etanercept | Lymphotoxin-alpha | 4049 |
| Etanercept | Low affinity immunoglobulin gamma Fc region receptor III-B | 2215 |
| Etanercept | Complement C1s subcomponent | 716 |
| Etanercept | Complement C1r subcomponent | 715 |
| Etanercept | Complement C1q subcomponent subunit A | 712 |
| Etanercept | Complement C1q subcomponent subunit B | 713 |
| Etanercept | Complement C1q subcomponent subunit C | 714 |
| Etanercept | Prostaglandin G/H synthase 2 | 5743 |
| Phenylbutazone | Prostaglandin G/H synthase 2 | 5743 |
| Phenylbutazone | Prostacyclin synthase | 5740 |
| Phenylbutazone | Prostaglandin G/H synthase 1 | 5742 |
| Phenylbutazone | Cytochrome P450 2C9 | 1559 |
| Phenylbutazone | Cytochrome P450 3A4 | 1576 |
| Phenylbutazone | Solute carrier family 22 member 6 | 9356 |
| Phenylbutazone | Solute carrier family 22 member 8 | 9356 |
| Phenylbutazone | Solute carrier family 22 member 11 | 55867 |
| golimumab | Tumor necrosis factor | 7124 |
| Adalimumab | Tumor necrosis factor | 7124 |
| Adalimumab | Low affinity immunoglobulin gamma Fc region receptor III-B | 2215 |
| Adalimumab | Complement C1r subcomponent | 715 |
| Adalimumab | Complement C1q subcomponent subunit A | 712 |
| Adalimumab | Complement C1q subcomponent subunit B | 713 |
| Adalimumab | Complement C1q subcomponent subunit C | 714 |
| Adalimumab | Low affinity immunoglobulin gamma Fc region receptor III-A | 2214 |
| Adalimumab | Complement C1s subcomponent | 716 |
| Adalimumab | High affinity immunoglobulin gamma Fc receptor I | 2209 |
| Adalimumab | Low affinity immunoglobulin gamma Fc region receptor II-a | 2212 |
| Adalimumab | Low affinity immunoglobulin gamma Fc region receptor II-b | 2213 |
| Adalimumab | Low affinity immunoglobulin gamma Fc region receptor II-c | 9103 |
| Etoricoxib | Prostaglandin G/H synthase 2 | 5743 |
| Etoricoxib | Cytochrome P450 3A4 | 1576 |
| Etoricoxib | Cytochrome P450 2C9 | 1559 |
| Etoricoxib | Cytochrome P450 2D6 | 1565 |
| Etoricoxib | Cytochrome P450 1A2 | 1544 |
| Etoricoxib | Cytochrome P450 2C19 | 1557 |
| Etoricoxib | Cytochrome P450 2E1 | 1571 |
| Secukinumab | Interleukin-17A | 3605 |
| Infliximab | Tumor necrosis factor | 7124 |
| Ketoprofen | Prostaglandin G/H synthase 1 | 5742 |
| Ketoprofen | Prostaglandin G/H synthase 2 | 5743 |
| Ketoprofen | C-X-C chemokine receptor type 1 | 3577 |
| Ketoprofen | Cytochrome P450 2C9 | 1559 |
| Ketoprofen | Cytochrome P450 2C8 | 1558 |
| Ketoprofen | Serum albumin | 213 |
| Ketoprofen | Multidrug resistance-associated protein 4 | 10257 |
| Ketoprofen | Solute carrier organic anion transporter family member 1A2 | 6579 |
| Ketoprofen | Solute carrier family 22 member 6 | 9356 |
| Ketoprofen | Solute carrier family 22 member 8 | 9376 |
| Ketoprofen | Solute carrier family 22 member 11 | 55867 |
| Ketoprofen | Solute carrier family 22 member 7 | 10864 |
| Sulindac | Prostaglandin G/H synthase 2 | 5743 |
| Sulindac | Prostaglandin G/H synthase 1 | 5742 |
| Sulindac | Aldose reductase | 231 |
| Sulindac | Mitogen-activated protein kinase 3 | 5595 |
| Sulindac | Peroxisome proliferator-activated receptor delta | 5467 |
| Sulindac | Prostaglandin D2 receptor 2 | 11251 |
| Sulindac | Cytochrome P450 1A1 | 1543 |
| Sulindac | Cytochrome P450 1A2 | 1544 |
| Sulindac | Serum albumin | 213 |
| Sulindac | Solute carrier family 22 member 6 | 9356 |
| Diclofenac | Prostaglandin G/H synthase 2 | 5743 |
| Diclofenac | Prostaglandin G/H synthase 1 | 5742 |
| Diclofenac | Arachidonate 5-lipoxygenase | 240 |
| Diclofenac | Sodium channel protein type 4 subunit alpha | 6329 |
| Diclofenac | Acid-sensing ion channel 1 | 41 |
| Diclofenac | Potassium voltage-gated channel subfamily KQT member 2 | 3785 |
| Diclofenac | Potassium voltage-gated channel subfamily KQT member 3 | 3786 |
| Diclofenac | Phospholipase A2, membrane associated | 5320 |
| Diclofenac | Cytochrome P450 2C9 | 1559 |
| Diclofenac | Cytochrome P450 2C19 | 1557 |
| Diclofenac | Cytochrome P450 1A2 | 1544 |
| Diclofenac | Cytochrome P450 2C8 | 1558 |
| Diclofenac | UDP-glucuronosyltransferase 1-1 | 54658 |
| Diclofenac | UDP-glucuronosyltransferase 2B7 | 7364 |
| Diclofenac | Prostaglandin G/H synthase 1 | 5742 |
| Diclofenac | Cytochrome P450 3A4 | 1576 |
| Diclofenac | Cytochrome P450 1A1 | 1543 |
| Diclofenac | Cytochrome P450 2B6 | 1555 |
| Diclofenac | Cytochrome P450 2C18 | 1562 |
| Diclofenac | Cytochrome P450 2E1 | 1571 |
| Diclofenac | Transthyretin | 7276 |
| Diclofenac | Serum albumin | 213 |
| Diclofenac | Multidrug resistance-associated protein 4 | 10257 |
| Diclofenac | Multidrug resistance protein 1 | 5243 |
| Diclofenac | Multidrug resistance-associated protein 1 | 4363 |
| Diclofenac | Solute carrier family 22 member 6 | 9356 |
| Diclofenac | Solute carrier family 22 member 8 | 9376 |
| Diclofenac | Solute carrier organic anion transporter family member 1C1 | 53919 |
| Diclofenac | Solute carrier family 22 member 11 | 55867 |
| Indomethacin | Prostaglandin G/H synthase 1 | 5742 |
| Indomethacin | Prostaglandin G/H synthase 2 | 5743 |
| Indomethacin | Phospholipase A2, membrane associated | 5320 |
| Indomethacin | Prostaglandin reductase 2 | 145482 |
| Indomethacin | Peroxisome proliferator-activated receptor gamma | 5468 |
| Indomethacin | Lactoylglutathione lyase | 2739 |
| Indomethacin | Prostaglandin D2 receptor 2 | 11251 |
| Indomethacin | Peroxisome proliferator-activated receptor alpha | 5465 |
| Indomethacin | Cytochrome P450 2C19 | 1557 |
| Indomethacin | Cytochrome P450 2C9 | 1559 |
| Indomethacin | Liver carboxylesterase 1 | 1066 |
| Indomethacin | UDP-glucuronosyltransferase 1-9 | 54600 |
| Indomethacin | UDP-glucuronosyltransferase 1-1 | 54658 |
| Indomethacin | UDP-glucuronosyltransferase 2B7 | 7364 |
| Indomethacin | Serum albumin | 213 |
| Indomethacin | Canalicular multispecific organic anion transporter 2 | 8714 |
| Indomethacin | Multidrug resistance-associated protein 4 | 10257 |
| Indomethacin | Multidrug resistance-associated protein 6 | 368 |
| Indomethacin | Multidrug resistance protein 1 | 105369239 |
| Indomethacin | Multidrug resistance-associated protein 1 | 4363 |
| Indomethacin | Solute carrier organic anion transporter family member 1A2 | 6579 |
| Indomethacin | Solute carrier family 22 member 6 | 9356 |
| Indomethacin | Solute carrier family 22 member 8 | 9376 |
| Indomethacin | Canalicular multispecific organic anion transporter 1 | 1244 |
| Indomethacin | ATP-binding cassette sub-family C member 11 | 85320 |
| Indomethacin | Solute carrier family 22 member 11 | 55867 |
| Indomethacin | Sodium/bile acid cotransporter | 6554 |
| Indomethacin | Solute carrier family 22 member 7 | 10864 |
| Celecoxib | Prostaglandin G/H synthase 2 | 5743 |
| Celecoxib | 3-phosphoinositide-dependent protein kinase 1 | 5170 |
| Celecoxib | Cytochrome P450 2C9 | 1559 |
| Celecoxib | Cytochrome P450 3A4 | 1576 |
| Celecoxib | Cytochrome P450 2D6 | 1565 |
| Celecoxib | Serum albumin | 213 |
| Celecoxib | Alpha-1-acid glycoprotein 1 | 5004 |
| Celecoxib | Multidrug resistance-associated protein 4 | 10257 |
| Prednisolone | Glucocorticoid receptor | 2908 |
| Prednisolone | Cytochrome P450 3A4 | 1576 |
| Prednisolone | Cytochrome P450 2A6 | 1548 |
| Prednisolone | Corticosteroid-binding globulin | 866 |
| Prednisolone | Solute carrier organic anion transporter family member 1A2 | 6579 |
| Prednisolone | Multidrug resistance protein 1 | 105369239 |
| Tofacitinib | Tyrosine-protein kinase JAK2 | 3717 |
| Tofacitinib | Tyrosine-protein kinase JAK1 | 3716 |
| Tofacitinib | Tyrosine-protein kinase JAK3 | 3718 |
| Tofacitinib | Cytochrome P450 3A4 | 1576 |
| Tofacitinib | Cytochrome P450 2C19 | 1557 |
| Tofacitinib | Serum albumin | 213 |
| Naproxen | Prostaglandin G/H synthase 2 | 5743 |
| Naproxen | Prostaglandin G/H synthase 1 | 5742 |
| Naproxen | Cytochrome P450 2C9 | 1559 |
| Naproxen | Cytochrome P450 1A2 | 1544 |
| Naproxen | Cytochrome P450 2C8 | 1558 |
| Naproxen | UDP-glucuronosyltransferase 1-1 | 54658 |
| Naproxen | UDP-glucuronosyltransferase 2B7 | 7364 |
| Naproxen | Serum albumin | 213 |
| Naproxen | Solute carrier organic anion transporter family member 1A2 | 6579 |
| Naproxen | Solute carrier family 22 member 6 | 9356 |
| Ibuprofen | Prostaglandin G/H synthase 2 | 5743 |
| Ibuprofen | Prostaglandin G/H synthase 1 | 5742 |
| Ibuprofen | Apoptosis regulator Bcl-2 | 596 |
| Ibuprofen | Thrombomodulin | 7056 |
| Ibuprofen | Tissue-type plasminogen activator | 5327 |
| Ibuprofen | Fatty acid-binding protein, intestinal | 2169 |
| Ibuprofen | Peroxisome proliferator-activated receptor gamma | 5468 |
| Ibuprofen | Cystic fibrosis transmembrane conductance regulator | 1080 |
| Ibuprofen | Cytochrome P450 2C9 | 1559 |
| Ibuprofen | Cytochrome P450 2C8 | 1558 |
| Ibuprofen | Cytochrome P450 2C19 | 1557 |
| Ibuprofen | UDP-glucuronosyltransferase 1-1 | 54658 |
| Ibuprofen | UDP-glucuronosyltransferase 1-3 | 54659 |
| Ibuprofen | UDP-glucuronosyltransferase 1-9 | 54600 |
| Ibuprofen | UDP-glucuronosyltransferase 2B4 | 7363 |
| Ibuprofen | UDP-glucuronosyltransferase 2B7 | 7364 |
| Ibuprofen | Prostaglandin G/H synthase 1 | 5742 |
| Ibuprofen | Prostaglandin G/H synthase 2 | 5743 |
| Ibuprofen | Serum albumin | 213 |
| Ibuprofen | Solute carrier organic anion transporter family member 2B1 | 11309 |
| Ibuprofen | Multidrug resistance protein 1 | 105369239 |
| Ibuprofen | Multidrug resistance-associated protein 4 | 10257 |
| Ibuprofen | Multidrug resistance-associated protein 1 | 4363 |
| Ibuprofen | Solute carrier organic anion transporter family member 1A2 | 6579 |
| Ibuprofen | Solute carrier family 22 member 6 | 9356 |
| Ibuprofen | Solute carrier family 22 member 8 | 9376 |
| Ibuprofen | Solute carrier family 22 member 11 | 55867 |
| Acetylsalicylic acid | Prostaglandin G/H synthase 1 | 5742 |
| Acetylsalicylic acid | Prostaglandin G/H synthase 2 | 5743 |
| Acetylsalicylic acid | Aldo-keto reductase family 1 member C1 | 1645 |
| Acetylsalicylic acid | 5'-AMP-activated protein kinase catalytic subunit alpha-1 | 5562 |
| Acetylsalicylic acid | Endothelin-1 receptor | 1909 |
| Acetylsalicylic acid | Inhibitor of nuclear factor kappa-B kinase subunit beta | 3551 |
| Acetylsalicylic acid | Cellular tumor antigen p53 | 7157 |
| Acetylsalicylic acid | 78 kDa glucose-regulated protein | 283234 |
| Acetylsalicylic acid | Ribosomal protein S6 kinase alpha-3 | 6197 |
| Acetylsalicylic acid | NF-kappa-B inhibitor alpha | 4792 |
| Acetylsalicylic acid | Nuclear factor NF-kappa-B p100 subunit | 4791 |
| Acetylsalicylic acid | Cytochrome P450 2C19 | 1557 |
| Acetylsalicylic acid | Cytochrome P450 2C8 | 1558 |
| Acetylsalicylic acid | Cytochrome P450 2C9 | 1559 |
| Acetylsalicylic acid | Serum albumin | 213 |
| Acetylsalicylic acid | Solute carrier family 22 member 6 | 9356 |
| Acetylsalicylic acid | Multidrug resistance protein 1 | 5243 |
| Acetylsalicylic acid | Solute carrier family 22 member 7 | 10864 |
| Flurbiprofen | Prostaglandin G/H synthase 1 | 5742 |
| Flurbiprofen | Prostaglandin G/H synthase 2 | 5743 |
| Flurbiprofen | Cytochrome P450 2C9 | 1559 |
| Flurbiprofen | UDP-glucuronosyltransferase 2B7 | 7364 |
| Flurbiprofen | UDP-glucuronosyltransferase 1-1 | 54658 |
| Flurbiprofen | UDP-glucuronosyltransferase 1-3 | 54659 |
| Flurbiprofen | UDP-glucuronosyltransferase 1-9 | 54600 |
| Flurbiprofen | UDP-glucuronosyltransferase 2B4 | 7363 |
| Flurbiprofen | Serum albumin | 213 |
| Flurbiprofen | Multidrug resistance-associated protein 4 | 10257 |
| Flurbiprofen | Solute carrier family 22 member 6 | 9356 |

**Supplement Table S11** AS Disease genes and supplied database.

| **Gene** **Symbol** | **Gene ID** | **Database** | **Gene Symbol** | **Gene ID** | **Database** | **Gene Symbol** | **Gene ID** | **Database** |
| --- | --- | --- | --- | --- | --- | --- | --- | --- |
| ABCF1 | 23 | GAD | IL1B | 3553 | GAD | PSMB8 | 5696 | GAD |
| ALPL | 249 | GAD | IL1F10 | 84639 | GAD | PSMB9 | 5698 | GAD |
| ANKH | 56172 | GAD | IL1F5 | 26525 | GAD | RPL13AP14 | 100271278 | GAD |
| ANO6 | 196527 | GAD | IL1F6 | 27179 | GAD | RUNX3 | 864 | GAD |
| ANTXR2 | 118429 | GAD | IL1F7 | 27178 | GAD | SOD2 | 6648 | GAD |
| ARTS-1 | 51752 | GAD | IL1F8 | 27177 | GAD | STAT3 | 6774 | GAD |
| ASPN | 54829 | GAD | IL1F9 | 56300 | GAD | TAP1 | 6890 | GAD |
| B3GNT2 | 10678 | GAD | IL1R1 | 3554 | GAD | TAP2 | 6891 | GAD |
| C10orf112 | 340895 | GAD | IL1R2 | 7850 | GAD | TGFB1 | 7040 | GAD |
| CARD15 | 64127 | GAD | IL1RN | 3557 | GAD | TIMP1 | 7076 | GAD |
| CARD9 | 64170 | GAD | IL23R | 149233 | GAD | TLR4 | 7099 | GAD |
| CD14 | 929 | GAD | IL4 | 3565 | GAD | TNAP | 445341 | GAD |
| COL6A1 | 1291 | GAD | IL6 | 3569 | GAD | TNF | 7124 | GAD |
| CSF2RB | 1439 | GAD | JAK2 | 3717 | GAD | TNFRSF1A | 7132 | GAD |
| CTLA4 | 1493 | GAD | KIF21B | 23046 | GAD | TNFRSF1B | 7133 | GAD |
| CYP1A1 | 1543 | GAD | KIR2DL1 | 3802 | GAD | TRADD | 8717 | GAD |
| CYP2D6 | 1565 | GAD | KIR2DL2 | 3803 | GAD | UBE2E3 | 10477 | GAD |
| DAB2 | 1601 | GAD | KIR2DL3 | 3804 | GAD | VDR | 7421 | GAD |
| DHFRP2 | 729816 | GAD | KIR2DL4 | 3805 | GAD | VEGFA | 7422 | GAD |
| ERAP1 | 51752 | GAD | KIR2DL5A | 57292 | GAD | ACVR1 | 90 | OMIM |
| ERAP2 | 64167 | GAD | KIR2DP1 | 554300 | GAD | B2M | 567 | OMIM |
| FCRL3 | 115352 | GAD | KIR2DS1 | 3806 | GAD | RUNX3 | 864 | OMIM |
| FCRL5 | 83416 | GAD | KIR2DS2 | 100132285 | GAD | CYP2D6 | 1565 | OMIM |
| FLJ37396 | 389422 | GAD | KIR2DS3 | 3808 | GAD | HLA-B | 3106 | OMIM |
| FLJ45139 | 400867 | GAD | KIR2DS4 | 3809 | GAD | IL12B | 3593 | OMIM |
| GNL1 | 2794 | GAD | KIR2DS5 | 3810 | GAD | LTBR | 4055 | OMIM |
| HLA-A | 3105 | GAD | KIR3DL1 | 3811 | GAD | PTGER4 | 5734 | OMIM |
| HLA-B | 3106 | GAD | KIR3DL2 | 3812 | GAD | PSF1 | 6890 | OMIM |
| HLA-C | 3107 | GAD | KIR3DL3 | 115653 | GAD | PSF2 | 6891 | OMIM |
| HLA-DPB1 | 3115 | GAD | KIR3DS1 | 3813 | GAD | TNF-alpha | 7124 | OMIM |
| HLA-DQA1 | 3117 | GAD | KPNB1 | 3837 | GAD | TNFRSF1A | 7132 | OMIM |
| HLA-DQB1 | 3119 | GAD | LTBR | 4055 | GAD | TNFRSF1B | 7133 | OMIM |
| HLA-DQB1 | 3120 | GAD | MEFV | 4210 | GAD | IL1R2 | 7850 | OMIM |
| HLA-DRA | 3122 | GAD | MICA | 100507436 | GAD | APRIL | 8741 | OMIM |
| HLA-DRB1 | 3123 | GAD | MMP3 | 4314 | GAD | TBKBP1 | 9755 | OMIM |
| HLA-E | 3133 | GAD | MSX2 | 4488 | GAD | PSF1 | 9837 | OMIM |
| HLA-S | 267015 | GAD | MTCO1P2 | 326603 | GAD | APRIL | 10541 | OMIM |
| HSPA1A | 3303 | GAD | NFKB1 | 4790 | GAD | BLYS | 10673 | OMIM |
| HSPA1B | 3304 | GAD | NFKBIA | 4792 | GAD | PSF2 | 51659 | OMIM |
| HSPA1L | 3305 | GAD | NOD2 | 64127 | GAD | ERAP1 | 51752 | OMIM |
| HSPA2 | 3306 | GAD | PADI4 | 23569 | GAD | CARD9 | 64170 | OMIM |
| IL10 | 3586 | GAD | PDCD1 | 5133 | GAD | ANTXR2 | 118429 | OMIM |
| IL12B | 3593 | GAD | PRR3 | 80742 | GAD | IL23R | 149233 | OMIM |
| IL1A | 3552 | GAD |  |  |  |  |  |  |
